# Supplementary material for: The role of species ecology in predicting Toxoplasma gondii prevalence in wild and domesticated mammals globally
Source: PLoS Pathog. 2024 Jan 10;20(1):e1011908. doi: 10.1371/journal.ppat.1011908 (PMC10805296; doi:10.1371/journal.ppat.1011908)
Supplement: S1 Table — Numbers of sampled individuals and species are provided in parentheses. (DOCX) [file ppat.1011908.s001.docx]

**S1 Table** Prevalence of *T. gondii* with 95% confidence intervals estimated at the taxonomic family level for free-ranging wild mammal populations compiled across 485 publications. Numbers of sampled individuals and species are provided in parentheses.

| Order | Taxonomic family | Prevalence (95%) |
| --- | --- | --- |
| Carnivora  (n= 43,090, Sps =112) | Canidae  (n = 12,609) | 44.47 (43.6-45.34) |
|  | Eupleridae  (n = 110) | 57.27 (47.48-66.66) |
|  | Felidae  (n = 4,313) | 55.76 (54.26-57.25) |
|  | Herpestidae  (n = 608) | 29.61 (26-33.41) |
|  | Hyaenidae  (n = 200) | 68.5 (61.57-74.87) |
|  | Mephitidae  (n = 1391) | 25.95 (23.67-28.34) |
|  | Mustelidae  (n = 5465) | 40.82 (39.52-42.14) |
|  | Odobenidae  (n = 143) | 10.49 (5.99-16.71) |
|  | Otariidae  (n = 1836) | 5.72 (4.7-6.88) |
|  | Phocidae  (n = 3997) | 9.46 (8.57-10.41) |
|  | Procyonidae  (n = 5315) | 32.87 (31.61-34.15) |
|  | Ursidae  (n = 7052) | 40.7 (39.55-41.86) |
|  | Viverridae  (n = 51) | 58.82 (44.17-72.42) |
| Cetartiodactyla  (n= 57,695, Sps =89) | Antilocapridae  (n = 63) | 4.76 (0.99-13.29) |
|  | Balaenidae  (n = 2) | 50 (1.26-98.74) |
|  | Balaenopteridae  (n = 205) | 0.49 (0.01-2.69) |
|  | Bovidae  (n = 6131) | 10.52 (9.75-11.32) |
|  | Cervidae  (n = 25,129) | 20.35 (19.85-20.85) |
|  | Delphinidae  (n = 430) | 24.42 (20.43-28.76) |
|  | Eschrichtiidae  (n = 21) | 9.52 (1.17-30.38) |
|  | Giraffidae  (n = 20) | 30 (11.89-54.28) |
|  | Iniidae  (n = 293) | 55.97 (50.08-61.74) |
|  | Kogiidae  (n = 9) | 0 (0-33.63) |
|  | Monodontidae  (n = 485) | 38.14 (33.8-42.63) |
|  | Phocoenidae  (n = 91) | 2.2 (0.27-7.71) |
|  | Physeteridae  (n = 3) | 0 (0-70.76) |
|  | Suidae  (n = 24,591) | 24.91 (24.37-25.45) |
|  | Tayassuidae  (n = 220) | 62.73 (55.97-69.13) |
|  | Ziphiidae  (n = 2) | 0 (0-84.19) |
| Chiroptera  (n= 4463, Sps =96) | Emballonuridae  (n = 24) | 45.83 (25.55-67.18) |
|  | Hipposideridae  (n = 437) | 8.47 (6.03-11.48) |
|  | Megadermatidae  (n = 74) | 32.43 (22-44.32) |
|  | Miniopteridae  (n = 368) | 37.23 (32.27-42.39) |
|  | Molossidae  (n = 1,335) | 8.01 (6.61-9.6) |
|  | Mormoopidae  (n = 6) | 0 (0-45.93) |
|  | Noctilionidae  (n = 6) | 0 (0-45.93) |
|  | Phyllostomidae  (n = 839) | 13.59 (11.34-16.09) |
|  | Pteropodidae  (n = 285) | 8.07 (5.18-11.86) |
|  | Rhinolophidae  (n = 298) | 6.38 (3.88-9.78) |
|  | Vespertilionidae  (n = 791) | 7.96 (6.17-10.08) |
| Cingulata  (n = 239, Sps = 6) | Chlamyphoridae  (n = 41) | 7.32 (1.54-19.92) |
|  | Dasypodidae  (n = 198) | 31.82 (25.4-38.8) |
| Dasyuromorphia  (n = 74, Sps = 4) | Dasyuridae  (n = 74) | 41.89 (30.51-53.94) |
| Didelphimorphia  (n = 1568, Sps = 12) | Didelphidae  (n = 1568) | 15.63 (13.86-17.52) |
| Diprotodontia  (n = 1913, Sps = 9) | Macropodidae  (n = 1292) | 11.61 (9.91-13.48) |
|  | Phalangeridae  (n = 374) | 4.01 (2.26-6.53) |
|  | Phascolarctidae  (n = 157) | 0 (0-2.32) |
|  | Potoroidae  (n = 67) | 0 (0-5.36) |
|  | Vombatidae  (n = 23) | 26.09 (10.23-48.41) |
| Eulipotyphla  (n = 1222, Sps = 20) | Erinaceidae  (n = 118) | 22.03 (14.93-30.59) |
|  | Soricidae  (n = 969) | 5.99 (4.58-7.67) |
|  | Talpidae  (n = 135) | 6.67 (3.09-12.28) |
| Lagomorpha  (n = 9160, Sps = 12) | Leporidae  (n = 9,138) | 9.62 (9.02-10.24) |
|  | Ochotonidae  (n = 22) | 9.09 (1.12-29.16) |
| Paucituberculata  (n = 5, Sps = 1) | Caenolestidae  (n = 5) | 0 (0-52.18) |
| Peramelemorphia  (n = 314, Sps = 2) | Peramelidae  (n = 314) | 4.78 (2.7-7.76) |
| Perissodactyla  (n = 35, Sps = 4) | Equidae  (n = 10) | 90 (55.5-99.75) |
|  | Rhinocerotidae  (n = 13) | 30.77 (9.09-61.43) |
|  | Tapiridae  (n = 12) | 50 (21.09-78.91) |
| Pilosa  (n = 146, Sps = 4) | Bradypodidae  (n = 6) | 0 (0-45.93) |
|  | Megalonychidae  (n = 100) | 0 (0-3.62) |
|  | Myrmecophagidae  (n = 40) | 42.5 (27.04-59.11) |
| Primates  (n = 1043, Sps = 26) | Atelidae  (n = 307) | 13.68 (10.04-18.04) |
|  | Callitrichidae  (n = 296) | 5.07 (2.86-8.22) |
|  | Cebidae  (n = 152) | 18.42 (12.6-25.51) |
|  | Cercopithecidae  (n = 272) | 27.57 (22.35-33.29) |
|  | Lemuridae  (n = 10) | 10 (0.25-44.5) |
|  | Pitheciidae  (n = 6) | 0 (0-45.93) |
| Proboscidea  (n = 39, Sps = 1) | Elephantidae  (n = 39) | 10.26 (2.87-24.22) |
| Rodentia  (n = 27,660, Sps = 133) | Castoridae  (n = 116) | 5.17 (1.92-10.92) |
|  | Caviidae  (n = 116) | 59.81 (54.18-65.26) |
|  | Cricetidae  (n = 9,160) | 7.25 (6.73-7.8) |
|  | Cuniculidae  (n = 60) | 60 (46.54-72.44) |
|  | Dasyproctidae  (n = 150) | 18.67 (12.78-25.84) |
|  | Dipodidae  (n = 6) | 0 (0-45.93) |
|  | Echimyidae  (n = 207) | 2.42 (0.79-5.55) |
|  | Erethizontidae  (n = 46) | 2.17 (0.06-11.53) |
|  | Gliridae  (n = 44) | 0 (0-8.04) |
|  | Heteromyidae  (n = 41) | 9.76 (2.72-23.13) |
|  | Muridae  (n = 15,049) | 4.2 (3.88-4.53) |
|  | Myocastoridae  (n = 472) | 27.75 (23.76-32.03) |
|  | Nesomyidae  (n = 66) | 24.24 (14.54-36.36) |
|  | Sciuridae  (n = 1927) | 4.2 (3.35-5.20) |
| Sirenia  (n = 640, Sps = 2) | Dugongidae  (n = 114) | 4.39 (1.44-9.94) |
|  | Trichechidae  (n = 526) | 2.28 (1.18-3.95) |
