# Supplementary material for: The role of species ecology in predicting Toxoplasma gondii prevalence in wild and domesticated mammals globally
Source: PLoS Pathog. 2024 Jan 10;20(1):e1011908. doi: 10.1371/journal.ppat.1011908 (PMC10805296; doi:10.1371/journal.ppat.1011908)
Supplement: S2 Table — The numbers of sampled individuals are provided in parentheses. (DOCX) [file ppat.1011908.s002.docx]

**S2 Table** Prevalence of *T. gondii* with 95% confidence intervals estimated from a compilation of 540 published global studies for domesticated and free-ranging populations of Bovidae and Suidae. The numbers of sampled individuals are provided in parentheses.

| **Continent** | **Domestic** | **Wild** | **Continental** |
| --- | --- | --- | --- |
| **a) Bovidae** |  |  |  |
| Africa | 27.8 (27.2 – 28.5)  (n = 20, 232) | 37.18 (32.1– 42.5)  (n = 347) | 27.98 (27.4 – 28.6)  (n= 20, 579) |
| Asia | 17.5 (17.2 – 17.8)  (n = 63, 994) | 4.3(3.1 – 5.8)  (n = 951) | 17.3 (17.0 – 17.6)  (n = 64, 945) |
| Europe | 40.3 (39.9 – 40.8)  (n = 54, 246) | 8.3 (6.8 – 9.9)  (n = 1, 233) | 39.6 (39.2 – 40.0)  (n= 55, 479) |
| Latin America | 41.1 (36.2-46.1)  (n = 392) |  |  |
| North America | 23.0 (21.6-24.5)  (n = 3, 358) | 10.4 (9.4-11.4)  (n = 3, 459) | 16.6 (15.7-17.5)  (n = 6, 817) |
| Oceania | 84.0 (82.4-85.5)  (n = 2, 284) |  |  |
| South America | 32.2 (31.6-32.8)  (n = 23, 048) |  |  |
| *Total* | 29.2 (29.0-29.5)  (n = 335, 108) | 10.5 (9.7-11.3)  (n = 11, 980) | 28.6 (28.4-28.8)  (n = 347, 088) |
| **b) Suidae** |  |  |  |
|  | **Domestic** | **Wild** | **Continental** |
| Africa | 32.4 (30.9-33.9)  (n = 3, 859) | 29.4 (26.5-32.3)  (n = 980) | 31.8 (30.5-33.1)  (n = 4, 839) |
| Asia | 29.5 (29.2-29.9)  (n = 60, 491) | 23.2 (21.7-24.7)  (n = 3,008) | 29.2 (28.9-29.6)  (n = 63, 499) |
| Europe | 12.2 (12-12.5)  (n = 82, 384) | 26.8 (26.1-27.4)  (n = 17, 614) | 14.8 (14.6-15)  (n = 99, 998) |
| Latin America | 43.5 (39.1-48.0)  (n = 496) |  |  |
| North America | 13.9 (13.6-14.2)  (n = 58, 073) | 11.2 (10.1-12.5)  (n = 2, 429) | 13.8 (13.5-14.05)  (n = 60, 502) |
| Oceania | 2.0 (0.05-10.9)  (n = 49) |  |  |
| South America | 22.1 (21.4-22.9)  (n = 12, 789) | 28.0 (24.3-31.9)  (n = 560) | 22.38 (21.67-23.09)  (n = 13, 349) |
| *Total* | 18.5 (18.3 -18.6)  (n = 436, 232) | 24.9 (24.4 -25.4)  (n = 49, 182) | 19.1 (19.1-19.3)  (n = 485, 464) |
