# Supplementary material for: The role of species ecology in predicting Toxoplasma gondii prevalence in wild and domesticated mammals globally
Source: PLoS Pathog. 2024 Jan 10;20(1):e1011908. doi: 10.1371/journal.ppat.1011908 (PMC10805296; doi:10.1371/journal.ppat.1011908)
Supplement: S3 Table — (DOCX) [file ppat.1011908.s003.docx]

**S3 Table.** Reference list of publications for which *T. gondii* prevalence data was available for free-ranging wild and domesticated mammal populations.

1. Abd El-Ghany A, Amin M. Epidemiology and molecular detection of zoonotic *Toxoplasma gondii* in cat feces and seroprevalence of anti-*Toxoplasma gondii* antibodies in pregnant women and sheep. *Life Science Journal* 2012; **9**: 133–146.
2. Abd El-Razik KA, Barakat AMA, Hussein HA, Younes AM, Elfadaly HA, Eldebaky HA *et al.* Seroprevalence, isolation, molecular detection and genetic diversity of *Toxoplasma gondii* from small ruminants in Egypt. *Journal of Parasitic Diseases* 2018; **42**: 527–536.
3. Abd-Rahman NH. Immunological Studies of Toxoplasmosis in The Sheep Model In Baghdad, Iraq. *International Journal of Advanced Biological Research* 2012; **2**: 778–781.
4. Abdallah M, Kamel M, Karima B, Samir A, Djamel K, Rachid K *et al.* Cross-Sectional Survey on *Toxoplasma gondii* Infection in Cattle, Sheep, and Goats in Algeria: Seroprevalence and Risk Factors. *Veterinary Sciences* 2019; **6**. doi:10.3390/vetsci6030063.
5. Abdel-Hafeez EH, Kamal AM, Abdelgelil NH, Abdel-Fatah M. Parasites Transmitted To Human By Ingestion of Different Types of Meat, El-Minia City, El-Minia Governorate, Egypt. *J Egypt Soc Parasitol* 2015; **45**: 671–680.
6. Abdelbaset A, Hamed M, Abushahba M, Rawy M, Sayed A, Adamovicz J. *Toxoplasma gondii* seropositivity and the associated risk factors in sheep and pregnant women in El-Minya Governorate, Egypt. *Veterinary World* 2020; **13**: 54–60.
7. Adesiyun, A., Knobel, D., Thompson, P., Wentzel, J., Kolo, F., Kolo, A., Conan, A., & Simpson, G. (2020). Sero-Epidemiological Study of Selected Zoonotic and Abortifacient Pathogens in Cattle at a Wildlife-Livestock Interface in South Africa. *Vector-Borne And Zoonotic Diseases*, *20*(4), 258–267
8. Afonso E, Thulliez P, Pontier D, Gilot-Fromont E. Toxoplasmosis in prey species and consequences for prevalence in feral cats: not all prey species are equal. *Parasitology* 2007; **134**: 1963–1971.
9. Afonso E, Germain E, Poulle ML, Ruette S, Devillard S, Say L *et al.* Environmental determinants of spatial and temporal variations in the transmission of *Toxoplasma gondii* in its definitive hosts. *International Journal for Parasitology: Parasites and Wildlife* 2013; **2**: 278–285.
10. Afonso E, Poulle ML, Lemoine M, Villena I, Aubert D, Gilot-Fromont E. Prevalence of *Toxoplasma gondii* in small mammals from the Ardennes region, France. *Folia Parasitologica* 2007; **54**: 313–314.
11. AFSSA. Toxoplasmose : état des connaissances et évaluation du risque lié à l’alimentation Rapport du groupe de travail *Toxoplasma gondii* de l’Afssa. 2005.
12. Aguirre AA, Keefe TJ, Reif JS, Kashinsky L, Yochem PK, Saliki JT *et al.* Infectious disease monitoring of the endangered Hawaiian monk seal. *Journal of Wildlife Diseases* 2007; **43**: 229–241.
13. Ahlers A, Mitchell MA, Dubey JP, Schooley RL, Heske EJ. Risk factors for *Toxoplasma gondii* exposure in semiaquatic mammals in a freshwater ecosystem. *Journal of Wildlife Diseases* 2015; **51**: 488–492.
14. Ahlers A, Wolf TM, Aarrestad O, Windels SK, Olson BT, Matykiewicz BR *et al.* Survey of *Toxoplasma gondii* exposure in muskrats in a relatively pristine ecosystem. *The Journal of Parasitology* 2020; **106**: 346–349.
15. Ahmad N, Iqbal Z, Mukhtar M, Mushtaq M, Khan K, Qayyum M. Seroprevalence and Associated Risk Factors of Toxoplasmosis in Sheep and Goats in Pothwar Region, Northern Punjab, Pakistan. *Pakistan Journal of Zoology* 2015; **47**: 161–167.
16. Ahmad N, Qayyum M. Seroprevalence and risk factors for toxoplasmosis in large ruminants in northern Punjab, Pakistan. *Journal of Infection in Developing Countries* 2014; **8**: 1022–1028.
17. Ahmed H, Malik A, Arshad M, Mustafa I, Khan M, Afzal M *et al.* Seroprevalence and Spatial Distribution of Toxoplasmosis in Sheep and Goats in North-Eastern Region of Pakistan. *Korean Journal of Parasitology* 2016; **54**: 439–446.
18. Akca A, Mor N. Seroprevalence of *Toxoplasma gondii* in Cattle in the Province of Kars, Turkey as Determined by ELISA. *Journal of Animal and Veterinary Advances* 2010; **9**: 876–878.
19. Åkerstedt J, Lillehaug A, Larsen IL, Eide NE, Arnemo JM, Handeland K. Serosurvey for Canine distemper virus, Canine adenovirus, *Leptospira interrogans*, and *Toxoplasma gondii* in free-ranging canids in Scandinavia and Svalbard. *Journal of Wildlife Diseases* 2010; **46**: 474–480.
20. Al Hamada A, Habib I, Barnes A, Robertson I. Risk factors associated with seropositivity to Toxoplasma among sheep and goats in Northern Iraq. *Veterinary Parasitology- Regional Studies and Reports* 2019; **15**. doi:10.1016/j.vprsr.2019.100264.
21. Al-Adhami BH, Simard M, Hernández-Ortiz A, Boireau C, Gajadhar AA. Development and evaluation of a modified agglutination test for diagnosis of Toxoplasma infection using tachyzoites cultivated in cell culture. *Food and Waterborne Parasitology* 2016; **2**: 15–21.
22. Al-Kappany Y, Abbas I, Devleesschauwer B, Dorny P, Jennes M, Cox E. Seroprevalence of anti-*Toxoplasma gondii* antibodies in Egyptian sheep and goats. *BMC Veterinary Research* 2018; **14**. doi:10.1186/s12917-018-1440-1.
23. Alanazi AD. Determination of seropositivity for *Toxoplasma gondii* in sheep, goats and camels slaughtered for food and human consumptions in Riyadh municipal abattoirs, Saudi Arabia. *Journal of the Egyptian Society of Parasitology* 2013; **43**: 569–76.
24. Alazemi MSH. Prevalence of anti-*Toxoplasma gondii* antibodies in aborted ewes in Kuwait. *Journal of the Egyptian Society of Parasitology* 2014; **44**: 393–6.
25. Alekseev AY, Reguzova AY, Rozanova EI, Abramov AV, Tumanov YV, Kuvshinova IN *et al.* Detection of specific antibodies to morbilliviruses, Brucella and Toxoplasma in the Black Sea dolphin *Tursiops truncatus ponticus* and the beluga whale *Delphinapterus leucas* from the Sea of Okhotsk in 2002-2007. *Russian Journal of Marine Biology* 2009; **35**: 494–497.
26. Alekseev AY, Rozanova EI, Ustinova EN, Tumanov YI, Kuvshinova IN, Shestopalov AM. The prevalence of antibodies to morbilliviruses, Brucella, and Toxoplasma in the Black Sea bottlenose dolphin *Tursiops truncatus ponticus* maintained in captivity. *Russian Journal of Marine Biology* 2007; **33**: 425–428.
27. Alekseev AY, Shpak OV, Adamenko LS, Glazov DM, Galkina IV, Schelkanov MY *et al.* Serological detection of causative agents of infectious and invasive diseases in the Beluga Whale Delphinapterus leucas (Pallas, 1776) (Cetacea: Monodontidae) from Sakhalinsky Bay. *Russian Journal of Marine Biology* 2017; **43**: 485–490.
28. Almeida JC, Melo RPB, Kim PCP, Guerra NR, Alves LC, Costa DF *et al.* Molecular and serological investigation of infectious diseases in captive and free-range crab-eating fox (Cerdocyon thous - Linnaeus, 1776) from northeastern Brazil. *Acta Parasitologica* 2018; **63**: 184–189.
29. Almería S, Cabezón O, Paniagua J, Cano-Terriza D, Jiménez-Ruiz S, Arenas-Montes A *et al.* *Toxoplasma gondii* in sympatric domestic and wild ungulates in the Mediterranean ecosystem. *Parasitology Research* 2018; **117**: 665–671.
30. Almería S, Calvete C, Pagés A, Gauss C, Dubey JP. Factors affecting the seroprevalence of *Toxoplasma gondii* infection in wild rabbits (*Oryctolagus cuniculus*) from Spain. *Veterinary Parasitology* 2004; **123**: 265–270.
31. Almería S, Vidal D, Ferrer D, Pabón M, Fernández-de-Mera MIG, Ruiz-Fons F *et al.* Seroprevalence of *Neospora caninum* in non-carnivorous wildlife from Spain. *Veterinary Parasitology* 2007; **143**: 21–28.
32. Almería S, Cano-Terriza D, Prieto P, Dubey JP, Jiménez-Martín D, Castro-Scholten S *et al.* Seroprevalence and risk factors of *Toxoplasma gondii* infection in wild ungulates that cohabit in a natural park with human–animal interaction in the Mediterranean ecosystem. *Zoonoses and Public Health* 2021; **68**: 263–270.
33. Alvarado-Esquivel C, Estrada-Malacón M, Reyes-Hernández S, Pérez-Ramírez J, Trujillo-López J, Villena I *et al.* High Prevalence of *Toxoplasma gondii* Antibodies in Domestic Pigs in Oaxaca State, Mexico. *Journal of Parasitology* 2012; **98**: 1248–1250.
34. Alvarado-Esquivel C, Estrada-Malacón M, Reyes-Hernández S, Pérez-Ramírez J, Trujillo-López J, Villena I *et al.* Seroprevalence of *Toxoplasma gondii* in Domestic Sheep in Oaxaca State, Mexico. *Journal of Parasitology* 2013; **99**: 151–152.
35. Alvarado-Esquivel C, García-Machado C, Alvarado-Esquivel D, González-Salazar A, Briones-Fraire C, Vitela-Corrales J *et al.* Seroprevalence of *Toxoplasma gondii* Infection In Domestic Pigs In Durango State, Mexico. *Journal of Parasitology* 2011; **97**: 616–619.
36. Alvarado-Esquivel C, Garcia-Machado C, Alvarado-Esquivel D, Vitela-Corrales J, Villena I, Dubey J. Seroprevalence of *Toxoplasma gondii* Infection In Domestic Sheep In Durango State, Mexico. *Journal of Parasitology* 2012; **98**: 271–273.
37. Alvarado-Esquivel C, Romero-Salas D, García-Vázquez Z, Crivelli-Diaz M, Barrientos-Morales M, Lopez-de-Buen L *et al.* Seroprevalence and correlates of *Toxoplasma gondii* infection in domestic pigs in Veracruz State, Mexico. *Tropical Animal Health and Production* 2014; **46**: 705–709.
38. Alvarado-Esquivel C, Silva-Aguilar D, Villena I, Dubey J. Seroprevalence and correlates of *Toxoplasma gondii* infection in domestic sheep in Michoacan State, Mexico. *Preventive Veterinary Medicine* 2013; **112**: 433–437.
39. Alvarado-Esquivel C, Vazquez-Morales RF, Colado-Romero EE, Guzman-Sanchez R, Liesenfeld O, Dubey JP. Prevalence of infection with *Toxoplasma gondii* in landrace and mixed breed pigs slaughtered in Baja California Sur State, Mexico. *European journal of microbiology & immunology* 2015; **5**: 112–5.
40. Ammar S, Braunstein J, Su C, Williamson RH, Gerhold R. Serologic Survey of *Toxoplasma gondii* in Black Bears (*Ursus americanus*) from Eastern Tennessee, USA. *Journal of Wildlife Diseases* 2020; **56**: 721–723.
41. Andrade M, Carneiro M, Medeiros A, Neto V, Vitor R. Seroprevalence and Risk Factors associated with ovine toxoplasmosis in Northeast Brazil. *Parasite* 2013; **20**. doi:10.1051/parasite/2013019.
42. Antolova D, Reiterova K, Dubinsky P. Seroprevalence of *Toxoplasma gondii*  in wild boars (*Sus scrofa*) in the Slovak Republic. *Annals of Agricultural and Environmental Medicine* 2007; **14**.
43. Aramini JJ, Stephen C, Dubey JP. *Toxoplasma gondii* in Vancouver Island Cougars (*Felis concolor vancouverensis*): Serology and oocyst shedding. *The Journal of Parasitology* 1998; **84**: 438.
44. Aramini JJ, Stephen C, Dubey JP, Engelstoft C, Schwantje H, Ribble CS. Potential contamination of drinking water with *Toxoplasma gondii* oocysts. *Epidemiology and Infection* 1999; **122**: 305–315.
45. Arias ML, Reyes L, Chinchilla M, Linde E. Seroepidemiology of *Toxoplasma gondii* (Apicomplexa) in meat producing animals in Costa Rica. *Revista de Biología Tropical* 1994; **42**: 15–20.
46. Arko-Mensah J, Bosompem KM, Canacoo EA, Wastling JM, Akanmori BD. The Seroprevalence Of Toxoplasmosis in Pigs In Ghana. *Acta Tropica* 2000; **76**: 27–31.
47. Armand B, Solhjoo K, Shabani-Kordshooli M, Davami MH, Sadeghi M. Toxoplasma infection in sheep from south of Iran monitored by serological and molecular methods; risk assessment to meat consumers. *Veterinary world* 2016; **9**: 850–5.
48. Asgari Q, Mehrabani D, Moazzeni M, Akrami-Mohajeri F, Kalantari M, Motazedian M *et al.* The Seroprevalence of Ovine Toxoplasmosis in Fars Province, Southern Iran. *Asian Journal of Animal and Veterinary Advances* 2009; **4**: 332–336.
49. Asgari Q, Sarkari B, Amerinia M, Panahi S, Mohammad-pour I, Sarvestani A. Toxoplasma Infection in Farm Animals: A Seroepidemiological Survey in Fars Province, South of Iran. *Jundishapur Journal of Microbiology* 2013; **6**: 269–272.
50. Asgari Q, Mehrabani D, Moazeni M, Mohajeri FA, Kalantari M, Motazedian MH *et al.* The seroprevalence of bovine toxoplasmosis in Fars Province, Southern Iran. *Asian Journal of Animal and Veterinary Advances* 2010; **5**: 210–216.
51. Assadi-Rad AM, New JC, Patton S. Risk factors associated with transmission of *Toxoplasma gondii* to sows kept in different management systems in Tennessee. *Veterinary Parasitology* 1995; **57**: 289–297.
52. Aston EJ, Mayor P, Bowman DD, Mohammed HO, Liotta JL, Kwok O *et al.* Use of filter papers to determine seroprevalence of *Toxoplasma gondii* among hunted ungulates in remote Peruvian Amazon. *International Journal for Parasitology: Parasites and Wildlife* 2014; **3**: 15–19.
53. Astorga R, Reguillo L, Hernández M, Cardoso-Toset F, Tarradas C, Maldonado A *et al.* Serosurvey on Schmallenberg Virus and Selected Ovine Reproductive Pathogens in Culled Ewes From Southern Spain. *Transboundary and Emerging Diseases* 2014; **61**: 4–11.
54. Aubert D, Ajzenberg D, Richomme C, Gilot-Fromont E, Terrier ME, Gevigney C de *et al.* Molecular and biological characteristics of *Toxoplasma gondii* isolates from wildlife in France. *Veterinary Parasitology* 2010; **171**: 346–349.
55. Ayinmode A, Akinseye V, Schares G, Cadmus S. Serological Survey of Toxoplasmosis, Neosporosis and Brucellosis among Cattle Herds in Oyo State, South-Western Nigeria. *African Journal of Infectious Diseases* 2017; **11**: 95–101.
56. Azevedo SS de, Pena HF de J, Alves CJ, Guimarães Filho AA de M, Oliveira RM, Maksimov P *et al.* Prevalence of anti-*Toxoplasma gondii* and anti-*Neospora caninum* antibodies in swine from Northeastern Brazil. *Rev Bras Parasitol Vet* 2010; **19**: 80–84.
57. Bacci C, Vismarra A, Mangia C, Bonardi S, Bruini I, Genchi M *et al.* Detection of *Toxoplasma gondii* in free-range, organic pigs in Italy using serological and molecular methods. *International Journal of Food Microbiology* 2015; **202**: 54–56.
58. Bachand N, Ravel A, Leighton P, Stephen C, Ndao M, Avard E *et al.* Serological and molecular detection of *Toxoplasma gondii* in terrestrial and marine wildlife harvested for food in Nunavik, Canada. *Parasites & Vectors* 2019; **12**: 155.
59. Balea A, Paştiu AI, Györke A, Mircean V, Cozma V. The dynamics of anti-*Toxoplasma gondii* antibodies (IgG) in small ruminants and pigs from Cluj County, Romania. 2012.
60. Balkaya İ, Ütük A, Babur Ç, Beyhan Y, Pıskı F, Sözdutmaz İ. Detection of *Toxoplasma gondii an*d *Neospora caninum* antibodies in Wild Boars (*Sus scrofa*) in Eastern Turkey. *Israel Journal of Veterinary Medicine* 2015; **70**.
61. Bamba S, Halos L, Tarnagda Z, Alanio A, Macé P, Moukoury S *et al.* Seroprevalence of *Toxoplasma gondii* and direct genotyping using mini-sequencing in free-range pigs in Burkina Faso. *International Journal of Food Microbiology* 2016; **230**: 10–15.
62. Bamba S, Bazié WW, Lingani M, Sourabié Y, Ndiaye JL, Tarnagda Z. Seroepidemiology of *Toxoplasma gondii* infection among slaughtered pigs, cattle and goats for human consumption in Bobo-Dioulasso, Burkina Faso. *Journal of Parasitology and Vector Biology* 2017; **9**: 106–110.
63. Barakat AMA, Elaziz MMA, Fadaly HA. Comparative diagnosis of toxoplasmosis in Egyptian small ruminants by indirect hemagglutination assay and Elisa. *Global Veterinaria* 2009; **3**: 9–14.
64. Barbosa W de O, Coelho TG, Costa TO da, Paiz LM, Fornazari F, Langoni H *et al.* Antibodies to *Toxoplasma gondii*, Leishmania spp., and *Leptospira* spp. in Free-Ranging Six-Banded Armadillos (Euphractus sexcinctus) from Northeastern Brazil. *Journal of Wildlife Diseases* 2020; **56**: 486.
65. Bard SM, Cain JW. Pathogen prevalence in American black bears (*Ursus americanus amblyceps)* of the Jemez Mountains, New Mexico, USA. *Journal of Wildlife Diseases* 2019; **55**: 745–754.
66. Barros M, Cabezón O, Dubey JP, Almería S, Ribas MP, Escobar LE *et al.* *Toxoplasma gondii* infection in wild mustelids and cats across an urban-rural gradient. *PLOS ONE* 2018; **13**: 1–17.
67. Barroso P, García-Bocanegra I, Acevedo P, Palencia P, Carro F, Jiménez-Ruiz S *et al.* Long-Term Determinants of the Seroprevalence of *Toxoplasma gondii* in a Wild Ungulate Community. *Animals* 2020; **10**: 2349.
68. Bártová E, Kobédová K, Lamka J, Kotrba R, Vodicka R, Sedlák K. Seroprevalence of *Neospora caninum* and *Toxoplasma gondii* in exotic ruminants and camelids in the Czech Republic. *Parasitology Research* 2017; **116**: 1925–1929.
69. Bártová E, Sedlák K, Literák I. *Toxoplasma gondii* and *Neospora caninum* antibodies in sheep in the Czech Republic. *Veterinary Parasitology* 2009; **161**: 131–132.
70. Bártová E, Sedlák K. Seroprevalence of *Toxoplasma gondii* and *Neospora caninum* in slaughtered pigs in the Czech Republic. *Parasitology* 2011; **138**: 1369–1371.
71. Bártová E, Sedlák K, Budikova M. A study of *Toxoplasma gondii* and *Neospora caninum* antibody seroprevalence in healthy cattle in the Czech Republic. *Annals of Agricultural and Environmental Medicine* 2015; **22**. doi:10.5604/12321966.1141365.
72. Bártová E, Sedlák K, Literák I. Prevalence of *Toxoplasma gondii* and *Neospora caninum* antibodies in wild boars in the Czech Republic. *Veterinary Parasitology* 2006; **142**: 150–153.
73. Bártová E, Slezakova R, Nagl I, Sedlak K. *Neospora caninum* and *Toxoplasma gondii* antibodies in red foxes (*Vulpes vulpes*) in the Czech Republic. *Annals of Agricultural and Environmental Medicine* 2016; **23**. doi:10.5604/12321966.1196858.
74. Bártová E, Sedlák K, Treml F, Holko I, Literák I. *Neospora caninum* and *Toxoplasma gondii* antibodies in European brown hares in the Czech Republic, Slovakia and Austria. *Veterinary Parasitology* 2010; **171**: 155–158.
75. Basso W, Hartnack S, Pardini L, Maksimov P, Koudela B, Venturini M *et al.* Assessment of diagnostic accuracy of a commercial ELISA for the detection of *Toxoplasma gondii* infection in pigs compared with IFAT, TgSAG1-ELISA and Western blot, using a Bayesian latent class approach. *International Journal for Parasitology* 2013; **43**: 565–570.
76. Bena M, Vasickova P, Kamler J, Kubankova M. Prevalence of selected emerging and re-emerging pathogens in population of wild boar in the Czech Republic. In: *12th International Symposium on Wild Boar and Other Suids*. Lázně Bělohrad, Czech Republic, 2019, p 67.
77. Benkirane A, Essamkaoui S, El Idrissi A, Lucchese L, Natale A. A sero-survey of major infectious causes of abortion in small ruminants in Morocco. *Veterinaria Italiana* 2015; **51**: 25–30.
78. Benlakehal A, Miroud K, Djeghim H, Kaidi R. Serological survey for anti-*Toxoplasma gondii* antibodies in sheep of northeastern Algeria. *Tropical Animal Health and Production* 2019; **51**: 2227–2233.
79. Bentum KE, Folitse RD, Amemor E, Burimuah V, Opoku-Agyemang T, Emikpe BO. Seroprevalence of *Toxoplasma gondii* antibodies in sheep and goats slaughtered at the Kumasi Abattoir, Ghana. *Journal of Immunoassay & Immunochemistry* 2019; **40**: 495–501.
80. Beral M, Rossi S, Aubert D, Gasqui P, Terrier ME, Klein F *et al.* Environmental factors associated with the seroprevalence of *Toxoplasma gondii* in wild boars (*Sus scrofa*), France. *EcoHealth* 2012; **9**: 303–309.
81. Berger-Schoch AE, Bernet D, Doherr MG, Gottstein B, Frey CF. *Toxoplasma gondii* in Switzerland: a serosurvey based on meat juice analysis of slaughtered pigs, wild boar, sheep and cattle. *Zoonoses Public Health* 2011; **58**: 472–478.
82. Berger-Schoch A, Herrmann D, Schares G, Müller N, Bernet D, Gottstein B *et al.* Prevalence and genotypes of *Toxoplasma gondii* in feline faeces (oocysts) and meat from sheep, cattle and pigs in Switzerland. *Veterinary Parasitology* 2011; **177**: 290–297.
83. Bevins SN, Carver S, Boydston EE, Lyren LM, Alldredge M, Logan KA *et al.* Three Pathogens in Sympatric Populations of Pumas, Bobcats, and Domestic Cats: Implications for Infectious Disease Transmission. *PLoS ONE* 2012; **7**: e31403.
84. Bezerra M, Cruz J, Kung E, de Melo R, Gomes A, de Moraes E *et al.* Detection of *Toxoplasma gondii* in the reproductive organs of rams in Brazil. *PESQUISA Veterinaria Brasileira* 2013; **33**: 989–991.
85. Bezerra M, Cruz J, Kung E, Silva J, Santos A, Moraes É *et al.* Occurrence of *Toxoplasma gondii* DNA in sheep naturally infected and slaughtered in abattoirs in Pernambuco, Brazil. *Pesquisa Veterinaria Brasileira* 2014; **34**: 329–331.
86. Bezerra R, Carvalho F, Guimaraes L, Rocha D, Maciel B, Wenceslau A *et al.* Genetic characterization of *Toxoplasma gondii* isolates from pigs intended for human consumption in Brazil. *Veterinary Parasitology* 2012; **189**: 153–161.
87. Bezerra RA, Paranhos EB, Del’Arco AE, Albuquerque GR. Detection anti-*Toxoplasma gondii* antibodies in swines bred and abated in the Bahia State, Brazil. *Rev Bras Parasitol Vet* 2009; **18**: 78–80.
88. Biazus AH, Dezen D, Barros LD de, Garcia JL, Portella LP, Vogel FF *et al.* Antibodies against *Toxoplasma gondii* in bats (*Desmodus rotundus*) captured near caves in cities from western region of Santa Catarina State, Brazil. *Comparative Clinical Pathology* 2016; **25**: 505–507.
89. Binninger CE, Beecham JJ, Thomas LA, Winward LD. A serologic survey for selected infectious diseases of black bears in Idaho. *Journal of Wildlife Diseases* 1980; **16**: 423–430.
90. Bisias G, Burriel AR, Boutsini S, Kritas SK, Leontides LS. A serological investigation of some abortion causes among small ruminant flocks in Greece. *Internet Journal of Veterinary Medicine* 2010; **8**.
91. Bispo MDS, Faustino MADG, Alves LC, Salcedo JHP, Souza CHD, Sousa DPD *et al.* Frequência De Anticorpos Anti- *Toxoplasma gondii* Em Propriedades De Criação De Caprinos E Ovinos No Estado De Pernambuco. *Ci Anim Bras* 2011; **12**: 291–297.
92. Blaga R, Aubert D, Thébault A, Perret C, Geers R, Thomas M *et al.* *Toxoplasma gondii* in beef consumed in France: regional variation in seroprevalence and parasite isolation. *Parasite* 2019; **26**. doi:10.1051/parasite/2019076.
93. Blanchet MA, Godfroid J, Breines EM, Heide-Jørgensen MP, Nielsen NH, Hasselmeier I *et al.* West Greenland harbour porpoises assayed for antibodies against *Toxoplasma gondii*: False positives with the direct agglutination method. *Diseases of Aquatic Organisms* 2014; **108**: 181–186.
94. Bocharova N, Treu G, Czirják GÁ, Krone O, Stefanski V, Wibbelt G *et al.* Correlates between Feeding Ecology and Mercury Levels in Historical and Modern Arctic Foxes (*Vulpes lagopus*). *PLoS ONE* 2013; **8**: 1–11.
95. Bollo E, Pregel P, Gennero S, Pizzoni E, Rosati S, Nebbia P *et al.* Health status of a population of nutria (*Myocastor coypus*) living in a protected area in Italy. *Research in Veterinary Science* 2003; **75**: 21–25.
96. Bondo KJ, Macbeth B, Schwantje H, Orsel K, Culling D, Culling B *et al.* Health Survey of Boreal Caribou (*Rangifer tarandus caribou*) in Northeastern British Columbia, Canada. *Journal of Wildlife Diseases* 2019; **55**: 544.
97. Bosio FS, Zanet MS, Gennero S, Bergagna C, Canton C, Trisciuoglio A *et al.* *Toxoplasma gondii* in Piedmont: Epidemiology in wildlife and livestock. In: *LXVII Convegno Nazionale, Centro Congressi Camera di Commercio*. Brescia, Italy, 2013, pp 17–19.
98. Bossart GD, Mignucci-Giannoni AA, Rivera-Guzman AL, Jimenez-Marrero NM, Camus AC, Bonde RK *et al.* Disseminated toxoplasmosis in Antillean manatees *Trichechus manatus manatus* from Puerto Rico. *Diseases of Aquatic Organisms* 2012; **101**: 139–144.
99. Bouchard É, Elmore SA, Alisauskas RT, Samelius G, Gajadhar AA, Schmidt K *et al.* Transmission dynamics of *Toxoplasma gondii* in arctic foxes (*Vulpes Lagopus*): A long-term mark-recapture serologic study at Karrak Lake, Nunavut, Canada. *Journal of Wildlife Diseases* 2019; **55**: 619–626.
100. Boughattas S, Ayari K, Sa T, Aoun K, Bouratbine A. Survey of the Parasite *Toxoplasma gondii* in Human Consumed Ovine Meat in Tunis City. *PLOS ONE* 2014; **9**. doi:10.1371/journal.pone.0085044.
101. Brandão LNS, Rosa JMA, Kramer B, Sousa ATHI de, Trevisol IM, Silva VS *et al.* Detection of *Toxoplasma gondii* infection in feral wild boars (*Sus scrofa*) through indirect hemagglutination and PCR. *Ciencia Rural* 2019; **49**. doi:10.1590/0103-8478cr20180640.
102. Brandell EE, Cross PC, Craft ME, Smith DW, Dubovi EJ, Gilbertson MLJ *et al.* Patterns and processes of pathogen exposure in gray wolves across North America. *Sci Rep* 2021; **11**: 3722.
103. Brillhart DB, Fox LB, Dubey JP, Upton SJ. Seroprevalence of *Toxoplasma gondii* in wild mammals in Kansas. *Journal of the Helminthological Society of Washington* 1994; **61**: 117–121.
104. Briscoe N, Humphreys JG, Dubey JP. Prevalence of *Toxoplasma gondii i*nfections in Pennsylvania black bears, *Ursus americanus*. *Journal of Wildlife Diseases* 1993; **29**: 599–601.
105. Bronson E, Spiker H, Driscoll CP. Serosurvey for selected pathogens in free-ranging American black bears (*Ursus americanus*) in Maryland, USA. *Journal of Wildlife Diseases* 2014; **50**: 829–836.
106. Brouat C, Diagne CA, Ismaïl K, Aroussi A, Dalecky A, Bâ K *et al.* Seroprevalence of *Toxoplasma gondii* in commensal rodents sampled across Senegal, West Africa. *Parasite* 2018; **25**: 32.
107. Brown M, Lappin MR, Brown JL, Munkhtsog B, Swanson WF. Exploring the ecologic basis for extreme susceptibility of Pallas’ cats (*Otocolobus manul*) to fatal toxoplasmosis. *Journal of Wildlife Diseases* 2005; **41**: 691–700.
108. Bueno MG, Catão-Dias JL, Laroque P de O, Vasconcellos SA, Neto JSF, Gennari SM *et al.* Infectious Diseases in Free-Ranging Blonde Capuchins, *Sapajus flavius,* in Brazil. *International Journal of Primatology* 2017; **38**: 1017–1031.
109. Burrells A, Bartley PM, Zimmer IA, Roy S, Kitchener AC, Meredith A *et al.* Evidence of the three main clonal *Toxoplasma gondii* lineages from wild mammalian carnivores in the UK. *Parasitology* 2013; **140**: 1768–1776.
110. Burridge MJ, Bigler WJ, Forrester DJ, Hennemann JM. Serologic survey for *Toxoplasma gondii* in wild animals in Florida. *Journal of the American Veterinary Medical Association* 1979; **175**: 964–967.
111. Buxton D, Maley SW, Pastoret PP, Brochier B, Innes EA. Examination of red foxes (*Vulpes vulpes*) from Belgium for antibody to *Neospora caninum* and *Toxoplasma gondii*. *Veterinary Record* 1997; **141**: 308–309.
112. Cabezón O, Hall AJ, Vincent C, Pabón M, García-Bocanegra I, Dubey JP *et al.* Seroprevalence of *Toxoplasma gondii* in North-eastern Atlantic harbor seal (*Phoca vitulina vitulina*) and grey seal (*Halichoerus grypus*). *Veterinary Parasitology* 2011; **179**: 253–256.
113. Cabezón O, Resendes AR, Domingo M, Raga JA, Agustí C, Alegre F *et al.* Seroprevalence of *Toxoplasma gondii* antibodies in wild dolphins from the Spanish Mediterranean Coast. *The Journal of Parasitology* 2004; **90**: 643–644.
114. Cabral AD, D’Auria SRN, Camargo MCGO, Rosa AR, Sodré MM, Galvão-Dias MA *et al.* Seroepidemiology of *Toxoplasma gondii* infection in bats from São Paulo city, Brazil. *Veterinary Parasitology* 2014; **206**: 293–296.
115. Cabral AD, Su C, Soares RM, Gennari SM, Sperança MA, da Rosa AR *et al.* Occurrence and diversity of Sarcocystidae protozoa in muscle and brain tissues of bats from São Paulo state, Brazil. *International Journal for Parasitology: Parasites and Wildlife* 2021; **14**: 91–96.
116. Cademartori BG, Santos LMJF, Oliveira FC, Quevedo P, Oliveira PA, Ramos TS *et al.* Isolation and pathogenicity of *Toxoplasma gondii* in naturally infected (rustic farm) pigs in southern Brazil. *Veterinary Parasitology* 2014; **203**: 207–211.
117. Cai Q, Ma L. Establishment and serological and serological investigation of ELISA method for the diagnosis of toxoplasmosis in sheep in Qinghai Province. *Chin J Vet Med* 2010; **46**: 39–40.
118. Cai Z, Li F, Hu P, Guan W, Li C, Cao S. Seroprevalence and analysis of *Toxoplasma gondii* infection in partial pig farms in suburban areas of Beijing. *Heilongjiang Anim Sci Vet Med* 2017; **8**: 103–104.
119. Caiwang C, Li X, Wang G, Ma L. Serological detection of abortion disease among sheep in Maqin County of Qinghai Province. *Qinghai J Anim Vet Sci* 2015; **45**: 6–7.
120. Calero-Bernal R, Pérez-Martín JE, Reina D, Serrano FJ, Frontera E, Fuentes I *et al.* Detection of Zoonotic Protozoa *Toxoplasma gondii* and *Sarcocystis suihominis* in Wild Boars from Spain. *Zoonoses and Public Health* 2016; **63**: 346–350.
121. Calvo-Mac C, Gutleb A, Contal S, Ilukewitsch V, Muñoz-Zanzi C, Medina-Vogel G. Exposure to *Toxoplasma gondii* in Marine Otters ( *Lontra felina* ) and Domestic Cats (*Felis catus)* in an Arid Environment in Chile. *Journal of Wildlife Diseases* 2020. doi:10.7589/2019-10-269.
122. Camossi L, Greca H, Corrêa A, Richini-Pereira V, Silva R, Da Silva A *et al.* Detection of *Toxoplasma gondii* DNA in the milk of naturally infected ewes. *Veterinary Parasitology* 2011; **177**: 256–261.
123. Campero LM, Schott F, Gottstein B, Deplazes P, Sidler X, Basso W. Detection of antibodies to *Toxoplasma gondii* in oral fluid from pigs. *International Journal for Parasitology* 2020; **50**: 349–355.
124. Cañon-Franco AWA, Yai LEO, Joppert AM, Souza CE, Auria SRND, Dubey P *et al.* Seroprevalence of *Toxoplasma gondii* antibodies in the rodent Capybara (*Hidrochoeris hidrochoeris*) from Brazil. *Journal of Parasitology* 2003; **89**: 4–5.
125. Cañón-Franco WA, Araújo FAP, López-Orozco N, Jardim MMA, Keid LB, Dalla-Rosa C *et al.* *Toxoplasma gondii* in free-ranging wild small felids from Brazil: molecular detection and genotypic characterization. *Veterinary parasitology* 2013; **197**: 462–469.
126. Caňón-Franco WA, de Araújo FAP, Gennari SM. *Toxoplasma gondii* in small neotropical wild felids. *Brazilian Journal of Veterinary Research and Animal Science* 2013; **50**: 50–67.
127. Cao W, Liu T, Zhang M, Wang M, Zhang Y, Zhao Y *et al.* Epidemiological survey of serum *Toxoplasma gondii* in some areas of Hebei Province. *The 13th academic seminar of veterinary parasitology of Chinese Association of Animal Science and Veterinary Medicine, Harbin* 2015:54.
128. Caporali EHG. De Anticorpos Anti-*Toxoplasma gondii* Em Suínos Dos Estados De. *Arq ciên vet zool UNIPAR* 2005; **8**: 19–24.
129. Carletti R, Freire R, Navarro I. Prevalence of *Toxoplasma gondii* infection among slaughtered swine in Parana State, Brazil. *Semina - Ciencias Agaria* 2005; **26**: 563–568.
130. Carlson-Bremer D, Colegrove KM, Gulland FMD, Conrad PA, Mazet JAK, Johnson CK. Epidemiology and pathology of *Toxoplasma gondii* in free-ranging California sea lions (*Zalophus californianus*). *Journal of Wildlife Diseases* 2015; **51**: 362–373.
131. Carlsson AM, Curry P, Elkin B, Russell D, Veitch A, Branigan M *et al.* Multi-pathogen serological survey of migratory caribou herds: A snapshot in time. *Plos One* 2019; **14**: e0219838.
132. Carme B, Aznar C, Motard A, Demar M, Thoisy B de. Serologic survey of *Toxoplasma gondii* in noncarnivorous free-ranging neotropical mammals in French Guiana. *Vector borne and zoonotic diseases* 2002; **2**: 11–17.
133. Castillo-Cuenca J, Díaz-Gao J, Martínez-Moreno A, Cano-Terriza D, Jiménez-Ruiz S, Almería S *et al.* Seroepidemiology of *Toxoplasma gondii* in extensively raised Iberian pigs in Spain. *Preventative Veterinary Medicine* 2020; **175**. doi:10.1016/j.prevetmed.2019.104854.
134. Castro-Scholten S, Cano-Terriza D, Jiménez-Ruiz S, Almería S, Risalde MA, Vicente J *et al.* Seroepidemiology of *Toxoplasma gondii* in wild ruminants in Spain. *Zoonoses and Public Health* 2021; **68**: 884–895.
135. Cavalcante GT, Aguiar DM, Chiebao D, Dubey JP, Ruiz VLA, Dias RA *et al.* Seroprevalence of *Toxoplasma gondii* Antibodies in Cats and Pigs From Rural Western Amazon, Brazil. *Journal of Parasitology* 2006; **92**: 863–864.
136. Cenci-Goga B, Ciampelli A, Sechi P, Veronesi F, Moretta I, Cambiotti V *et al.* Seroprevalence and risk factors for *Toxoplasma gondii* in sheep in Grosseto district, Tuscany, Italy. *BMC Veterinary Research* 2013; **9**. doi:10.1186/1746-6148-9-25.
137. Chadwick EA, Cable J, Chinchen A, Francis J, Guy E, Kean EF *et al.* Seroprevalence of *Toxoplasma gondii* in the Eurasian otter (*Lutra lutra*) in England and Wales. *Parasites & Vectors* 2013; **6**: 75.
138. Chalupský J, Vávra J, Gaudin JC, Vandewalle P, Arthur CP, Guenezan M *et al.* Serological evidence of the occurrence of encephalitozoonosis and toxoplasmosis in the European wild rabbit (*Oryctolagus cuniculus*) in France. *Bulletin de la Société Française de Parasitologie* 1990; **8**: 91–95.
139. Chandrawathani P, Nurulaini R, Zanin CM, Premaalatha B, Adnan M, Jamnah O *et al.* Seroprevalence of *Toxoplasma gondii* antibodies in pigs, goats, cattle, dogs and cats in peninsular Malaysia. *Trop Biomed* 2008; **25**: 257–258.
140. Chang GN, Tsai SS, Kuo M, Dubey JP. Epidemiology of swine toxoplasmosis in Taiwan. *Southeast Asian J Trop Med Public Health* 1991; **22 Suppl**: 111–114.
141. Chang Q, Zheng X, Qiu J, Wang C, Zhu X. Seroprevalence of *Toxoplasma gondii* Infection in Fattening Pigs in Northeast China. *Journal of Parasitology* 2013; **99**: 544–545.
142. Cheadle MA, Spencer JA, Blagburn BL. Seroprevalences of *Neospora caninum* and *Toxoplasma gondii* in nondomestic felids from southern Africa. *Journal of Zoo and Wildlife Medicine* 1999; **30**: 248–251.
143. Chen C, Fan X, Tan M, Liu Q. Serological survey and analysis on Brucella, Chlamydia, and Toxoplasmosis on scale dairy farming in Huangyuan county of Qinghai. *Chinese Qinghai Journal of Animal and Veterinary Sciences* 2017; **47**: 33–35.
144. Chen K, Huang S-Y, Wang J-L, Hu R-L, Yao Q-X, Zhang S-F *et al.* Prevalence and genetic characterization of *Toxoplasma gondii* in badgers (*Melogale moschata*) in southern China by PCR-RFLP. *Infection, Genetics and Evolution* 2017; **52**: 30–33.
145. Chen L. Serological investigation of toxoplasmosis in dairy cattle of Jiyuan City. *The Chinese Livestock and Poultry Breeding* 2018; **14**: 8–9.
146. Chen Y, Huangpu H, Shi D, Song Y, Wang Z. Serological investigation of *Toxoplasma gondii* in cows in some areas of Henan Province. *Chinese Journal of Veterinary Medicine* 2017; **53**: 45–46.
147. Cheng T, Halper B, Siebert J, Cruz-Martinez L, Chapwanya A, Kelly P *et al.* Parasites of small Indian mongoose, *Herpestes auropunctatus*, on St. Kitts, West Indies. *Parasitology Research* 2018; **117**: 989–994.
148. Chikweto A, Kumthekar S, Tiwari K, Nyack B, Deokar MS, Stratton G *et al.* Seroprevalence of *Toxoplasma gondii* in Pigs, Sheep, Goats, and Cattle from Grenada and Carriacou, West Indies. *Journal of Parasitology* 2011; **97**: 950–951.
149. Chițimia L, Nalbaru AM, Banu T, Apostu C, Constantinoiu C. Seroprevalence of ovine toxoplasmosis by indirect immunofluorescence test. *Revista Română de Medicină Veterinară* 2011; **21**: 136–141.
150. Chomel ABB, Zarnke RL, Kasten RW, Kass PH, Chomel BB, Mendes E *et al.* Serologic survey of *Toxoplasma gondii* in grizzly bears (*Ursus arctos)* and black bears (*Ursus americanus*) from Alaska, 1988 to 1991. *Journal of Wildlife Diseases* 1995; **31**: 472–479.
151. Chomel BB, Carniciu ML, Kasten RW, Castelli PM, Work TM, Jessup DA. Antibody prevalence of eight ruminant infectious diseases in California mule and black-tailed deer (*Odocoileus hemionus*). *Journal of Wildlife Diseases* 1994; **30**: 51–59.
152. Choudhary S, Zieger U, Sharma RN, Chikweto A, Tiwari KP, Ferreira LR *et al.* Isolation and RFLP genotyping of *Toxoplasma gondii* from the Mongoose (*Herpestes auropunctatus*) in Grenada, West Indies. *Journal of Zoo and Wildlife Medicine* 2013; **44**: 1127–1130.
153. Çiçek H, Babür C, Eser M. Seroprevalence of *Toxoplasma gondii* in Pırlak sheep in the Afyonkarahisar Province of Turkey. *Turkiye Parazitol Derg* 2011; **35**: 137–139.
154. Clark RK, Boyce WM, Jessup DA, Elliott LF. Survey of Pathogen Exposure among Population Clusters of Bighorn Sheep ( Ovis canadensis ) in California. *Journal of Zoo and Wildlife Medicine* 1993; **24**: 48–53.
155. Clementino Andrade MM, Pinheiro BV, Cunha MM, Carneiro ACAV, andrade Neto VF, Vitor RWA. New gentotypes of *Toxoplasma gondii* obtained from farm animals in Northeast Brazil. *Research in Veterinary Science* 2013; **94**: 587–589.
156. Clifford DL, Mazet JAK, Dubovi EJ, Garcelon DK, Coonan TJ, Conrad PA *et al.* Pathogen exposure in endangered island fox (*Urocyon littoralis*) populations: Implications for conservation management. *Biological Conservation* 2006; **131**: 230–243.
157. Closa-Sebastià F, Casas-Díaz E, Cuenca R, Lavín S, Mentaberre G, Marco I. Antibodies to selected pathogens in wild boar (Sus scrofa) from Catalonia (NE Spain). *European Journal of Wildlife Research* 2011; **57**: 977–981.
158. Coelho C, Vieira-Pinto M, Faria A, Vale-Gonçalves H, Veloso O, Paiva-Cardoso M *et al.* Serological evidence of *Toxoplasma gondii* in hunted wild boar from Portugal. *Veterinary Parasitology* 2014; **202**: 310–312.
159. Coelho C, Vieira-Pinto M, Vilares A, Gargaté MJ, Rodrigues M, Cardoso L *et al.* PCR Detection of *Toxoplasma gondii* in European Wild Rabbit (Oryctolagus cuniculus) from Portugal. *Microorganisms* 2020; **8**: 1926.
160. Cole ARA, Lindsay DS, Howe DK, Roderick CL, Dubey JP, Thomas NJ *et al.* Biological and molecular characterizations of *Toxoplasma gondii* strains obtained from Southern Sea Otters (*Enhydra lutris nereis*). *Journal of Parasitology* 2000; **86**: 526–530.
161. Consalter A, Frazao-Teixeira E, Dubey J, Zanella E, da Silva A, de Souza G *et al.* Epidemiological Investigation of *Toxoplasma gondii* Infections in Commercial Sheep Flock in an Endemic Area for Ocular Toxoplasmosis in Southern Brazil. *Acta Parasitologica* 2019; **64**: 514–519.
162. Correa R, Cedeño I, de Escobar C, Fuentes I. Increased urban seroprevalence of *Toxoplasma gondii* infecting swine in Panama. *Veterinary Parasitology* 2008; **153**: 9–11.
163. Correia E, Feitosa T, dos Santos F, de Azevedo S, Pena H, Gennari S *et al.* Prevalence and risk factors for *Toxoplasma gondii* in sheep in the State of Paraiba, Northeastern Brazil. *Revista Brasileira de Parasitologia Veterinaria* 2015; **24**: 383–386.
164. Cosendey-KezenLeite R, de Oliveira F, Frazao-Teixeira E, Dubey J, de Souza G, Ferreira A *et al.* Occurrence and risk factors associated to *Toxoplasma gondii* infection in sheep from Rio de Janeiro, Brazil. *Tropical Animal Health and Production* 2014; **46**: 1463–1466.
165. Costa, G., da Costa, A., Lopes, W., Bresciani, K., dos Santos, T., Esper, C., & Santana, A. (2011). *Toxoplasma gondii*: Infection natural congenital in cattle and an experimental inoculation of gestating cows with oocysts. *Experimental Parasitology*, *127*(1), 277–281.
166. Costa D, Marvulo M, Silva J, Santana S, Magalhaes F, Lima C *et al.* Seroprevalence of *Toxoplasma gondii* in Domestic and Wild Animals From the Fernando de Noronha, Brazil. *Journal of Parasitology* 2012; **98**: 679–680.
167. Costa-Silva S, Sacristán C, Gonzales-Viera O, Díaz-Delgado J, Sánchez-Sarmiento AM, Marigo J *et al.* *Toxoplasma gondii* in cetaceans of Brazil: a histopathological and immunohistochemical survey. *Revista Brasileira de Parasitologia Veterinária* 2019; **28**: 395–402.
168. Cotey SR, Scimeca R, Chang L, Carpenter AL, Will EE, Ott-Conn C *et al.* *Toxoplasma gondii* Prevalence, Partial Genotypes, and Spatial Variation In North American River Otters (*Lontra canadensis*) In The Upper Peninsula of Michigan, USA. *Journal of Wildlife Diseases* 2022; **58**: 869–881.
169. Cox JC, Edmonds JW, Rosamond CH. Toxoplasmosis and the wild rabbit *Oryctolagus cuniculus* in Victoria, Australia with suggested mechanisms for dissemination of oocysts. *Journal of Hygiene* 1981; **87**: 331–337.
170. Cox JJ, Slabach B, Hast JT, Murphy SM, Kwok OCHH, Dubey JP. High seroprevalence of *Toxoplasma gondii* in elk (*Cervus canadensis*) of the central Appalachians, USA. *Parasitology Research* 2017; **116**: 1079–1083.
171. Crist SC, Stewart RL, Rinehart JP, Needham GR. Surveillance for *Toxoplasma gondii* in the white-tailed deer (*Odocoileus virginianus*) in Ohio. *Ohio Journal of Science* 1999; **99**: 34–37.
172. Cruciani D, Crotti S, Paoloni D, La Morgia V, Felici A, Papa P *et al.* Health Status of the Eastern Grey Squirrel (*Sciurus carolinensis*) Population in Umbria: Results of the LIFE Project ‘U-SAVEREDS’. *Animals* 2022; **12**: 2741.
173. Cruz-Vázquez C, De Velasco-Reyes I, Vitela-Mendoza I. Seroprevalence of *Toxoplasma gondii* Infection and Associated Factors In Sheep From Jalisco, Mexico. *Journal of Parasitology* 2020; **106**: 392–394.
174. Cubas-Atienzar A, Hide G, Smith J. Mat Seroprevalence Infers Low Rates of *Toxoplasma gondii* In Domestic Pigs From Yucatan, Mexico. *Journal of Parasitology* 2019; **105**: 738–747.
175. Curi NH de A, Coelho CM, Malta M de CC, Magni EMV, Sábato MAL, Araújo AS *et al.* Pathogens of Wild Maned Wolves (*Chrysocyon brachyurus*) in Brazil. *Journal of Wildlife Diseases* 2012; **48**: 1052–1056.
176. da Silva J, Alves B, Melo R, Kim P, Neto O, Bezerra M *et al.* Occurrence of anti-*Toxoplasma gondii* antibodies and parasite DNA in raw milk of sheep and goats of local breeds reared in Northeastern Brazil. *Acta Tropica* 2015; **142**: 145–148.
177. da Silva MA, Pena HFJ, Soares HS, Aizawa J, Oliveira S, Alves BF *et al.* Isolation and genetic characterization of *Toxoplasma gondii* from free-ranging and captive birds and mammals in Pernambuco state, Brazil. *Rev Bras Parasitol Vet* 2018; **27**: 481–487.
178. da Silva R, Langoni H, Su C, da Silva A. Genotypic characterization of *Toxoplasma gondii* in sheep from Brazilian slaughterhouses: New atypical genotypes and the clonal type II strain identified. *Veterinary Parasitology* 2011; **175**: 173–177.
179. da Silva RC, Machado GP, Cruvinel TM de A, Cruvinel CA, Langoni H. Detection of antibodies to *Toxoplasma gondii* in wild animals in Brazil. *Journal of Venomous Animals and Toxins Including Tropical Diseases* 2014; **20**: 1–4.
180. da Silva RC, Machado GP, de Andrade Cruvinel TM, Cruvinel CA, Langoni H. Frequency of *Toxoplasma gondii* antibodies in tufted capuchin monkeys (*Cebus apella nigritus*) from an ecological station in the state of São Paulo, Brazil. *Pesquisa Veterinaria Brasileira* 2013; **33**: 251–253.
181. da Silva RC, Zetun CB, Bosco S de MG, Bagagli E, Rosa PS, Langoni H. *Toxoplasma gondii* and Leptospira spp. infection in free-ranging armadillos. *Veterinary Parasitology* 2008; **157**: 291–293.
182. da Silva Yang SGN, da Silva e Souza D, da Silva Santiago AC, Silva RBS, de Oliveira PRF, Mota RA *et al.* Molecular and serological detection of *Leishmania infantum*, *Toxoplasma gondii*, and Leptospira spp. in free-ranging capybaras (*Hydrochoerus hydrochaeris*) from the Atlantic Forest. *Eur J Wildl Res* 2021; **67**: 13.
183. Dabritz HA, Miller MA, Gardner IA, Packham AE, Atwill ER, Conrad PA. Risk Factors for *Toxoplasma gondii* infection in wild rodents from central coastal California and a review of *T. gondii* prevalence in rodents. *Journal of Parasitology* 2008; **94**: 675–683.
184. Dahmani A, Harhoura K, Aissi M, Zenia S, Hamriouri B, Guechi N *et al.* The zoonotic protozoan of sheep carcasses in the north of Algeria: A case of ovine toxoplasmosis. *Journal of The Hellenic Veterinary Medical Society* 2018; **69**: 1016–1024.
185. Dahourou L, Gbati O, Savadogo M, Yougbare B, Dicko A, Combari A *et al.* Prevalence of *Toxoplasma gondii* and *Neospora caninum* infections in households sheep ‘Elevage en case’ in Dakar, Senegal. *Veterinary World* 2019; **12**: 1028–1032.
186. Damriyasa IM, Bauer C. Seroprevalence of *Toxoplasma gondii* infection in sows in Münsterland, Germany. *Dtsch Tierarztl Wochenschr* 2005; **112**: 223–224.
187. Darabus G, Hotea I, Oprescu I, Morariu S, Brudiu I, Olariu R. Toxoplasmosis seroprevalence in cats and sheep from Western Romania. *Revue De Medecine Veterinaire* 2011; **162**: 316–320.
188. Davies PR, Morrow WEM, Deen J, Gamble HR, Patton S. Seroprevalence of *Toxoplasma gondii* and *Trichinella spiralis* in finishing swine raised in different production systems in North Carolina, USA. *Preventive Veterinary Medicine* 1998; **36**: 67–76.
189. Davoust B, Mediannikov O, Roqueplo C, Perret C, Demoncheaux J-P, Sambou M *et al.* Serological survey of animal toxoplasmosis in Senegal. *Bulletin de la Societe de pathologie exotique (1990)* 2015; **108**: 73–7.
190. de Azevedo, P., Ribeiro-Andrade, M., dos Santos, J., dos Reis, A., Valença, S., Fernandes, E., Pinheiro, J., & Mota, R. (2020). Serological survey and risk factors for *Toxoplasma gondii* infection in cattle from Amazonas, Brazil. *Preventive Veterinary Medicine*, *176*.
191. De Craeye S, Speybroeck N, Baert K, Ajzenberg D, Darde M, Collinet F *et al.* Prevalence of *Toxoplasma gondii* in Belgian wildlife. In: *8th European Vertebrate Pest Management Conference Prevalence*. 2011, p 146527.
192. De Craeye S, Speybroeck N, Ajzenberg D, Dardé MLL, Collinet F, Tavernier P *et al.* *Toxoplasma gondii* and *Neospora caninum* in wildlife: Common parasites in Belgian foxes and Cervidae? *Veterinary Parasitology* 2011; **178**: 64–69.
193. de Jesus RF, Rodrigues GM, Silva EM, Carneiro AJB, Franke CR, de Magalhães Cunha R *et al.* Toxoplasmatinae Parasites in Bats from Bahia State, Brazil. *J Wildl Dis* 2017; **53**: 144–147.
194. de Lima D, de Melo RPB, Campos de Almeida J, Rodrigues Magalhães FJ, Ribeiro andrade M, de Morais Pedrosa C *et al.* *Toxoplasma gondii* in invasive animals on the Island of Fernando de Noronha in Brazil: Molecular characterization and mouse virulence studies of new genotypes. *Comparative Immunology, Microbiology and Infectious Diseases* 2019; **67**: 101347.
195. de Macedo M, de Macedo C, Ewald M, Martins G, Zulpo D, da Cunha I *et al.* Isolation and genotyping of *Toxoplasma gondii* from pregnant dairy cows (*Bos taurus*) slaughtered. *Revista Brasileira de Parasitologia Veterinaria* 2012; **21**: 74–77.
196. de Oliveira F, de Oliveira P, da Cunha N, Aguiar C, Pappen F, Ruas J *et al.* The incidence and productive significance of ovine toxoplasmosis in Southern Brazil. *Ciencia Rural* 2016; **46**: 1618–1621.
197. de Sousa R, Lemos J, Farias L, Lopes C, dos Santos K. Seroprevalence and risk factors for *Toxoplasma gondii* infection in pigs in southern Piaui. *Revista Brasileira de Parasitologia Veterinaria* 2014; **23**: 98–100.
198. de Sousa S de, Ajzenberg D, Canada N, Freire L, Costa JMC da, Dardé ML *et al.* Biologic and molecular characterization of *Toxoplasma gondii* isolates from pigs from Portugal. *Veterinary Parasitology* 2006; **135**: 133–136.
199. de Souza JBR, Soares VE, Maia MO, Pereira CM, Ferraudo AS, Cruz BC *et al.* Spatial distribution and risk factors for *Toxoplasma gondii* seropositivity in cattle slaughtered for human consumption in Rondônia, North region, Brazil. *Veterinary Parasitology* 2016; **226**: 145–149.
200. de Thoisy B de, Demar M, Aznar C, Carme B, Mammals N. Ecologic correlates of *Toxoplasma gondii* exposure in free-ranging neotropical mammals. *Journal of Wildlife Diseases* 2003; **39**: 456–459.
201. de Thoisy B de, Vogel I, Reynes JM, Pouliquen JF, Carme B, Kazanji M *et al.* Health evaluation of translocated free-ranging primates in French Guiana. *American Journal of Primatology* 2001; **54**: 1–16.
202. Dechicha AS, Bachi F, Gharbi I, Gourbdji E, Baazize-Ammi D, Brahim-Errahmani O *et al.* Sero-epidemiological survey on toxoplasmosis in cattle, sheep and goats in Algeria. *AJAR* 2015; **10**: 2113–2119.
203. Deem SL, Emmons LH. Exposure of Free-Ranging Maned Wolves (*Chrysocyon brachyurus*) To Infectious and Parasitic Disease Agents in the Noël Kempff Mercado National Park, Bolivia. *Journal of Zoo and Wildlife Medicine* 2005; **36**: 192–197.
204. Dehkordi F, Rahimi E, Abdizadeh R. Detection of *Toxoplasma gondii* in Raw Caprine, Ovine, Buffalo, Bovine, and Camel Milk Using Cell Cultivation, Cat Bioassay, Capture ELISA, and PCR Methods in Iran. *Foodborne Pathogens and Disease* 2013; **10**: 120–125.
205. Deksne G, Kirjušina M. Seroprevalence of *Toxoplasma gondii* in Domestic Pigs (Sus scrofa domestica) and Wild Boars *(Sus scrofa)* in Latvia. *Journal of Parasitology* 2013; **99**: 44–47.
206. Deksne G, Segliņa Z, Ligere B, Kirjušina M. The Pine marten (*Martes martes*) and the Stone marten (*Martes foina*) as possible wild reservoirs of *Toxoplasma gondii* in the Baltic States. *Veterinary Parasitology: Regional Studies and Reports* 2017; **9**: 70–74.
207. Dellarupe A, Fitte B, Pardini L, Campero LM, Bernstein M, Robles MDR *et al.* *Toxoplasma gondii* and *Neospora caninum* infections in synanthropic rodents from Argentina. *Revista Brasileira de Parasitologia Veterinaria* 2019; **28**: 113–118.
208. Dempster R, Wilkins M, Green R, de Lisle G. Serological survey of *Toxoplasma gondii* and *Campylobacter fetus fetus* in sheep from New Zealand. *New Zealand Veterinary Journal* 2011; **59**: 155–159.
209. Derakhshan M, Mousavi M. Serological survey of antibodies to *Toxoplasma gondii* in cats, goats, and sheep in Kerman, Iran. *Comp Clin Pathol* 2014; **23**: 267–268.
210. Devleesschauwer B, Pruvot M, Joshi DD, De Craeye S, Jennes M, Ale A *et al.* Seroprevalence of Zoonotic Parasites in Pigs Slaughtered in the Kathmandu Valley of Nepal. *Vector-Borne and Zoonotic Diseases* 2013; **13**: 872–876.
211. Diakou A, Elias P, Nikolaos P, Charilaos K, Nektarios G. *Toxoplasma gondii* and *Neospora caninum* seroprevalence in dairy sheep and goats mixed stock farming. *Veterinary Parasitology* 2013; **198**: 387–390.
212. Dideprich V, New JC, Noblet GP, Patton S. Serologic Survey of *Toxoplasma gondii* Antibodies in Free-ranging Wild Hogs *(Sus scrofa)* from the Great Smoky Mountains National Park and From Sites in South Carolina. *The Journal of Eukaryotic Microbiology* 1996; **43**: 122S-122S.
213. Djellata N, Yahimi A, Hanzen C, Saegerman C, Kaidi R. Prevalence and factors associated with a higher or lower risk of exposure to *Coxiella burnetii, Chlamydia abortus* and *Toxoplasma gondii* in dairy cows that have aborted in Algeria. *Revue Scientifique Et Technique-office International Des Epizooties* 2019; **38**: 775–786.
214. Djokic V, Blaga R, Aubert D, Durand B, Perret C, Geers R *et al.* *Toxoplasma gondii* infection in pork produced in France. *Parasitology* 2016; **143**: 557–567.
215. Djokic V, Fablet C, Blaga R, Rose N, Perret C, Djurkovic-Djakovic O *et al.* Factors associated with *Toxoplasma gondii* infection in confined farrow-to-finish pig herds in western France: an exploratory study in 60 herds. *Parasites & Vectors* 2016; **9**. doi:10.1186/s13071-016-1753-5.
216. Doby JM, Desmonts G, Beaucournu JG, Akinchina GT. Recherche immunologique systematique de Toxoplasmose chez les petits mammiferes sauvages en France. *Folia Parasitologica* 1974; **21**: 289–300.
217. Dodd NS, Lord JS, Jehle R, Parker S, Parker F, Brooks DR *et al.* *Toxoplasma gondii*: Prevalence in species and genotypes of British bats (*Pipistrellus pipistrellus and P. pygmaeus*). *Experimental Parasitology* 2014; **139**: 6–11.
218. Dong B, Deng M, Li G, Wang J, Tan Q. Qiannan livestock toxoplasmosis serological survey. *Heilongjiang Animal Science and Veterinary Medicine* 2015;94–95.
219. Dong H, Su R, Lu Y, Wang M, Liu J, Jian F *et al.* Prevalence, Risk Factors, and Genotypes of *Toxoplasma gondii* in Food Animals and Humans (2000-2017) From China. *Frontiers In Microbiology* 2018; **9**. doi:10.3389/fmicb.2018.02108.
220. Dong Y, Luo Z, Zhang G, Liu X. Epidemiological survey of toxoplasmosis in cattle and sheep in Qinghai Province. *Chinese Journal of Zoonoses* 2011; **27**: 359–363.
221. Dorion B, Black W, Wolff P, Murray L, Nomi K, Bildfell R. Seroprevalence of *Toxoplasma gondii* in American Black Bears (*Ursus americanus*) in Nevada, USA, using an Enzyme-linked Immunosorbent Assay. *Journal of Wildlife Diseases* 2021; **57**: 408–412.
222. dos Santos EO, Klain VF, Manrique SB, Roman IJ, dos Santos HF, Sangioni LA *et al.* The Influence of Landscape Structure on the Occurrence of *Neospora caninum*, *Toxoplasma gondii*, and Sarcocystis spp. in Free-Living Neotropical Primates. *Acta Parasit* 2022; **67**: 1680–1696.
223. dos Santos H, Freire R, Merlini L, Sposito P, Lima J, Navarro I. Occurrence of infection by *Toxoplasma gondii* in slaughtered swine in the northwestern region of Parana, Brazil. *Semina-Ciencias Agrarias* 2015; **36**: 1999–2004.
224. dos Santos I, Leite A, Furquim M, Zanatto D, Fernandes S, da Silva G *et al.* Frequency of antibodies and risk factors associated with *Toxoplasma gondii* infection in backyard pig production in the city of Mossoro, state of Rio Grande do Norte, Brazil. *Revista Brasileira de Parasitologia Veterinaria* 2019; **28**: 508–513.
225. Du F, Zhang Q, Yu Q, Hu M, Zhou Y, Zhao J. Soil contamination of *Toxoplasma gondii* oocysts in pig farms in central China. *Veterinary Parasitology* 2012; **187**: 53–56.
226. Dubey AJP, Graham DH, Young RWD, Dahl E, Eberhard ML, Nace EK *et al.* Molecular and Biologic Characteristics of *Toxoplasma gondii* Isolates from Wildlife in the United States. *Journal of Parasitology* 2004; **90**: 67–71.
227. Dubey AJP, Sundar N, Nolden CA, Samuel MD, Velmurugan GV, Kwok OCH *et al.* Characterization of *Toxoplasma gondii* from Raccoons (*Procyon lotor*), Coyotes (*Canis latrans*), and Striped Skunks (*Mephitis mephitis*) in Wisconsin Identified Several Atypical Genotypes. *Journal of Parasitology* 2007; **93**: 1524–1527.
228. Dubey AJP, Velmurugan GV, Ferreira LR, Kwok OCH, Su C. Isolation of *Toxoplasma gondii* from Animals in Durango, Mexico. *Journal of Parasitology* 2009; **95**: 319–322.
229. Dubey JP. *Toxoplasmosis of animals and humans*. 2nd ed. CRC Press, 2010https://www.crcpress.com/Toxoplasmosis-of-Animals-and-Humans-Second-Edition/Dubey/p/book/9781420092363.
230. Dubey JP, Briscoe N, Gamble R, Zarlenga D, Humphreys JG, Thulliez P. Characterization of *Toxoplasma* and *Trichinella* isolates from muscles of black bears in Pennsylvania. *American journal of veterinary research* 1994; **55**: 815–9.
231. Dubey JP, Brown J, Verma SK, Cerqueira-Cézar CK, Banfield J, Kwok OCH *et al.* Isolation of viable *Toxoplasma gondii*, molecular characterization, and seroprevalence in elk (*Cervus canadensis*) in Pennsylvania, USA. *Veterinary Parasitology* 2017; **243**: 1–5.
232. Dubey JP, Cerqueira-Cézar CK, Murata FHA, Verma SK, Kwok OCH, Pedersen K *et al.* White-tailed deer (*Odocoileus virginianus*) are a reservoir of a diversity of *Toxoplasma gondii* strains in the USA and pose a risk to consumers of undercooked venison. *Parasitology* 2020; **147**: 775–781.
233. Dubey JP, Choudhary S, Ferreira LR, Kwok OCH, Butler E, Carstensen M *et al.* Isolation and RFLP genotyping of *Toxoplasma gondii* from the gray wolf (*Canis lupus*). *Veterinary Parasitology* 2013; **197**: 685–690.
234. Dubey JP, Foreyt WJ. Seroprevalence of *Toxoplasma gondii* in Rocky Mountain Bighorn Sheep (*Ovis canadensis*). *Journal of Parasitology* 2000; **86**: 622–623.
235. Dubey JP, Gamble HR, Rodrigues AO, Thulliez PH. Prevalence of antibodies to *Toxoplasma gondii* and *Trichinella spiralis* in 509 pigs from 31 farms in Oahu, Hawaii. *Veterinary Parasitology* 1992; **43**: 57–63.
236. Dubey JP, Hamir AN, Hanlon C, Rupprecht CE. Prevalence of *Toxoplasma gondii* infection in raccoons. *Journal of the American Veterinary Medical Association* 1992; **200**: 534–536.
237. Dubey JP, Humphreys JG, Thulliez P. Prevalence of viable *Toxoplasma gondii* tissue cysts and antibodies to *T. gondii* by various serologic tests in black bears (*Ursus americanus*) from Pennsylvania. *Journal of Parasitology* 1995; **81**: 109–112.
238. Dubey JP, Jenkins MC, Kwok OCH, Zink RL, Michalski ML, Ulrich V *et al.* Seroprevalence of *Neospora caninum* and *Toxoplasma gondii* antibodies in white-tailed deer (*Odocoileus virginianus*) from Iowa and Minnesota using four serologic tests. *Veterinary Parasitology* 2009; **161**: 330–334.
239. Dubey JP, Leighty JC, Beal VC, Anderson WR, Andrews CD, Thulliez Ph. National Seroprevalence of *Toxoplasma gondii* in Pigs. *The Journal of Parasitology* 1991; **77**: 517–521.
240. Dubey JP, Mansfield K, Hall B, Kwok OCH, Thulliez P. Seroprevalence of *Neospora caninum* and *Toxoplasma gondii* in black-tailed deer (*Odocoileus hemionus columbianus*) and mule deer (*Odocoileus hemionus hemionus*). *Veterinary Parasitology* 2008; **156**: 310–313.
241. Dubey JP, Rajendran C, Ferreira LR, Kwok OCH, Sinnett D, Majumdar D *et al.* A New Atypical Highly Mouse Virulent *Toxoplasma gondii* Genotype Isolated from a Wild Black Bear in Alaska. *Journal of Parasitology* 2010; **96**: 713–716.
242. Dubey JP, Rollor EA, Smith K, Kwok OCH, Thulliez P. Low Seroprevalence of *Toxoplasma gondii* in Feral Pigs from a Remote Island Lacking Cats. *The Journal of Parasitology* 1997; **83**: 839.
243. Dubey JP, Thulliez P, Powell EC. *Toxoplasma gondii* in Iowa Sows: Comparison of Antibody Titers to Isolation of *T. gondii* by Bioassays in Mice and Cats. *The Journal of Parasitology* 1995; **81**: 48–53.
244. Dubey JP, Van Why K, Verma SK, Choudhary S, Kwok OCH, Khan A *et al.* Genotyping *Toxoplasma gondii* from wildlife in Pennsylvania and identification of natural recombinants virulent to mice. *Veterinary Parasitology* 2014; **200**: 74–84.
245. Dubey JP, Weigel RM, Siegel AM, Thulliez P, Kitron UD, Mitchell MA *et al.* Sources and Reservoirs of *Toxoplasma gondii* Infection on 47 Swine Farms in Illinois. *The Journal of Parasitology* 1995; **81**: 723–729.
246. Dubey JP, Zarnke R, Thomas NJ, Wong SK, Bonn WV, Briggs M *et al.* *Toxoplasma gondii*, *Neospora caninum*, *Sarcocystis neurona,* and Sarcocystis canis-like infections in marine mammals. *Veterinary Parasitology* 2003; **116**: 275–296.
247. Dubey JP, Van Why K, Verma SK, Choudhary S, Kwok OCH, Khan A *et al.* Genotyping *Toxoplasma gondii* from wildlife in Pennsylvania and identification of natural recombinants virulent to mice. *Veterinary Parasitology* 2014; **200**: 74–84.
248. Dubey JP, Velmurugan GV, Rajendran C, Yabsley MJ, Thomas NJ, Beckmen KB *et al.* Genetic characterisation of *Toxoplasma gondii* in wildlife from North America revealed widespread and high prevalence of the fourth clonal type. *International Journal for Parasitology* 2011; **41**: 1139–1147.
249. Dubey JP. *Toxoplasma gondii* infection in rodents and insectivores from Montana. *Journal of Wildlife Diseases* 1983; **19**: 149–150.
250. Dubey JP, Brown J, Ternent M, Verma SK, Hill DE, Cerqueira-Cézar CK *et al.* Seroepidemiologic study on the prevalence of *Toxoplasma gondii* and *Trichinella* spp. infections in black bears (*Ursus americanus*) in Pennsylvania, USA. *Veterinary Parasitology* 2016; **229**: 76–80.
251. Dubey JP, Verma SK, Calero-Bernal R, Cassinelli AB, Kwok OCH, Why KV *et al.* Isolation and genetic characterization of *Toxoplasma gondii* from black bears (*Ursus americanus*), bobcats (*Lynx rufus*), and feral cats (*Felis catus*) from Pennsylvania. *Journal of Eukaryotic Microbiology* 2015; **62**: 410–415.
252. Dubey J, Gamble H, Hill D, Sreekumar C, Romand S, Thulliez P. High prevalence of viable *Toxoplasma gondii* infection in market weight pigs from a farm in Massachusetts. *Journal of Parasitology* 2002; **88**: 1234–1238.
253. Dubey J, Hill D, Rozeboom D, Rajendran C, Choudhary S, Ferreira L *et al.* High prevalence and genotypes of *Toxoplasma gondii* isolated from organic pigs in northern USA. *Veterinary Parasitology* 2012; **188**: 14–18.
254. Dubey J, Hotea I, Olariu T, Jones J, Darabus G. Epidemiological review of toxoplasmosis in humans and animals in Romania. *Parasitology* 2014; **141**: 311–325.
255. Dubey JP, Storandt STS, Kwok OCH, Thulliez P, Kazacos KR. *Toxoplasma gondii* antibodies in naturally exposed wild coyotes, red foxes, and gray foxes and serologic diagnosis of Toxoplasmosis in red foxes fed *T. gondii* oocysts and tissue cysts. *Journal of Parasitology* 1999; **85**: 240–243.
256. Dunbar MR, Cunningham MW, Roof JC. Seroprevalence of selected disease agents from free-ranging black bears in Florida. *Journal of Wildlife Diseases* 1998; **34**: 612–619.
257. Dzib-Paredes G, Rosado-Aguilar J, Acosta-Viana K, Ortega-Pacheco A, Hernández-Cortázar I, Guzman-Marín E *et al.* Seroprevalence and parasite load of *Toxoplasma gondii* in Mexican hairless pig *(Sus scrofa)* tissues from the Southeast of Mexico. *Veterinary Parasitology* 2016; **229**: 45–49.
258. Ebani VV, Guardone L, Rocchigiani G, Bascherini A, Cagnoli G, Bertelloni F *et al.* Molecular survey on the presence of arthropod-borne bacteria and protozoans in roe deer (*Capreolus capreolus*) and ticks from Central Italy. *Acta Tropica* 2022; **233**: 106586.
259. Ebani VV, Poli A, Rocchigiani G, Bertelloni F, Nardoni S, Papini RA *et al.* Serological survey on some pathogens in wild brown hares (*Lepus europaeus*) in Central Italy. *Asian Pac J Trop Med* 2016; **9**: 465–469.
260. Edelhofer R, Prossinger H. Infection with *Toxoplasma gondii* during Pregnancy: Seroepidemiological Studies in Austria. *Zoonoses and Public Health* 2010; **57**: 18–26.
261. Edelhofer R. Prevalence of antibodies against *Toxoplasma gondii* in pigs in Austria--an evaluation of data from 1982 and 1992. *Parasitol Res* 1994; **80**: 642–644.
262. Edelhofer R, Heppe-Winger EM, Hassl A, Aspock H. Toxoplasma-Infektionen bei jagdbaren Wildtieren in Ostosterreich. Mitt. Osterr. *Ges Tropenmed Parasitol* 1989; **11**: 119–123.
263. Ekanayake DK, Rajapakse RPVJ, Dubey JP, Dittus WPJ. Seroprevalence of *Toxoplasma gondii* in Wild Toque Macaques (*Macaca sinica*) at Polonnaruwa, Sri Lanka. *Journal of Parasitology* 2004; **90**: 870–871.
264. El Nahal HS, Morsy TA, Bassili WR, El Missiry AG, Saleh MSM. Antibodies against three parasites of medical importance in Rattus sp. collected in Giza Governorate, Egypt. *Journal of the Egyptian Society of Parasitology* 1982; **12**.
265. El Safadi D, Chahine D, Al Tarraf A, Raii O, Mesto K, Ismail M *et al.* First report on seroprevalence and risk factors of *Toxoplasma gondii* infection in sheep and goats in North Lebanon. *Journal of Infection In Developing Countries* 2019; **13**: 831–836.
266. El-Gawady H, Abdel-Aal A, Sallam N, Youissif E. Molecular studies on *Toxoplasma gondii* in sheep and goats in Ismailia Province, Egypt. Archives of Infectious Diseases and Therapy, 2(2). *Archives of Infectious Diseases and Therapy* 2018; **2**.
267. Elfadaly HA, Hassanain NA, Shaapan RM, Hassanain MA, Barakat AM, Abdelrahman KA. Molecular detection and genotyping of *Toxoplasma gondii* from Egyptian isolates. *Asian Journal of Epidemiology* 2017; **10**: 37–44.
268. Elfahal AM, Elhassan AM, Hussien MO, Enan KA, Musa AB, El Hussein AM. Seroprevalence of *Toxoplasma gondii* in Dairy Cattle with Reproductive Problems in Sudan. *ISRN veterinary science* 2013; **2013**: 895165.
269. Elias MA, Maranhão L, Rabelo RM, Soares HS, Ferreira-Neto JS, Valsecchi J *et al.* Negative seroprevalence for *Toxoplasma gondii* in free-living primates from Central Amazonia. *Journal of Medical Primatology* 2022; **51**: 246–249.
270. Elliott LF, Boyce WM, Clark RK, Jessup DA. Geographic analysis of pathogen exposure in bighorn sheep (Ovis canadensis). *Journal of Wildlife Diseases* 1994; **30**: 315–318.
271. Esteves F, Aguiar D, Rosado J, Costa ML, de Sousa B, Antunes F *et al.* *Toxoplasma gondii* prevalence in cats from Lisbon and in pigs from centre and south of Portugal. *Veterinary Parasitology* 2014; **200**: 8–12.
272. Etougbétché JR, Houéménou G, Missihoun AA, Gauthier P, Dossou H-J, Galal L *et al.* *Toxoplasma gondii* and *Trypanosoma lewisi* infection in urban small mammals from Cotonou, Benin, with special emphasis on co-infection patterns. 2023; BioRx: 2023.10.15.558972.
273. Eymann J, Herbert CA, Cooper DW, Dubey JP. Serologic Survey for *Toxoplasma gondii* and *Neospora caninum* in the Common Brushtail Possum (*Trichosurus Vulpecula*) From Urban Sydney, Australia. *Journal of Parasitology* 2006; **92**: 267–272.
274. Fajardo H, D’ávila S, Bastos R, Cyrino C, Detoni M, Garcia J *et al.* Seroprevalence and risk factors of Toxoplasmosis in cattle from extensive and semi-intensive rearing systems at Zona da Mata, Minas Gerais state, Southern Brazil. *Parasites & Vectors* 2013; **6**. doi:10.1186/1756-3305-6-191.
275. Fan C-K, Su K-E, Tsai Y-J. Serological Survey of *Toxoplasma gondii* Infection Among Slaughtered Pigs in Northwestern Taiwan. *Journal of Parasitology* 2004; **90**: 653–654.
276. Feitosa T, Vilela V, de Almeida-Netoa J, dos Santo A, de Morais D, Alves B *et al.* High genetic diversity in *Toxoplasma gondii* isolates from pigs at slaughterhouses in Paraiba state, northeastern Brazil: Circulation of new genotypes and Brazilian clonal lineages. *Veterinary Parasitology* 2017; **244**: 76–80.
277. Feitosa T, Vilela V, de Melo L, Neto J, Souto D, de Morais D *et al.* *Toxoplasma gondii* and *Neospora caninum* in slaughtered pigs from Northeast, Brazil. *Veterinary Parasitology* 2014; **202**: 305–309.
278. Felin E, Jukola E, Raulo S, Fredriksson-Ahomaa M. Meat Juice Serology and Improved Food Chain Information as Control Tools for Pork-Related Public Health Hazards. *Zoonoses and Public Health* 2015; **62**: 456–464.
279. Fereig RM, Mahmoud HYAH, Mohamed SGA, AbouLaila MR, Abdel-Wahab A, Osman SA *et al.* Seroprevalence and epidemiology of *Toxoplasma gondii* in farm animals in different regions of Egypt. *Veterinary parasitology, regional studies and reports* 2016; **3–4**: 1–6.
280. Fernández-Aguilar X, Alzaga V, Villanúa D, Cabezón O, García-Bocanegra I, Dubey JP *et al.* Epidemiology and prevalence of *Toxoplasma gondii* infection in the Iberian hare (*Lepus granatensis*). *Veterinary Parasitology* 2013; **196**: 194–198.
281. Fernández-Escobar M, Calero-Bernal R, Benavides J, Regidor-Cerrillo J, Guerrero-Molina M, Gutiérrez-Expósito D *et al.* Isolation and genetic characterization of *Toxoplasma gondii* in Spanish sheep flocks. *Parasites & Vectors* 2020; **13**. doi:10.1186/s13071-020-04275-z.
282. Ferreira RA, Mineo JR, Duarte JM, Silva DA, Patarroyo JH. Toxoplasmosis in naturally infected deer from Brazil. *Journal of Wildlife Diseases* 1997; **33**: 896–899.
283. Ferreira SCM, Torelli F, Klein S, Fyumagwa R, Karesh WB, Hofer H *et al.* Evidence of high exposure to *Toxoplasma gondii* in free-ranging and captive African carnivores. *International Journal for Parasitology: Parasites and Wildlife* 2019; **8**: 111–117.
284. Ferroglio E, Bosio F, Trisciuoglio A, Zanet S. *Toxoplasma gondii* in sympatric wild herbivores and carnivores: Epidemiology of infection in the Western Alps. *Parasites and Vectors* 2014; **7**: 196.
285. Fiorello CV, Robbins RGG, Maffei L, Wade SE. Parasites of Free-Ranging Small Canids and Felids in the Bolivian Chaco. *Journal of Zoo and Wildlife Medicine* 2006; **37**: 130–134.
286. Forman D, West N, Francis J, Guy E. The seroprevalence of *Toxoplasma gondii* in British marine mammals. *Memorias do Instituto Oswaldo Cruz* 2009; **104**: 296–298.
287. Formenti N, Gaffuri A, Trogu T, Viganò R, Ferrari N, Lanfranchi P. Spread and genotype of *Toxoplasma gondii* in naturally infected alpine chamois (*Rupicapra r. rupicapra*). *Parasitology Research* 2016; **115**: 2115–2120.
288. Formenti N, Trogu T, Pedrotti L, Gaffuri A, Lanfranchi P, Ferrari N. *Toxoplasma gondii* infection in alpine red deer (*Cervus elaphus*): Its spread and effects on fertility. *PLOS ONE* 2015; **10**: e0138472.
289. Fornazari F, Teixeira CR, Silva RC da, Leiva M, Almeida SC de, Langoni H. Prevalence of antibodies against *Toxoplasma gondii* among Brazilian white-eared opossums (*Didelphis albiventris*). *Veterinary Parasitology* 2011; **179**: 238–241.
290. Foronda P, Plata-Luis J, Castillo-Figueruelo B del, Fernández-Álvarez Á, Martín-Alonso A, Feliu C *et al.* Serological survey of antibodies to *Toxoplasma gondii* and *Coxiella burnetii* in rodents in north-Western African islands (Canary Islands and Cape Verde). *Onderstepoort Journal of Veterinary Research* 2015; **82**: 1–5.
291. Forrester DJ, Conti JA, Belden RC. Parasites of the Florida Panther (*Felis concolor coryi*). Proc. Helminthol. Soc. Wash. 1985; **52**: 95–97.
292. Franti CE, Riemann HP, Behymer DE, Suther D, Howarth JA, Ruppanner R. Prevalence of *Toxoplasma gondii* antibodies in wild and domestic animals in Northern California. *Journal of the American Veterinary Medical Association* 1976; **169**: 901–906.
293. Frazão-Teixeira E, de Oliveira FCR. Anti-*Toxoplasma gondii* antibodies in cattle and pigs in a highly endemic area for human toxoplasmosis in Brazil. *J Parasitol* 2011; **97**: 44–47.
294. Fredebaugh SL, Mateus-Pinilla NE, McAllister M, Warner RE, Weng H-Y. Prevalence of antibody to *Toxoplasma gondii* in terrestrial wildlife in a natural area. *J Wildl Dis* 2011; **47**: 381–392.
295. Freitas JA, Oliveira JP, Ramos OS, Ishizuka MM. Serologic diagnosis of *Toxoplasma gondii* in swines slaughtered without inspection in Northern Brazil. *Arquivo Brasileiro de Medicina Veterinária e Zootecnia* 2009; **61**: 1230–1232.
296. Frenkel JK, Hassanein KM, Hassanein RS, Brown E, Thulliez P, Nunez RQ- *et al.* Panama: a five-year prospective cohort study of children, cats, rodents, birds, and soil. *Tropical Medicine* 1995; **53**: 458–468.
297. Frey C, Berger-Schoch A, Herrmann D, Schares G, Müller N, Bernet D *et al.* Incidence and genotypes of *Toxoplasma gondii* in the muscles of sheep, cattle and pigs as well as in cat feces in Switzerland. *Schweizer Archiv Fur Tierheilkunde* 2012; **154**: 251–255.
298. Frölich K, Wisser J, Schmüser H, Fehlberg U, Neubauer H, Grunow R *et al.* Epizootiologic and ecologic investigations of European brown hares (*Lepus europaeus*) in selected populations from Schleswig-Holstein, Germany. *J Wildl Dis* 2003; **39**: 751–761.
299. Fujii K, Kakumoto C, Kobayashi M, Saito S, Kariya T, Watanabe Y *et al.* Seroepidemiology of *Toxoplasma gondii* and *Neospora caninum* in Seals around Hokkaido, Japan. *Journal of Veterinary Medical Science* 2007; **69**: 393–398.
300. Furtado MM, Gennari SM, Ikuta CY, Jácomo ATDA, Morais ZMD, Pena HFDJ *et al.* Serosurvey of Smooth Brucella, Leptospira spp. and *Toxoplasma gondii* in Free-Ranging Jaguars (*Panthera onca*) and Domestic Animals from Brazil. *PLoS ONE* 2015; **10**: 1–14.
301. Fusco G, Rinaldi L, Guarino A, Proroga Y, Pesce A, Giuseppina D *et al.* *Toxoplasma gondii* in sheep from the Campania region (Italy). *Veterinary Parasitology* 2007; **149**: 271–274.
302. Gaffuri A, Giacometti M, Tranquillo VM, Magnino S, Cordioli P, Lanfranchi P. Serosurvey of roe deer, chamois and domestic sheep in the central Italian Alps. *Journal of Wildlife Diseases* 2006; **42**: 685–690.
303. Gajadhar AA, Aramini JJ, Tiffin G, Bisaillon J-R. Prevalence of *Toxoplasma gondii* in Canadian Market-Age Pigs. *The Journal of Parasitology* 1998; **84**: 759–763.
304. Galal L, Sarr A, Cuny T, Brouat C, Coulibaly F, Sembène M *et al.* The introduction of new hosts with human trade shapes the extant distribution of *Toxoplasma gondii* lineages. *PLOS Neglected Tropical Diseases* 2019; **13**: e0007435.
305. Galal L, Schares G, Stragier C, Vignoles P, Brouat C, Cuny T *et al.* Diversity of *Toxoplasma gondii* strains shaped by commensal communities of small mammals. *International Journal for Parasitology* 2018; **49**: 267–275.
306. Gamarra JA, Cabezón O, Pabón M, Arnal MC, Luco DF, Dubey JP *et al.* Prevalence of antibodies against *Toxoplasma gondii* in roe deer from Spain. *Veterinary Parasitology* 2008; **153**: 152–156.
307. Gamble HR, Brady RC, Dubey JP. Prevalence of *Toxoplasma gondii* infection in domestic pigs in the New England states. *Veterinary Parasitology* 1999; **82**: 129–136.
308. Ganoe LS, Brown JD, Lovallo MJ, Yabsley MJ, Garrett KB, Thompson AT *et al.* Surveillance for diseases, pathogens, and toxicants of muskrat (*Ondatra zibethicus*) in Pennsylvania and surrounding regions. *PLOS ONE* 2021; **16**: e0260987.
309. Garcelon DK, Wayne RK, Gonzales BJ. A serologic survey of the island fox (*Urocyon littoralis*) on the Channel Islands, California. *Journal of wildlife diseases* 1992; **28**: 223–229.
310. Garcia JL, Svoboda WK, Chryssafidis AL, Malanski LDS, Shiozawa MM, Aguiar LDM *et al.* Sero-epidemiological survey for toxoplasmosis in wild New World monkeys (*Cebus spp.; Alouatta caraya*) at the Paraná river basin, Paraná State, Brazil. *Veterinary Parasitology* 2005; **133**: 307–311.
311. García-Bocanegra I, Cabezón O, Hernández E, Martínez-Cruz M, Martinez-Moreno A, Martínez-Moreno J. *Toxoplasma gondii* in Ruminant Species (Cattle, Sheep, And Goats) From Southern Spain. *Journal of Parasitology* 2013; **99**: 438–440.
312. García-Bocanegra I, Dubey J, Simon-Grifé M, Cabezón O, Casal J, Allepuz A *et al.* Seroprevalence and risk factors associated with *Toxoplasma gondii* infection in pig farms from Catalonia, north-eastern Spain. *Research in Veterinary Science* 2010; **89**: 85–87.
313. García-Bocanegra I, Simon-Grifé M, Sibila M, Dubey J, Cabezón O, Martín G *et al.* Duration of maternally derived antibodies in *Toxoplasma gondii* naturally infected piglets. *Veterinary Parasitology* 2010; **170**: 134–136.
314. García-Bocanegra I, Dubey JP, Martínez F, Vargas A, Cabezón O, Zorrilla I *et al.* Factors affecting seroprevalence of *Toxoplasma gondii* in the endangered Iberian lynx (*Lynx pardinus*). *Veterinary Parasitology* 2010; **167**: 36–42.
315. Garcia-Vazquez Z, Rosario-Crus R, Diaz-Garcia G, Hernandez-Baumgarten O. Seroprevalence of *Toxoplasma gondii* infection in cattle, swine and goats in four Mexican states. *Preventive Veterinary Medicine* 1993; **17**: 127–132.
316. Gates M, Gerhold RW, Wilkes RP, Gulsby WD, Maestas L, Rosypal A *et al.* Parasitology, Virology, and Serology of Free-Ranging Coyotes (*Canis latrans*) from Central Georgia, USA. *Journal of Wildlife Diseases* 2014; **50**: 896–901.
317. Gauss CBL, Dubey JP, Vidal D, Ruiz F, Vicente J, Marco I *et al.* Seroprevalence of *Toxoplasma gondii* in wild pigs *(Sus scrofa)* from Spain. *Veterinary Parasitology* 2005; **131**: 151–156.
318. Gauss CBL, Dubey JP, Vidal D, Cabezón O, Ruiz-Fons F, Vicente J *et al.* Prevalence of *Toxoplasma gondii* antibodies in red deer (*Cervus elaphus*) and other wild ruminants from Spain. *Veterinary Parasitology* 2006; **136**: 193–200.
319. Gaydos JK, Conrad PA, Gilardi KVK, Blundell GM, Ben-David M. Does human proximity affect antibody prevalence in marine-foraging river otters (*Lontra canadensis*)? *Journal of Wildlife Diseases* 2007; **43**: 116–123.
320. Gazzonis A, Villa L, Manfredi M, Zanzani S. Spatial Analysis of Infections by *Toxoplasma gondii* and *Neospora caninum* (Protozoa: Apicomplexa) in Small Ruminants in Northern Italy. *ANIMALS* 2019; **9**. doi:10.3390/ani9110916.
321. Gazzonis A, Marangi M, Villa L, Ragona M, Olivieri E, Zanzani S *et al.* *Toxoplasma gondii* infection and biosecurity levels in fattening pigs and sows: serological and molecular epidemiology in the intensive pig industry (Lombardy, Northern Italy). *Parasitology Research* 2018; **117**: 539–546.
322. Gazzonis A, Veronesi F, Di Cerbo A, Zanzani S, Molineri G, Moretta I *et al.* *Toxoplasma gondii* in small ruminants in Northern Italy - prevalence and risk factors. *Annals of Agricultural and Environmental Medicine* 2015; **22**: 62–68.
323. Gazzonis A, Villa L, Riehn K, Hamedy A, Minazzi S, Olivieri E *et al.* Occurrence of selected zoonotic food-borne parasites and first molecular identification of *Alaria alata* in wild boars *(Sus scrofa)* in Italy. *Parasitology Research* 2018; **117**: 2207–2215.
324. Gazzonis A, Zanzani S, Villa L, Manfredi M. *Toxoplasma gondii* infection in meat-producing small ruminants: Meat juice serology and genotyping. *Parasitology International* 2020; **76**. doi:10.1016/j.parint.2020.102060.
325. Ge W, Sun H, Wang Z, Xu P, Wang W, Mu G *et al.* Prevalence and Genotype of *Toxoplasma gondii* Infection in Cattle from Jilin Province, Northeastern China. *Vector-borne and Zoonotic Diseases* 2014; **14**: 399–402.
326. Gebremedhin E, Gizaw D. Seroprevalence of *Toxoplasma gondii* Infection in Sheep and Goats in Three Districts of Southern Nations, Nationalities and Peoples’ Region of Ethiopia. *World Applied Sciences Journal* 2014; **31**: 1891–1896.
327. Gebremedhin EZ, Kebeta MM, Asaye M, Ashenafi H, Di Marco V, Vitale M. First report on seroepidemiology of *Toxoplasma gondii* infection in pigs in Central Ethiopia. *BMC Vet Res* 2015; **11**: 59.
328. Gebremedhin E, Abdurahaman M, Tessema T, Tilahun G, Cox E, Goddeeris B *et al.* Isolation and genotyping of viable *Toxoplasma gondii* from sheep and goats in Ethiopia destined for human consumption. *Parasites & Vectors* 2014; **7**. doi:10.1186/1756-3305-7-425.
329. Gebremedhin E, Agonafir A, Tessema T, Tilahun G, Medhin G, Vitale M *et al.* Seroepidemiological study of ovine toxoplasmosis in East and West Shewa Zones of Oromia Regional State, Central Ethiopia. *BMC Veterinary Research* 2013; **9**. doi:10.1186/1746-6148-9-117.
330. Gebremedhin E, Agonafir A, Tessema T, Tilahun G, Medhin G, Vitale M *et al.* Some risk factors for reproductive failures and contribution of *Toxoplasma gondii* infection in sheep and goats of Central Ethiopia: A cross-sectional study. *Research in Veterinary Science* 2013; **95**: 894–900.624
331. Gebreyes WA, Bahnson PB, Funk JA, McKean J, Patchanee P. Seroprevalence of Trichinella, Toxoplasma, and Salmonella in Antimicrobial-Free and Conventional Swine Production Systems. *Foodborne Pathogens and Disease* 2008; **5**: 199–203.
332. Gehrt SD, Kinsel MJ, Anchor C. Pathogen dynamics and morbidity of striped skunks in the absence of rabies. *Journal of Wildlife Diseases* 2010; **46**: 335–347.
333. Gennari SM, Ogrzewalska MH, Soares HS, Saraiva DG, Pinter A, Nieri-Bastos FA *et al.* *Toxoplasma gondii* antibodies in wild rodents and marsupials from the Atlantic Forest, state of São Paulo, Brazil. *Brazilian Journal of Veterinary Parasitology* 2015; **24**: 379–382.
334. Gennari SM, Caňón-Franco WA, Yai LEO, Souza SLPD, Santos LC, Farias NAR *et al.* Seroprevalence of *Toxoplasma gondii* antibodies from wild canids from Brazil. *Veterinary Parasitology* 2004; **121**: 337–340.
335. Gerhold R, Saraf P, Chapman A, Zou X, Hickling G, Stiver W *et al.* *Toxoplasma gondii* seroprevalence and genotype diversity in select wildlife species from the southeastern United States. *Parasites & Vectors* 2017; **10**. doi:10.1186/s13071-017-2456-2.
336. Gharekhani, J., & Yakhchali, M. (2020). Risk factors associated to Toxoplasma gondii infection in dairy farms in Hamedan suburb, Iran. *Journal of Parasitic Diseases : Official Organ of the Indian Society for Parasitology*, *44*(1), 116–121.
337. Gharekhani J, Yakhchali M, Esmaeilnejad B, Mardani K, Majidi G, Sohrabi A *et al.* Seroprevalence and Risk Factors of *Neospora caninum* and *Toxoplasma gondii* in Small Ruminants in Southwest of Iran. *Archives of Razi Institute* 2018; **73**: 305–310.
338. Ghoneim N, Shalaby S, Hassanain N, Zeedan G, Soliman Y, Abdalhamed A. Comparative Study Between Serological and Molecular Methods for Diagnosis of Toxoplasmosis in Women and Small Ruminants in Egypt. *Foodborne Pathogens and Disease* 2010; **7**: 17–22.
339. Giagaspero M, Bonfini B, Orusa R, Savini G, Osawa T, Harasawa R. Epidemiological Survey for *Toxoplasma gondii*, *Chlamydia psittaci var. ovis, Mycobacterium paratuberculosis, Coxiella burnetii, Brucella spp., Leptospirosis* and Orf Virus among Sheep from Northern Districts of Japan. *Journal of Veterinary Medical Science* 2013; **75**: 679–684.
340. Gilot-Fromont E, Aubert D, Belkilani S, Hermitte P, Gibout O, Geers R *et al.* Landscape, herd management and within-herd seroprevalence of *Toxoplasma gondii* in beef cattle herds from Champagne-Ardenne, France. *Veterinary Parasitology* 2009; **161**: 36–40.
341. Gisbert Algaba I, Verhaegen B, Murat JB, Coucke W, Mercier A, Cox E *et al.* Molecular Study of *Toxoplasma gondii* Isolates Originating from Humans and Organic Pigs in Belgium. *Foodborne Pathogens and Disease* 2020; **17**: 316–321.
342. Glor S, Edelhofer R, Grimm F, Deplazes P, Basso W. Evaluation of a commercial ELISA kit for detection of antibodies against *Toxoplasma gondii* in serum, plasma and meat juice from experimentally and naturally infected sheep. *Parasites & Vectors* 2013; **6**. doi:10.1186/1756-3305-6-85.
343. Goldstein T, Gill VA, Tuomi P, Monson D, Burdin A, Conrad PA *et al.* Assessment of clinical pathology and pathogen exposure in sea otters (*Enhydra lutris*) bordering the threatened population in Alaska. *Journal of Wildlife Diseases* 2011; **47**: 579–592.
344. Gong H. Serological detection of abortion disease in sheep in Qilian County of Qinghai Province. *Qinghai J Anim Vet Sci* 2014; **44**: 12–13.
345. Gotteland C, Chaval Y, Villena I, Galan M, Geers R, Aubert D *et al.* Species or local environment, what determines the infection of rodents by *Toxoplasma gondii*? *Parasitology* 2014; **141**: 259–268.
346. Grema C, Hotea I, Imre M, Dărăbuș G, Pascu C, Mariș C. Seroprevalence of Toxoplasmosis and Swine Influenza in Wild Boars. *Sci Parasitology* 2015; **16**: 20–27.
347. Gresham CS, Gresham CA, Duffy MJ, Faulkner CT, Patton S. Increased prevalence of Brucella suis and pseudorabies virus antibodies in adults of an isolated feral swine population in coastal South Carolina. *Journal of Wildlife Diseases* 2002; **38**: 653–656.
348. Gu D, Li H. Serological survey of swine toxoplasmosis. Shandong Anim Sci Vet Med. 2014;7:53-4. *Shandong Anim Sci Vet Med* 2014; : 53–54.
349. Guimarães AM, Bruhn FRP, da Rocha CMBM, de Araújo TH, Mesquita CAM. Seroepidemiology of *Toxoplasma gondii* in Dairy Cows in Southeastern Brazil: Seropositive Cows on All Farms Investigated. *Acta Parasit* 2020; **65**: 628–635.
350. Guimaraes L, Bezerra R, Rocha D, Albuquerque G. Prevalence and risk factors associated with anti-*Toxoplasma gondii* antibodies in sheep from Bahia state, Brazil. *Revista Brasileira de Parasitologia Veterinaria* 2013; **22**: 220–224.
351. Gustafsson K, Uggla A. Serologic survey for *Toxoplasma gondii* infection in the brown hare (*Lepus europaeus P*.) in Sweden. *Journal of Wildlife Diseases* 1994; **30**: 201–204.
352. Halos L, Thébault A, Aubert D, Thomas M, Perret C, Geers R *et al.* An innovative survey underlining the significant level of contamination by *Toxoplasma gondii* of ovine meat consumed in France. *International Journal For Parasitology* 2010; **40**: 193–200.
353. Halová D, Mulcahy G, Rafter P, Turceková L, Grant T, de Waal T. *Toxoplasma gondii* in Ireland: Seroprevalence and Novel Molecular Detection Method in Sheep, Pigs, Deer and Chickens. *Zoonoses and Public Health* 2013; **60**: 168–173.
354. Hamidinejat H, Ghorbanpour M, Nabavi L, Hajikolaie M, Jalali M. Occurrence of anti-*Toxoplasma gondii* antibodies in female cattle in south-west of Iran. *Tropical Animal Health and Production* 2010; **42**: 899–903.
355. Hamilton CM, Gray R, Wright SE, Gangadharan B, Laurenson K, Innes EA. Prevalence of antibodies to *Toxoplasma gondii* and *Neospora caninum* in red foxes (*Vulpes vulpes*) from around the UK. *Veterinary Parasitology* 2005; **130**: 169–173.
356. Hamilton CM, Katzer F, Beierschmitt A, Soto E, Innes EA, Kelly PJ. First report of *Toxoplasma gondii* seroprevalence in wild-caught Caribbean African green monkeys. *Parasites and Vectors* 2014; **7**: 1–4.
357. Hamilton CM, Kelly PJ, Bartley PM, Burrells A, Porco A, Metzler D *et al.* *Toxoplasma gondii* in livestock in St. Kitts and Nevis, West Indies. *Parasites & Vectors* 2015; **8**: 166.
358. Hamilton C, Katzer F, Innes E, Kelly P. Seroprevalence of *Toxoplasma gondii* in small ruminants from four Caribbean islands. *Parasites & Vectors* 2014; **7**. doi:10.1186/1756-3305-7-449.
359. Hammond-Aryee K, van Heiden L, van Heiden P. The prevalence of antibodies to *Toxoplasma gondii* in sheep in the Western Cape, South Africa. *Onderstepoort Journal of Veterinary Research* 2015; **82**. doi:10.4102/ojvr.v82i1.993.
360. Han H. Serological survey of toxoplasmosis in some dairy farms in Suiping County, Henan Province. *China Dairy* 2019; **39**: 50–51.
361. Han J, Sun X, Li B, Duan G, Zou F. Seroprevalence of toxoplasmosis in pigs in Yuxi City, Yunnan Province. *Heilongjiang Anim Sci Vet Med* 2011; **7**: 102–103.
362. Hancock K, Thiele LA, Zajac AM, Elvinger F, Lindsay DS. Prevalence of Antibodies to *Toxoplasma gondii* in Raccoons (*Procyon lotor*) From an Urban Area of Northern Virginia. *Journal of Parasitology* 2005; **91**: 694–695.
363. Hanif M, Tasawar Z. Seroprevalence and Risk Factors Associated With Toxoplasmosis In Sheep In Multan and Khanewal Districts of Punjab (Pakistan). *Journal of Animal and Plant Sciences* 2016; **26**: 1620–1627.
364. Hanni KD, Mazet JAK, Gulland FMD, Estes J, Staedler M, Murray MJ *et al.* Clinical pathology and assessment of pathogen exposure in southern and Alaskan sea otters. *Journal of Wildlife Diseases* 2003; **39**: 837–850.
365. Harrington LA, Gelling M, Simpson V, Harrington A, Macdonald DW. Notes on the health status of free-living, non-native American mink, Neovison vison, in southern England. *Eur J Wildl Res* 2012; **58**: 875–880.
366. Hartley M, English A. A seroprevalence survey of *Toxoplasma gondii* in common wombats (*Vombatus ursinus*). *Eur J Wildl Res* 2005; **51**: 65–67.
367. Hassanain MA, Elfadaly HA, Shaapan R, Hassanain N, Barakat A. Biological Assay of *Toxoplasma gondii* Egyptian Mutton Isolates. *International Journal of Zoological Research* 2011; **7**: 330–337.
368. He Y, Shan L, XuRong L, ZhenBo Q. Serological survey of animal toxoplasmosis in Zhangye city of Gansu province in China. *China Animal Health Inspection* 2016; **33**: 12–13.
369. Heald K-A, Millins C, Kitchener AC, Banyard AC, Hantke G, Sainsbury KA *et al.* Investigating infectious disease threats to the recovery of the European polecat in Britain. *Mamm Biol* 2020; **100**: 439–444.
370. Hecker Y, Masson F, Armendano J, Cora J, Olivares C, Gual I *et al.* Evaluation of frequency of antibodies against *Toxoplasma gondii*, *Neospora caninum* and Sarcocystis spp. and transmission routes in sheep from Humid Pampa, Argentina. *Acta Parasitologica* 2018; **63**: 416–421.
371. Hecker Y, Moore D, Manazza J, Unzaga J, Späth E, Pardini L *et al.* First report of seroprevalence of *Toxoplasma gondii* and *Neospora caninum* in dairy sheep from Humid Pampa, Argentina. *Tropical Animal Health and Production* 2013; **45**: 1645–1647.
372. Heddergott M, Frantz AC, Stubbe M, Stubbe A, Ansorge H, Osten-Sacken N. Seroprevalence and risk factors of *Toxoplasma gondii* infection in invasive raccoons (*Procyon lotor)* in Central Europe. *Parasitology Research* 2017; **116**: 2335–2340.
373. Heddergott M, Steinbach P, Pohl D, Frantz AC. First report on the sero-epidemiology of *Toxoplasma gondii* infection in German roe deer (*Capreolus capreolus*). *Parasite* 2018; **25**: 52.
374. Hejlíček K, Literák I. Prevalence of Toxoplasmosis in Pigs in the Region of South Bohemia. *Acta Vet Brno* 1993; **62**: 159–166.
375. Hejlíček K, Literák I, Nezval J. Toxoplasmosis in wild mammals from the Czech Republic. *Journal of Wildlife Diseases* 1997; **33**: 480–485.
376. Hernández M, Gómez-Laguna J, Tarradas C, Luque I, García-Valverde R, Reguillo L *et al.* A serological Survey of Brucella spp., Salmonella spp., *Toxoplasma gondii* and Trichinella spp. in Iberian Fattening Pigs Reared in Free-Range Systems. *Transboundary and Emerging Diseases* 2014; **61**: 477–481.
377. Hernández-Cortazar I, Acosta-Viana K, Guzman-Marin E, Ortega-Pacheco A, Torres-Acosta J, Jimenez-Coello M. Presence of *Toxoplasma gondii* in Pork Intended for Human Consumption in Tropical Southern Mexico. *FFoodborne Pathogens and Disease* 2016; **13**: 695–699.
378. Herrero L, Gracia M, Pérez-Arquillué C, Lázaro R, Herrera M, Herrera A *et al.* *Toxoplasma gondii*: Pig seroprevalence, associated risk factors and viability in fresh pork meat. *Veterinary Parasitology* 2016; **224**: 52–59.
379. Herrmann DC, Maksimov P, Maksimov A, Sutor A, Schwarz S, Jaschke W *et al.* *Toxoplasma gondii* in foxes and rodents from the German Federal States of Brandenburg and Saxony-Anhalt: Seroprevalence and genotypes. *Veterinary Parasitology* 2012; **185**: 78–85.
380. Herrmann DC, Wibbelt G, Götz M, Conraths FJ, Schares G. Genetic characterisation of *Toxoplasma gondii* isolates from European beavers (*Castor fiber*) and European wildcats (*Felis silvestris silvestris*). *Veterinary Parasitology* 2013; **191**: 108–111.
381. Hidalgo-Hermoso E, Cabello J, Verasay J, Moreira-Arce D, Hidalgo M, Abalos P *et al.* Serosurvey For Selected Parasitic and Bacterial Pathogens In Darwin’s Fox (*Lycalopex fulvipes*): Not Only Dog Diseases Are A Threat. *Journal of Wildlife Diseases* 2021; **58**: 76–85.
382. Hill D, Haley C, Wagner B, Gamble H, Dubey J. Seroprevalence of and Risk Factors for *Toxoplasma gondii* in the US Swine Herd Using Sera Collected During the National Animal Health Monitoring Survey (Swine 2006). *Zoonoses and Public Health* 2010; **57**: 53–59.
383. Hill NJ, Dubey JP, Vogelnest L, Power ML, Deane EM. Do free-ranging common brushtail possums (*Trichosurus vulpecula*) play a role in the transmission of *Toxoplasma gondii* within a zoo environment? *Veterinary Parasitology* 2008; **152**: 202–209.
384. Hill RE, Zimmerman JJ, Wills RW, Patton S, Clark WR. Seroprevalence of antibodies against *Toxoplasma gondii* in free-ranging mammals in Iowa. *Journal of Wildlife Diseases* 1998; **34**: 811–815.
385. Hillman AE, Lymbery AJ, Elliot AD, Thompson RCA. Urban environments alter parasite fauna, weight and reproductive activity in the quenda (*Isoodon obesulus*). *Science of the Total Environment* 2017; **607–608**: 1466–1478.
386. Hillman AE, Lymbery AJ, Elliot AD, Ash AL, Thompson RCA. Parasitic infections of brushtail possums *Trichosurus vulpecula* in urbanised environments and bushland in the greater Perth region, Western Australia. *Wildlife Biology* 2018; **2018**: 1–8.
387. Hirvelä-Koski V. The prevalence of toxoplasma antibodies in swine sera in Finland. *Acta Vet Scand* 1992; **33**: 21–25.
388. Hofmannová L, Juránková J. Survey of *Toxoplasma gondii* and Trichinella spp. in hedgehogs living in proximity to urban areas in the Czech Republic. *Parasitol Res* 2019; **118**: 711–714.
389. Hoghooghi-Rad N, Afraa M. Prevalence of toxoplasmosis in humans and domestic animals in Ahwaz, capital of Khoozestan Province, south-west Iran. *J Trop Med Hyg* 1993; **96**: 163–168.
390. Holec-Gasior L, Dominiak-Górski B, Kur J. First report of seroprevalence of *Toxoplasma gondii* infection in sheep in Pomerania, northern Poland. *Annals of Agricultural and Environmental Medicine* 2015; **22**: 604–607.
391. Holec-Gasior L, Drapala D, Dominiak-Górski B, Kur J. Epidemiological study of *Toxoplasma gondii* infection among cattle in Northern Poland. *Annals of Agricultural and Environmental Medicine* 2013; **20**: 653–656.
392. Holec-Gasior L, Kur J, Hiszczynska-Sawicka E, Drapala D, Dominiak-Górski B, Pejsak Z. Application of recombinant antigens in serodiagnosis of swine toxoplasmosis and prevalence of *Toxoplasma gondii* infection among pigs in Poland. *Polish Journal of Veterinary Sciences* 2010; **13**: 457–464.
393. Hollings T, Jones M, Mooney N, McCallum H. Wildlife disease ecology in changing landscapes: Mesopredator release and toxoplasmosis. *International Journal for Parasitology: Parasites and Wildlife* 2013; **2**: 110–118.
394. Hollis-Etter KM, Anchor CL, Chelsvig JE, Dubey JP, Warner RE. Suburban white-tailed deer seropositive for *Toxoplasma gondii* from Chicago, Illinois. *Parasitology Research* 2019; **118**: 2271–2276.
395. Holzman S, Conroy MJ, Davidson WR. Diseases, parasites and survival of coyotes in south-central Georgia. *Journal of wildlife diseases* 1992; **28**: 572–580.
396. Hong N, Qian D, Zhang D, Hua Y. Serological investigation of pig’s toxoplasmosis in Guizhou province. *China J Vet Parasitol* 2010; **18**: 68–70.
397. Hong SH, Kim HJ, Jeong YI, Cho SH, Lee WJ, Kim JT *et al.* Serological and molecular detection of *Toxoplasma gondii* and *Babesia microti* in the blood of rescued wild animals in Gangwon-do (Province), Korea. *Korean Journal of Parasitology* 2017; **55**: 207–212.
398. Horta MC, Guimarães MF, Arraes-Santos AI, Araujo AC, Dubey JP, Labruna MB *et al.* Detection of anti-*Toxoplasma gondii* antibodies in small wild mammals from preserved and non-preserved areas in the Caatinga biome, a semi-arid region of Northeast Brazil. *Vet Parasitol Reg Stud Reports* 2018; **14**: 75–78.
399. Hoshina T, Fukumoto S, Aonuma H, Saiki E, Hori S, Kanuka H. Seroprevalence of *Toxoplasma gondii* in wild sika deer in Japan. *Parasitology International* 2019; **71**: 76–79.
400. Hosseini SA, Abediankenari S, Amouei A, Sarvi S, Sharif M, Rezaei F *et al.* Seroprevalence of *Toxoplasma gondii* in Wild Rats (*Rattus rattus*) in Northern Iran. *Vet Med Int* 2021; **2021**: 6655696.
401. Hotea I, Oprescu I, Llie M, Balint A, Dărăbus G. Prevalence of *Toxoplasma gondii* infection, by ELISA, in rams in Timis County. *Lucr Stiint Med Vet* 2011; **54**: 325–328.
402. Hotea I, Ilie MS, Imre M, Sorescu D, Indre D, Brudiu I *et al.* *Toxoplasma gondii* seroprevalence in cats and sheep from Caras-Severin county, Romania. *Lucrari Stiintifice - Universitatea de Stiinte Agricole a Banatului Timisoara, Medicina Veterinara* 2012; **45**: 104–109.
403. Hotea I, Oprescu I, Ilie MS, Imre K, Imre M, Dărăbuș G. Seroprevalence of *Toxoplasma gondii* infection in cats and sheep in Arad County. *Lucrari Stiintifice - Universitatea de Stiinte Agricole a Banatului Timisoara, Medicina Veterinara* 2011; **44**: 63–68.
404. Hotea I, Dărăbuş G, Ilie M, Imre K, Oprescu I, Morariu S *et al.* The Identification Of *Toxoplasma gondii* Infection In Sheep By Elisa From Arad County. 2009.
405. Hou Z, Su S, Liu D, Wang L, Jia C, Zhao Z *et al.* Prevalence, risk factors and genetic characterization of *Toxoplasma gondii* in sick pigs and stray cats in Jiangsu Province, eastern China. *Infection Genetics and Evolution* 2018; **60**: 17–25.
406. Houk AE, Goodwin DG, Zajac AM, Barr SC, Dubey JP, Lindsay DS. Prevalence of Antibodies to *Trypanosoma cruzi,* *Toxoplasma gondii*, *Encephalitozoon cuniculi,* *Sarcocystis neurona,* *Besnoitia darlingi,* and *Neospora caninum* in North American Opossums, *Didelphis virginiana*, from southern Louisiana. *Journal of Parasitology* 2010; **96**: 1119–1122.
407. Hove T, Dubey JP. Prevalence of *Toxoplasma gondii* antibodies in sera of domestic pigs and some wild game species from Zimbabwe. *J Parasitol* 1999; **85**: 372–373.
408. Hove T, Lind P, Mukaratirwa S. Seroprevalence of *Toxoplasma gondii* infection in domestic pigs reared under different management systems in Zimbabwe. *Onderstepoort Journal of Veterinary Research* 2005; **72**: 231–237.
409. Hove T, Mukaratirwa S. Seroprevalence of *Toxoplasma gondii* in farm-reared ostriches and wild game species from Zimbabwe. *Acta Tropica* 2005; **94**: 49–53.
410. Howerth EW, Reeves AJ, McElveen MR, Austin FW. Survey for selected diseases in nutria (*Myocastor coypus*) from Louisiana. *Journal of wildlife diseases* 1994; **30**: 450–453.
411. Hua L, Wei D, Wang J, Xiong Q, Feng Z, Liu M *et al.* Serological investigation of swine Toxoplasma disease in North and northeast China. *Chin J Prev Vet Med* 2016; **38**: 124–127.
412. Huang C, Lin Y, Dai A, Li X, Yang X, Yuan Z *et al.* Seroprevalence of *Toxoplasma gondii* infection in breeding sows in Western Fujian Province, China. *Tropical Animal Health and Production*; **42**: 115–118.
413. Huang R, Li X, Wang G, Ma L. Detection of several pathogens about sheep in Gangcha, Qinghai Province. *Chin J Vet Med* 2015; **51**: 46–48.
414. Hueffer K, Holcomb D, Ballweber LR, Gende SM, Blundell G, O’Hara TM. Serologic surveillance of pathogens in a declining harbor seal (*Phoca vitulina*) population in Glacier Bay National Park, Alaska, USA and a reference site. *Journal of Wildlife Diseases* 2011; **47**: 984–988.
415. Humphreys JG, Stewart RL, Dubey JP. Prevalence of *Toxoplasma gondii* antibodies in sera of hunter-killed white-tailed deer in Pennsylvania. *American Journal of Veterinary Research* 1995; **56**: 172–173.
416. Huong LTT, Dubey JP. Seroprevalence of *Toxoplasma gondii* in Pigs From Vietnam. *Journal of Parasitology* 2007; **93**: 951–952.
417. Hůrková L, Modrý D. PCR detection of *Neospora caninum*, *Toxoplasma gondii* and Encephalitozoon cuniculi in brains of wild carnivores. *Veterinary Parasitology* 2006; **137**: 150–154.
418. Hutchinson J, Smith R. Seropositivity to Toxoplasma infection in sheep samples submitted to Animal and Plant Health Agency laboratories between 2005 and 2012. *Veterinary Record* 2015; **176**. doi:10.1136/vr.102114.
419. Hutchinson J, Wear A, Lambton S, Smith R, Pritchard G. Survey to determine the seroprevalence of *Toxoplasma gondii* infection in British sheep flocks. *Veterinary Record* 2011; **169**: 582-U57.
420. Hwang YT, Pitt JA, Quirk TW, Dubey JP. Seroprevalence of *Toxoplasma gondii* in mesocarnivores of the Canadian prairies. *Journal of Parasitology* 2007; **93**: 1370–1373.
421. Ibrahim H, Huang P, Salem T, Talaat R, Nasr M, Xuan X *et al.* Short Report: Prevalence of *Neospora caninum* and *Toxoplasma gondii* Antibodies in Northern Egypt. *American Journal Of Tropical Medicine And Hygiene* 2009; **80**: 263–267.
422. Ibrahim H, Mohamed A, El-Sharaawy A, El-Shqanqery H. Molecular and serological prevalence of *Toxoplasma gondii* in pregnant women and sheep in Egypt. *Asian Pacific Journal Of Tropical Medicine* 2017; **10**: 996–1001.
423. Ichikawa-Seki M, Guswanto A, Allamanda P, Mariamah ES, Wibowo PE, Igarashi I *et al.* Seroprevalence of antibody to TgGRA7 antigen of *Toxoplasma gondii* in livestock animals from Western Java, Indonesia. *Parasitology International* 2015; **64**: 484–486.
424. Inoue I, Leow CS, Husin D, Matsuo K, Darmani P. A Survey of *Toxoplasma gondii* Antibodies In Pigs In Indonesia. *Southeast Asian J Trop Med Public Health* 2001; **32**.
425. Inpankaew T, Pinyopanuwut N, Chimnoi W, Kengradomkit C, Sununta C, Zhang G *et al.* Serodiagnosis of *Toxoplasma gondii* infection in dairy cows in Thailand. *Transboundary And Emerging Diseases* 2010; **57**: 42–45.
426. Iqbal A, Measures L, Lair S, Dixon B. *Toxoplasma gondii* infection in stranded St. Lawrence Estuary beluga Delphinapterus leucas in Quebec, Canada. *Dis Aquat Organ* 2018; **130**: 165–175.
427. Ito K, Abe S, Yamashita R, Sumiyama D, Kanazawa T, Murata K. Prevalence of serum antibodies to *Toxoplasma gondii* in the small Indian mongoose (*Herpestes auropunctatus*) on Amami-Oshima Island, Japan. *J Vet Med Sci* 2020; **82**: 229–231.
428. Izadyar N, Abd Nikfarjam B, Rastaghi A, Alizadeh S, Heydarian P, Saraei M. A serologic study on *Toxoplasma gondii* infection in slaughtered sheep and goats in Qazvin Province, Iran. *Tropical Animal Health and Production* 2019; **51**: 1289–1293.
429. Jackson MH, Hutchison WM, Siim JC. Toxoplasmosis in a wild rodent population of central Scotland and a possible explanation of the mode of transmission. *Journal of Zoology* 1986; **209**: 549–557.
430. Jakubek EB, Bröjer C, Regnersen C, Uggla A, Schares G, Björkman C. Seroprevalences of *Toxoplasma gondii* and *Neospora caninum* in Swedish red foxes (*Vulpes vulpes)*. *Veterinary Parasitology* 2001; **102**: 167–172.
431. Jakubek EB, Farkas R, Pálfi V, Mattsson JG. Prevalence of antibodies against *Toxoplasma gondii* and *Neospora caninum* in Hungarian red foxes (*Vulpes vulpes*). *Veterinary Parasitology* 2007; **144**: 39–44.
432. Jakubek E-B, Mattsson R, Mörner T, Mattsson JG, Gavier-Widén D. Potential application of serological tests on fluids from carcasses: detection of antibodies against *Toxoplasma gondii* and Sarcoptes scabiei in red foxes (*Vulpes vulpes*). *Acta Vet Scand* 2012; **54**: 13.
433. Jensen SKS, Aars J, Lydersen C, Kovacs KM, Åsbakk K. The prevalence of *Toxoplasma gondii* in polar bears and their marine mammal prey: Evidence for a marine transmission pathway? *Polar Biology* 2010; **33**: 599–606.
434. Jeon SH, Yong TS. Serological observation of *Toxoplasma gondii* prevalence in *Apodemus agrarius,* a dominant species of field rodents in Korea. *Yonsei Med J* 2000; **41**: 491–496.
435. Jeong W, Yoon H, Kim YK, Moon OK, Kim DS, An DJ. Prevalence of antibodies to *Toxoplasma gondii* in South Korean wild boar (*Sus scrofa coreanus*). *Journal of Wildlife Diseases* 2014; **50**: 902–905.
436. Jia JL, Gao S, Shi W, Zhang J. Investigation of sheep infectious and parasitic diseases in large-scale farms in Gansu Province. *China Anim Health Inspect* 2009; **26**: 44–46.
437. Jiang HH, Qin SY, Wang W, He B, Hu TS, Wu JM *et al.* Prevalence and genetic characterization of *Toxoplasma gondii* infection in bats in southern China. *Veterinary Parasitology* 2014; **203**: 318–321.
438. Jiang H-H, Huang S-Y, Zhou D-H, Zhang X-X, Su C, Deng S-Z *et al.* Genetic characterization of *Toxoplasma gondii* from pigs from different localities in China by PCR-RFLP. *Parasites & Vectors* 2013; **6**: 227.
439. Jiang H-H, Zhang W, Zhao L, Zhou D, Song H, Deng S-Z *et al.* Seroprevalence of *Toxoplasma gondii* infection in pigs in Jiangxi Province, Southeastern China. *Foodborne Pathog Dis* 2014; **11**: 362–365.
440. Jiang N, Su R, Jian F, Su C, Zhang X, Jiang B *et al.* *Toxoplasma gondii* in lambs of China: Heart juice serology, isolation and genotyping. *International Journal of Food Microbiology*  2020; **322**. doi:10.1016/j.ijfoodmicro.2020.108563.
441. Jiang S, Chen Y, Wang T. Serological survey and study of pig toxoplasmosis in Yichuan County. *J Anim Sci Vet Med* 2009; **28**: 35–36.
442. Jiang Y, Wang Z, Zhang Y, Ye Q, Fu X. Serological investigation of *Toxoplasma gondii* in parts of Northern Xinjiang. *Chinese Journal of Veterinary Medicine* 2015; **51**: 56–57.
443. Jiménez-Martín D, García-Bocanegra I, Almería S, Castro-Scholten S, Dubey JP, Amaro-López MA *et al.* Epidemiological surveillance of *Toxoplasma gondii* in small ruminants in southern Spain. *Preventive Veterinary Medicine* 2020; **183**: 105137.
444. Jin A. Seroprevalence of *Toxoplasma gondii* infection in pigs in Chenduo County, Qinghai Province. *Heilongjiang Anim Sci Vet Med* 2015; **12**: 110–111.
445. Jittapalapong S, Sarataphan N, Maruyama S, Hugot JP, Morand S, Herbreteau V. Toxoplasmosis in rodents: Ecological survey and first evidences in Thailand. *Vector-Borne and Zoonotic Diseases* 2011; **11**: 231–237.
446. Johnson AM, Roberts H, Munday BL. Prevalence of *Toxoplasma gondii* antibody in wild macropods. *Australian Veterinary Journal* 1988; **65**: 199–201.
447. Johnson CK, Tinker MT, Estes JA, Conrad PA, Staedler M, Miller MA *et al.* Prey choice and habitat use drive sea otter pathogen exposure in a resource-limited coastal system. *Proceedings of the National Academy of Sciences of the United States of America* 2009; **106**: 2242–2247.
448. Johnson D, Harms NJ, Latter NC, Elkin BT, Tabel H, Wei G. Serum biochemistry, serology, and parasitology of boreal caribou (*Rangifer tarandus caribou*) in the Northwest Territories, Canada. *Journal of Wildlife Diseases* 2010; **46**: 1096–1107.
449. Jokelainen, P., Tagel, M., Motus, K., Viltrop, A., & Lassen, B. (2017). *Toxoplasma gondii* seroprevalence in dairy and beef cattle: Large-scale epidemiological study in Estonia. *Veterinary Parasitology*, *236*, 137–143.
450. Jokelainen P, Näreaho A, Knaapi S, Oksanen A, Rikula U, Sukura A. *Toxoplasma gondii* in wild cervids and sheep in Finland: North-south gradient in seroprevalence. *Veterinary Parasitology* 2010; **171**: 331–336.
451. Jokelainen P, Velström K, Lassen B. Seroprevalence of *Toxoplasma gondii* in free-ranging wild boars hunted for human consumption in Estonia. *Acta Veterinaria Scandinavica* 2015; **57**. doi:10.1186/s13028-015-0133-z.
452. Jokelainen P, Deksne G, Holmala K, Näreaho A, Laakkonen J, Kojola I *et al.* Free-ranging Eurasian lynx (*Lynx lynx*) as host of *Toxoplasma gondii* in Finland. *Journal of Wildlife Diseases* 2013; **49**: 527–534.
453. Jokelainen P, Isomursu M, Näreaho A, Oksanen A. Natural *Toxoplasma gondii* infections in European brown hares and mountain hares in Finland: Proportional mortality rate, antibody prevalence, and genetic characterization. *Journal of Wildlife Diseases* 2011; **47**: 154–163.
454. Jokelainen P, Velström K, Lassen B. Seroprevalence of *Toxoplasma gondii* in free-ranging wild boars hunted for human consumption in Estonia. *Acta Veterinaria Scandinavica* 2015; **57**: 1–6.
455. Junge RE, Louis EE, Junge RE, Dipl ACZM, Louis EE, Ph D. Biomedical evaluation of black lemurs (*Eulemur macaco macaco*) in Lokobe Reserve, Madagascar. *Journal of Zoo and Wildlife Medicine* 2007; **38**: 67–76.
456. Kamal A, Din J, Kamil =A, Khan M, Bibi H, Faisal S. Seroprevalence of *Toxoplasma gondii* in sheep and buffalo of District Charsadda, Khyber Pakhtunkhwa, Pakistan. 2019. doi:10.12692/ijb/14.3.497-502.
457. Kamani J, Mani A, Egwu G. Seroprevalence of *Toxoplasma gondii* infection in domestic sheep and goats in Borno state, Nigeria. *Tropical Animal Health and Production*; **42**: 793–797.
458. Kaneko F, Kitamura N, Suzuki K, Kato M. Serological survey of antibodies to four pathogens in wild boars in Nagano Prefecture, Japan. *Journal of Veterinary Medical Science* 2022; **84**: 855–859.
459. Kang SW, Doan HTT, Noh JH, Choe SE, Yoo MS, Kim YH *et al.* Seroprevalence of *Toxoplasma gondii* and *Trichinella spiralis* infections in wild boars *(Sus scrofa)* in Korea. *Parasitology International* 2013; **62**: 583–585.
460. Kantzoura V, Diakou A, Kouam M, Feidas H, Theodoropoulou H, Theodoropoulos G. Seroprevalence and risk factors associated with zoonotic parasitic infections in small ruminants in the Greek temperate environment. *Parasitology International* 2013; **62**: 554–560.
461. Kapperud G. Survey for toxoplasmosis in wild and domestic animals from Norway and Sweden. *Journal of Wildlife Diseases* 1978; **14**: 157–162.
462. Karatepe M, Babür C, Karatepe B, Kiliç S, Cakir M. Prevalence of *Toxoplasma gondii* antibodies in Anatolian ground squirrels, squirrels, *Spermophilus xanthophrymnus* (Rodentia: Sciuridae) from Nigde,Turkey. *Revue de Médecine Vétérinaire* 2004; **155**: 530–532.
463. Katzer F, Brülisauer F, Collantes-Fernández E, Bartley P, Burrells A, Gunn G *et al.* Increased *Toxoplasma gondii* positivity relative to age in 125 Scottish sheep flocks; evidence of frequent acquired infection. *Veterinary Research* 2011; **42**. doi:10.1186/1297-9716-42-121.
464. Kelly TR, Sleeman JM. Morbidity and Mortality of Red Foxes (*Vulpes vulpes*) and Gray Foxes (*Urocyon cinereoargenteus)* Admitted to the Wildlife Center of Virginia, 1993–2001. *Journal of Wildlife Diseases* 2003; **39**: 467–469.
465. Khames M, Yekkour F, Fernández-Rubio C, Aubert D, Nguewa P, Villena I. Serological survey of cattle toxoplasmosis in Medea, Algeria. *Veterinary Parasitology- Regional Studies and Reports* 2018; **12**: 89–90.
466. Khan MT, Din JU, Ali S, Yar A, Bibi H, Faisal S. Seroprevalence of *Toxoplasma gondii* infection in cows and goats of district Charsadda, Khyber Pakhtunkhwa, Pakistan. *International Journal of Fauna and Biological Studies* 2018; **5**: 18–22.
467. Khezri, M. Toxoplasmosis in sheep from Kurdistan province, Iran. *Afr J Microbiol Res* 2012; **6**. doi:10.5897/AJMR11.1210.
468. Kijlstra A, Eissen O, Cornelissen J, Munniksma K, Eijck I, Kortbeek T. *Toxoplasma gondii* infection in animal-friendly pig production systems. *Investigative Ophthalmology & Visual Science* 2004; **45**: 3165–3169.
469. Kijlstra A, Meerburg B, Cornelissen J, De Craeye S, Vereijken P, Jongert E. The role of rodents and shrews in the transmission of *Toxoplasma gondii* to pigs. *Veterinary Parasitology* 2008; **156**: 183–190.
470. Kikuchi Y, Chomel BB, Kasten RW, Martenson JS, Swift PK, O’Brien SJ. Seroprevalence of *Toxoplasma gondii* in American free-ranging or captive pumas (*Felis concolor*) and bobcats (*Lynx rufus*). *Veterinary Parasitology* 2004; **120**: 1–9.
471. Kinjo T, Minamoto N, Suzuki J. Serologic studies on five selected zoonoses in wild Japanese serows (*Capricornis crispus*). *Japanese Journal of Veterinary Science* 1987; **49**: 1027–1033.
472. Kirk CM, Amstrup S, Swor R, Holcomb D, O’Hara TM. Morbillivirus and toxoplasma exposure and association with hematological parameters for southern Beaufort Sea Polar bears: Potential response to infectious agents in a sentinel species. *EcoHealth* 2010; **7**: 321–331.
473. Kjær LJ, Jensen LM, Chriél M, Bødker R, Petersen HH. The raccoon dog (*Nyctereutes procyonoides*) as a reservoir of zoonotic diseases in Denmark. *International Journal for Parasitology: Parasites and Wildlife* 2021; **16**: 175–182.
474. Klun I, Djurković-Djaković O, Katić-Radivojević S, Nikolić A. Cross-sectional survey on *Toxoplasma gondii* infection in cattle, sheep and pigs in Serbia: Seroprevalence and risk factors. *Veterinary Parasitology* 2006; **135**: 121–131.
475. Klun I, Vujanić M, Yera H, Nikolić A, Ivović V, Bobić B *et al.* *Toxoplasma gondii* infection in slaughter pigs in Serbia: seroprevalence and demonstration of parasites in blood. *Veterinary Research* 2011; **42**: 17.
476. Kobayashi S, Shimizu Y, Yamamoto T, Hayama Y, Yamaguchi E, Hanafusa Y *et al.* First nationwide survey of the seroprevalence of *Toxoplasma gondii* in wild boars in Japan. *Parasitol Res* 2021; **120**: 1505–1509.
477. Kocan AA, Barron SJ, Fox JC, Franzmann AW. Antibodies to *Toxoplasma gondii* in moose (*Alces alces* L.) from Alaska. *Journal of wildlife diseases* 1986; **22**: 432.
478. Kofoed KG, Vorslund-Kiar M, Nielsen HV, Alban L, Johansen MV. Sero-prevalence of *Toxoplasma gondii* in Danish pigs. *Veterinary parasitology, regional studies and reports* 2017; **10**: 136–138.
479. Kolören Z, Cerqueira-Cézar CK, Murata FHA, Kwok OCH, Banfield JE, Brown JD *et al.* High Seroprevalence but Low Rate of Isolation of *Toxoplasma gondii* from Wild Elk (*Cervus canadensis*) in Pennsylvania. *Journal of Parasitology* 2019; **105**: 890–892.
480. Kornacka A, Cybulska A, Bień J, Goździk K, Moskwa B. The usefulness of direct agglutination test, enzyme-linked immunosorbent assay and polymerase chain reaction for the detection of *Toxoplasma gondii* in wild animals. *Veterinary Parasitology* 2016; **228**: 85–89.
481. Kornacka A, Cybulskaa A, Popiołekb M, Kuśmierek N, Moskwa B. Survey of *Toxoplasma gondii* and *Neospora caninum* in raccoons (*Procyon lotor*) from the Czech Republic, Germany and Poland. *Veterinary Parasitology* 2018; **262**: 47–50.
482. Krijger IM, Ahmed AAA, Goris MGA, Cornelissen JBWJ, Groot Koerkamp PWG, Meerburg BG. Wild rodents and insectivores as carriers of pathogenic Leptospira and *Toxoplasma gondii* in The Netherlands. *Veterinary Medicine and Science* 2020; **6**: 623–630.
483. Krijger IM, Cornelissen JB, Wisselink HJ, Meerburg BG. Prevalence of *Toxoplasma gondii* in common moles (*Talpa europaea*). *Acta Veterinaria Scandinavica* 2014; **56**: 48.
484. Krücken J, Blümke J, Maaz D, Demeler J, Ramünke S, Antolová D *et al.* Small rodents as paratenic or intermediate hosts of carnivore parasites in Berlin, Germany. *PLOS ONE* 2017; **12**: e0172829.
485. Kunic JM, Bernstein M, Venturini MC, Pardini L, Sommerfelt IE. Risk factors associated with *Toxoplasma gondii* seroprevalence in domestic pig farms in Argentina. *Veterinary Parasitology: Regional Studies and Reports* 2022; **30**: 100710.
486. Kuraa H, Malek S. Seroprevalence of *Toxoplasma gondii* in ruminants by using latex agglutination test (LAT) and enzyme-linked immunosorbent assay (ELISA) in Assiut governorate. *Tropical Biomedicine* 2016; **33**: 711–725.
487. Kurth K, Jiang T, Muller L, Su C, Gerhold RW. *Toxoplasma gondii* contamination at an animal agriculture facility: Environmental, agricultural animal, and wildlife contamination indicator evaluation. *International Journal for Parasitology: Parasites and Wildlife* 2021; **16**: 191–198.
488. Kuruca L, Klun I, Uzelac A, Nikolic A, Bobic B, Simin S *et al.* Detection of *Toxoplasma gondii* in naturally infected domestic pigs in Northern Serbia. *Parasitology Research* 2017; **116**: 3117–3123.
489. Kuruca L, Klun I, Uzelac A, Nikolić A, Bobić B, Simin S *et al.* Detection of Viable in Free-Range Pigs from the Special Nature Reserve of Zasavica. *Contemporary Agriculture* 2016; **65**: 1–6.
490. Kutz SJ, Elkin BT, Panayi D, Dubey JP. Prevalence of *Toxoplasma gondii* Antibodies in Barren-Ground Caribou (*Rangifer tarandus groenlandicus*) from the Canadian Arctic. *The Journal of Parasitology* 2001; **87**: 439.
491. Kutz, S. J., Elkin, B., Gunn, A., & Dubey, J. P. (2000). Prevalence of Toxoplasma gondii Antibodies in Muskox (Ovibos moschatus) Sera from Northern Canada. *Journal of Parasitology*, *86*(4), 879–882
492. Labelle AP, Dubey JP, Mikaelian I, Blanchette N, Lafond R, Angus KW *et al.* Seroprevalence of antibodies to *Toxoplasma gondii* in Lynx (*Lynx canadensis*) and Bobcats (*Lynx rufus*) from Québec, Canada. *Journal of Parasitology* 2001; **87**: 1194–1196.
493. Lafrance-Girard C, Arsenault J, Thibodeau A, Opsteegh M, Avery B, Quessy S. *Toxoplasma gondii* in Retail Beef, Lamb, and Pork in Canada: Prevalence, Quantification, and Risk Factors from a Public Health Perspective. *Foodborne Pathogens and Disease* 2018; **15**: 798–808.
494. Lahmar I, Lachkhem A, Slama D, Sakly W, Haouas N, Gorcii M *et al.* Prevalence of Toxoplasmosis in Sheep, Goats and Cattle in Southern Tunisia. *Journal of Bacteriology & Parasitology* 2015; **06**. doi:10.4172/2155-9597.1000245.
495. Lambourn DM, Jeffries SJ, Dubey JP. Seroprevalence of *Toxoplasma gondii* in Harbor Seals (*Phoca vitulina*) in Southern Puget Sound, Washington. *The Journal of Parasitology* 2001; **87**: 1196.
496. Langoni H, Greca H, Guimaraes F, Ullmann L, Gaio F, Uehara R *et al.* Serological profile of *Toxoplasma gondii* and *Neospora caninum* infection in commercial sheep from Sao Paulo State, Brazil. *Veterinary Parasitology* 2011; **177**: 50–54.
497. Larkin JL, Gabriel M, Gerhold RW, Yabsley MJ, Wester JC, Humphreys JG *et al.* Prevalence to *Toxoplasma gondii* and Sarcocystis spp. in a Reintroduced Fisher (*Martes pennanti*) Population in Pennsylvania. *Journal of Parasitology* 2011; **97**: 425–429.
498. Lashari M, Tasawar Z. Seroprevalence of Toxoplasmosis in Sheep in Southern Punjab, Pakistan. *Pakistan Veterinary Journal* 2010; **30**: 91–94.
499. Laubach ZM, Gering E, Yang E, Montgomery TM, Getty T, Holekamp KE. Associations between *Toxoplasma gondii* infection and steroid hormone levels in spotted hyenas. *International Journal for Parasitology: Parasites and Wildlife* 2022; **17**: 53–59.
500. Leblebicier A, Yildiz K. Seroprevalence of *Toxoplasma gondii* in sheep in Silopi district by using indirect fluorescent antibody test (IFAT). *Turkiye parazitolojii dergisi* 2014; **38**: 1–4.
501. Lehmann T, Graham DH, Dahl E, Sreekumar C, Launer F, Corn JL *et al.* Transmission dynamics of *Toxoplasma gondii* on a pig farm. *Infection, Genetics and Evolution* 2003; **3**: 135–141.
502. Lehrer EW, Fredebaugh SL, Schooley RL, Mateus-Pinilla NE. Prevalence of antibodies to *Toxoplasma gondii* in woodchucks across an urban-rural gradient. *Journal of Wildlife Diseases* 2010; **46**: 977–980.
503. Lei C, Bian S, Gao D, Bao Z, Cai Y. Serological survey of toxoplasmosis of sheep in Aletai region of Xinjiang. *Anim Husb Feed Sci* 2014; **35**: 99–100.
504. Leite TNB, Maja TDA, Ovando TM, Cantadori DT, Schimidt LR, Guercio AC *et al.* Occurrence of infection Leishmania spp. and *Toxoplasma gondii* in monkeys (*Cebus apella*) from Campo Grande, MS. *Revista brasileira de parasitologia veterinaria = Brazilian journal of veterinary parasitology : Orgao oficial do Colegio Brasileiro de Parasitologia Veterinaria* 2008; **17 Suppl 1**: 307–310.
505. Li B, Oledzka G, McFarlane R, Spellerberg M, Smith S, Gelder F *et al.* Immunological response of sheep to injections of plasmids encoding *Toxoplasma gondii* SAG1 and ROP1 genes. *Parasite Immunology* 2010; **32**: 671–683.
506. Li J, Zheng B, Ren H, Wang Z, Yao Z, Wang D. Analysis of seroprevalence and risk factors associated with *Toxoplasma gondii* infection in Xinxiang, Henan Province. *Journal of Medical Pest Control* 2018; **34**: 922–924.
507. Li K, Han Z, Gao J, Liu M, Zhang D, Li J. Seroprevalence of *Toxoplasma gondii* infection in cattle in some counties of Tibet, China. *China Dairy Cattle* 2014; **18**: 24–26.
508. Li Q, Qin S, Li S, Peng P, Zhao Q, Jia H *et al.* First Report on *Toxoplasma gondii* Seroprevalence in Free-Ranging Pigs in Northeastern China. *Acta Parasitologica* 2019; **64**: 295–299.
509. Li R, Li K, Wang X, Luo H, Qiu G, Zhang H *et al.* Seroprevalence of *Toxoplasma gondii* Infection in Tibetan Pigs in Nyingchi, Tibet, China. *Pakistan Journal of Zoology* 2017; **49**: 383–385.
510. Li Y-N, Nie X, Peng Q-Y, Mu X-Q, Zhang M, Tian M-Y *et al.* Seroprevalence and genotype of *Toxoplasma gondii* in pigs, dogs and cats from Guizhou province, Southwest China. *Parasites Vectors* 2015; **8**: 214.
511. Liao G, Liu C, Xiao J, Chen M, Li Y, Chen M. Seroprevalence of *Toxoplasma gondii* infection in pigs in Yongzhou, Hunan. *Anim Husb Vet Sci Tech Inf* 2016; **7**: 22–23.
512. Liassides M, Christodoulou V, Moschandreas J, Karagiannis C, Mitis G, Koliou M *et al.* Toxoplasmosis in female high school students, pregnant women and ruminants in Cyprus. *Transactions of The Royal Society of Tropical Medicine and Hygiene* 2016; **110**: 359–366.
513. Limon G, Beauvais W, Dadios N, Villena I, Cockle C, Blaga R *et al.* Cross-Sectional Study of *Toxoplasma gondii* Infection in Pig Farms in England. *FFoodborne Pathogens and Disease* 2017; **14**: 269-+.
514. Lindsay DS, Spencer J, Rupprecht C, Blagburn BL. Prevalence of agglutinating antibodies to *Neospora caninum* in Raccoons (*Procyon lotor*). *Journal of Parasitology* 2001; **87**: 1197–1198.
515. Lindsay DS, Weston JL, Little SE. Prevalence of antibodies to *Neospora caninum* and *Toxoplasma gondii* in gray foxes (*Urocyon cinereoargenteus*) from South Carolina. *Veterinary Parasitology* 2001; **97**: 159–164.
516. Lindsay DS, Kelly EJ, Mckown RD, Stein FJ, Plozer J, Herman J *et al.* Prevalence of *Neospora caninum* and *Toxoplasma gondii* Antibodies in Coyotes (*Canis latrans*) and Experimental Infections of Coyotes with *Neospora caninum*. *The Journal of Parasitology* 1996; **82**: 657–659.
517. Lindsay DS, McKown RD, DiCristina JA, Jordan CN, Mitchell SM, Oates DW *et al.* Prevalence of Agglutinating Antibodies to *Toxoplasma gondii* in Adult and Fetal Mule Deer (*Odocoileus hemionus*) From Nebraska. *Journal of Parasitology* 2005; **91**: 1490–1491.
518. Lindsay DS, Smith PC, Blagburn BL. Prevalence and isolation of *Toxoplasma gondii* from white-tailed deer in Alabama. *Journal of Parasitology* 1991; **77**: 62–64.
519. Lindsay SA, Caraguel CGB, O’Handley R, Šlapeta J, Gray R. *Toxoplasma gondii* seroprevalence in the endangered Australian sea lion (*Neophoca cinerea*). *Frontiers in Marine Science* 2022; **9**.
520. Litovka D, Naidenko S, Mamaev MS, Klyuchnikova P, Kovekovdova L, Blokhin S *et al.* Monitoring, populational and toxicology researches of gray whales in the Mechigmensky Bay and of beluga whales in the Anadyr Liman (western Bering Sea, Russia)*, 2013-2019 // Paper SC/68b/E/11 presented to the International Whaling Commission Scientific Committee (Cambridge, Great Britain, May, 12-26 2020) - P. 1-6.* 2020.
521. Littnan CL, Stewart BS, Yochem PK, Braun R. Survey for Selected Pathogens and Evaluation of Disease Risk Factors for Endangered Hawaiian Monk Seals in the Main Hawaiian Islands. *EcoHealth* 2006; **3**: 232–244.
522. Liu F, Wang D, Yang S-C, Zhu J-H, Li J-M, Shi K *et al.* Prevalence and Risk Factors of Brucellosis, Toxoplasmosis, and Neosporosis Among Yanbian Yellow Cattle in Jilin Province, China. *Vector Borne Zoonotic Dis* 2019; **19**: 217–221.
523. Liu H. Serological investigation of toxoplasmosis in pigs, cattle and sheep in Wuping County. *Fujian Journal of Animal Husbandry and Veterinary Medicine* 2014; **36**: 16–18.
524. Liu J, Wang P, Wang J, Shayilan K, Jin Y, Lu G. Antibody monitoring and analysis of *Toxoplasma gondii* in partial scale pig farms of Xinjiang. *Progress in Veterinary Medicine* 2016; **37**: 130–132.
525. Liu K. Serological survey of the infection of swine toxoplasmosis in Xinyang. *Journal of Henan Agricultural Sciences* 2013; **42**: 166–168.
526. Liu Q, Ma R, Zhao Q, Shang L, Cai J, Wang X *et al.* Seroprevalence of *Toxoplasma gondii* Infection in Tibetan Sheep in Northwestern China. *Journal of Parasitology* 2010; **96**: 1222–1223.
527. Liu Q, Wang Z-D, Huang S-Y, Zhu X-Q. Diagnosis of toxoplasmosis and typing of *Toxoplasma gondii*. *Parasites & Vectors* 2015; **8**: 292.
528. Liu W, Lu Y, Lu L, Mu Y, Dai L, Song M. Serological investigation of bovine toxoplasmosis in some areas of Heilongjiang Province. *Heilongjiang Animal Science and Veterinary Medicine* 2010: 80–81.
529. Liu X, Liu C, Liu Y, Jin H, Zhao Y, Chen J *et al.* Seroprevalence of *Toxoplasma gondii* Infection in Slaughtered Pigs and Cattle in Liaoning Province, Northeastern China. *Journal of Parasitology* 2012; **98**: 440–441.
530. Liu X-Y, Wang Z-D, El-Ashram S, Liu Q. *Toxoplasma gondii* oocyst-driven infection in pigs, chickens and humans in northeastern China. *BMC Veterinary Research* 2019; **15**: 366.
531. Liu Z, Li J, Pan H. Seroprevalence and risk factors of *Toxoplasma gondii* and *Neospora caninum* infections in small ruminants in China. *Preventative Veterinary Medicine* 2015; **118**: 488–492.
532. Lizana V, Gortázar C, Muniesa A, Cabezón Ó, Martí-Marco A, López-Ramon J *et al.* Human and environmental factors driving *Toxoplasma gondii* prevalence in wild boar *(Sus scrofa)*. *Research in Veterinary Science* 2021; **141**: 56–62.
533. Lobato-Bailón L, López-Morales A, Quintela R, Ribas MP, Molina-López R, Obon E *et al.* Lack of Detection of *Toxoplasma gondii* in Pipistrellus spp. Bats from Densely Cat-Populated Areas of NE Spain. *Pathogens* 2022; **11**: 1451.
534. Lopes AP, Sargo R, Rodrigues M, Cardoso L. High seroprevalence of antibodies to *Toxoplasma gondii* in wild animals from Portugal. *Parasitology Research* 2011; **108**: 1163–1169.
535. Lopes A, Dubey J, Neto F, Rodrigues A, Martins T, Rodrigues M *et al.* Seroprevalence of *Toxoplasma gondii* infection in cattle, sheep, goats and pigs from the North of Portugal for human consumption. *Veterinary Parasitology* 2013; **193**: 266–269.
536. Lopes A, Sargo R, Rodrigues M, Cardoso L. High seroprevalence of antibodies to *Toxoplasma gondii* in wild animals from Portugal. *Parasitology Research* 2011; **108**: 1163–1169.
537. Lopes A, Vilares A, Neto F, Rodrigues A, Martins T, Ferreira I *et al.* Genotyping Characterization of *Toxoplasma gondii* in Cattle, Sheep, Goats and Swine from the North of Portugal. *IRANIAN Journal of Parasitology* 2015; **10**: 465–472.
538. Lopes W, dos Santos T, da Silva R, Rossanese W, de Souza F, Rodrigues J *et al.* Seroprevalence of and risk factors for *Toxoplasma gondii* in sheep raised in the Jaboticabal microregion, Sao Paulo State, Brazil. *Research In Veterinary Science* 2010; **88**: 104–106.
539. Lu Y, Ma L. Serodiagnosis of toxoplasmosis and development of ELISA kit. *Chinese Journal of Animal Health Inspection* 2009; **26**: 40–41.
540. Luciano D, Menezes R, Ferreira L, Nicolau J, das Neves L, Luciano R *et al.* Seroepidemiology of toxoplasmosis in goats and sheep from three counties of Rio de Janeiro state, Brazil. *Pesquisa Veterinaria Brasileira* 2011; **31**: 569–574.
541. Luciano D, Menezes R, Ferreira L, Nicolau J, das Neves L, Luciano R *et al.* Occurrence of anti-*Toxoplasma gondii* antibodies in cattle and pigs slaughtered, State of Rio de Janeiro. *Revista Brasileira de Parasitologia Veterinaria* 2011; **20**: 351–353.
542. Lúcio ÉC, Pimentel J de L, Clemente SM dos S, Sá LMN, Oliveira JMB, Albuquerque PPF *et al.* Epidemiological analysis of *Toxoplasma gondii* infection in sheep in the state of Pernambuco, Brazil. *Semina: Ciências Agrárias* 2017; **38**: 3059–3067.
543. Lukášová R, Marková J, Bártová E, Murat J-B, Sedlák K. Molecular Evidence of *Toxoplasma gondii*, *Neospora caninum*, and *Encephalitozoon cuniculi* in Red Foxes (*Vulpes vulpes*). *Journal of Wildlife Diseases* 2018; **54**: 825–828.
544. Lundén A, Lind P, Gustavsson K, Uggla A, Vågsholm I, Engvall EO. Serological Survey of *Toxoplasma gondii* Infection in Pigs Slaughtered in Sweden. *Scandinavian Journal of Infectious Diseases* 2002; **34**: 362–365.
545. Luo H, Li K, Shahzad M, Zhang H, Lan Y, Xiong X. Seroprevalence of *Toxoplasma gondii* infection in wild boars, wild rabbits, and wild chickens in Hubei Province, China. *Korean Journal of Parasitology* 2017; **55**: 85–88.
546. Luptáková L, Benova K, Rencko A, Petrovova E. DNA detection of *Toxoplasma gondii* in sheep milk and blood samples in relation to phase of infection. *Veterinary Parasitology* 2015; **208**: 250–253.
547. Luptáková L, Bálent P, Válencaková A, Hisira V, Petrovová E. Detection of *Toxoplasma gondii* and encephalitozoon spp. in wild boars by serological and molecular methods. *Revue de Medecine Veterinaire* 2010; **161**: 559–563.
548. Luptáková L, Valencakova A, Bálent P, Malcekova B, Petrovova E. Evaluation of detection of *Toxoplasma gondii* DNA in animal blood samples by quantitative PCR. *Central European Journal of Biology* 2012; **7**. doi:10.2478/s11535-012-0025-4.
549. Lynch M, Nielsen O, Duigna PJ, Kirkwood R, Hoskins A, Arnould JPY. Serologic survey for potential pathogens and assessment of disease risk in Australian fur seals. *Journal of Wildlife Diseases* 2011; **47**: 555–565.
550. Ma L, Li S, Zhang Y, Wen Z. Seroprevalence of *Toxoplasma gondii* and *Neospora caninum* in dairy cows in Hebei province, China. *Animal Biotechnology* 2021; **32**: 451–453.
551. Ma L, Han X, Cai Q, Zheng Y, Lu Y, Wang G *et al.* Serum antibodies detection of *Toxoplasma gondii* in human and animals in some areas of Qinghai Province. *Chin J Vet Med* 2013; **49**: 49–50.
552. Ma R, Cai J, Zhao B, Hu G, Pan X. Serological investigation of toxoplasmosis in cattle and sheep in Delingha region, Qinghai Province. In: *The 11th academic seminar of veterinary parasitology of Chinese Association of Animal Science and Veterinary Medicine.* Wuhan, 2011, p 245.
553. Ma Y, Wang W, Cai X, Liu B, Xie Y. Preliminary investigation on the causes of abortion in cattle and sheep in Huzhu County. *Chinese Qinghai Journal of Animal and Veterinary Science* 2018; **48**: 41–42.
554. Maas M, Glorie J, Dam-Deisz C, de Vries A, Franssen FFJ, Jaarsma RI *et al.* Zoonotic Pathogens in Eurasian Beavers (*Castor fiber*) in the Netherlands. *Journal of Wildlife Diseases* 2022; **58**: 404–408.
555. Macaluso G, Di Bella S, Purpari G, Giudice E, Mira F, Gucciardi F *et al.* Evaluation of a commercial enzyme-linked immunosorbent assay (ELISA) for detecting antibodies against *Toxoplasma gondii* from naturally and experimentally infected pigs. *Infectious Diseases* 2019; **51**: 26–31.
556. Machacová T, Ajzenberg D, Zákovskáb A, Sedlák K, Bártová E. *Toxoplasma gondii* and *Neospora caninum* in wild small mammals: Seroprevalence, DNA detection and genotyping. *Veterinary Parasitology* 2016; **223**: 88–90.
557. Machado DMR, de Barros LD, de Souza Lima Nino B, de Souza Pollo A, dos Santos Silva AC, Perles L *et al.* *Toxoplasma gondii* infection in wild boars *(Sus scrofa)* from the State of São Paulo, Brazil: Serology, molecular characterization, and hunter’s perception on toxoplasmosis. *Veterinary Parasitology: Regional Studies and Reports* 2021; **23**: 100534.
558. Magalhaes F, Ribeiro-andrade M, de Alcantara A, Pinheiro J, de Sena M, Porto W *et al.* Risk factors for *Toxoplasma gondii* infection in sheep and cattle from Fernando de Noronha Island, Brazil. *Revista Brasileira de Parasitologia Veterinaria* 2016; **25**: 511–515.
559. Magalhaes F, Ribeiro-andrade M, Souza F, Lima C, Biondo A, Vidotto O *et al.* Seroprevalence and spatial distribution of *Toxoplasma gondii* infection in cats, dogs, pigs and equines of the Fernando de Noronha Island, Brazil. *Parasitology International* 2017; **66**: 43–46.
560. Mahboub HD, Helal MA, Eldaim M a. A, El-Razek EMA, Elsify AM. Seroprevalence of abortion causing agents in Egyptian sheep and goat breeds and their effects on the animal’s performance. *Journal of Agricultural Science (Toronto)* 2013; **5**: 92–101.
561. Maia JF, Gennari SM, Milanelo L, Furuya HR, Souza VAF, Vitaliano SN. Inquérito sorológico da Toxoplasmose em quatis (*Nasua nasua*) do Parque Ecológico do Tietê, São Paulo, SP, Brasil. *Brazilian Journal of Veterinary Research and Animal Science* 2016; **53**. doi:10.11606/ISSN.1678-4456.BJVRAS.2016.111083.
562. Malmlov A, Breck S, Fry T, Duncan C. Serologic Survey for Cross-Species Pathogens in Urban Coyotes (Canis latrans), Colorado, USA. *Journal of Wildlife Diseases* 2014; **50**: 946–950.
563. Malmsten A, Magnusson U, Ruiz-Fons F, González-Barrio D, Dalin AM. A serologic survey of pathogens in wild boar *(Sus scrofa)* in Sweden. *Journal of Wildlife Diseases* 2018; **54**: 229–237.
564. Malmsten J, Jakubek EB, Björkman C. Prevalence of antibodies against *Toxoplasma gondii* and *Neospora caninum* in moose (*Alces alces*) and roe deer (*Capreolus capreolus*) in Sweden. *Veterinary Parasitology* 2011; **177**: 275–280.
565. Mannise N, Cabrera A, Juan H, Cosse M, Giannitti F, Francia ME *et al.* Molecular detection of coccidian Apicomplexa Parasites isolated from wild crab-eating and pampas foxes through novel TaqMan^TM^ probes: a contribution to their molecular epidemiology. *Mol Biol Rep* 2021; **48**: 5013–5021.
566. Marchiondo AA, Duszynski DW, Maupin GO. Prevalence of antibodies to *Toxoplasma gondii* in wild and domestic animals of New Mexico, Arizona and Colorado. *Journal of Wildlife Diseases* 1976; **12**: 226–232.
567. Marciano MAM, andrade Junior HF de, Meireles LR. Evaluation of the ELISA technique to search for anti-*Toxoplasma gondii* IgG in juices from sun-dried meat. *Brazilian Journal of Food Technology* 2018; **21**: e2017009.
568. Marques J, Isbrecht F, Lucas T, Guerra I, Dalmolin A, da Silva R *et al.* Anti-*Toxoplasma gondii* antibody detection in animals from farms of a rural community of south of Mato Grosso do Sul, Brasil. *Semina-Ciencias Agrarias* 2009; **30**: 889–897.
569. Marques-Santos F, Amendoeira M, Carrijo K, Santos J, Arruda I, Sudré A *et al.* Occurrence of *Toxoplasma gondii* and risk factors for infection in pigs raised and slaughtered in the Triangulo Mineiro region, Minas Gerais, Brazil. *Pesquisa Veterinaria Brasileira* 2017; **37**: 570–576.
570. Martínez-Flores W, Palma-García J, Caballero-Ortega H, Del Viento-Camacho A, López-Escamilla E, Martínez-Hernández F *et al.* Genotyping *Toxoplasma gondii* with the B1 Gene in Naturally Infected Sheep from an Endemic Region in the Pacific Coast of Mexico. *Vector borne and zoonotic diseases* 2017; **17**: 495–502.
571. Martino, P. E., Montenegro, J. L., Preziosi, J. A., Venturini, C., Bacigalupe, D., Stanchi, N. O., & Bautista, E. L. (2004). Serological survey of selected pathogens of free-ranging foxes in southern Argentina, 1998-2001. *OIE Revue Scientifique et Technique*, *23*(3), 801–806.
572. Martino, P. E., Stanchi, N. O., Silvestrini, M., Brihuega, B., Samartino, L., & Parrado, E. (2014). Seroprevalence for selected pathogens of zoonotic importance in wild nutria (*Myocastor coypus*). *European Journal of Wildlife Research*, *60*(3), 551–554.
573. Martino PE, Montenegro JL, Preziosi JA, Venturini C, Bacigalupe D, Stanchi NO *et al.* Serological survey of selected pathogens of free-ranging foxes in southern Argentina, 1998-2001. *OIE Revue Scientifique et Technique* 2004; **23**: 801–806.
574. Martins M, Urbani N, Flanagan C, Siebert U, Gross S, Dubey JP *et al.* Seroprevalence of *Toxoplasma gondii* in Pinnipeds under Human Care and in Wild Pinnipeds. *Pathogens* 2021; **10**: 1415.
575. Mas Bakal PM, Karstad L, In ’T Veld N. Serologic evidence of toxoplasmosis in captive and free-living wild mammals in Kenya. *J Wildl Dis* 1980; **16**: 559–564.
576. Mason S, Quinnell R, Smith J. Detection of *Toxoplasma gondii* in lambs via PCR screening and serological follow-up. *Veterinary Parasitology* 2010; **169**: 258–263.
577. Mathieu A, Flint M, Stent PM, Schwantje HM, Wittum TE. Comparative health assessment of urban and non-urban free-ranging mule deer (*Odocoileus hemionus*) in southeastern British Columbia, Canada. *PeerJ* 2018; **2018**: 1–22.
578. Matsumoto J, Kako Y, Morita Y, Kabeya H, Sakano C, Nagai A *et al.* Seroprevalence of *Toxoplasma gondii* in wild boars (*Sus scrofa leucomystax*) and wild sika deer (*Cervus nippon*) in Gunma Prefecture, Japan. *Parasitology International* 2011; **60**: 331–332.
579. Matsuo K, Kamai R, Uetsu H, Goto H, Takashima Y, Nagamune K. Seroprevalence of *Toxoplasma gondii* infection in cattle, horses, pigs and chickens in Japan. *Parasitology International* 2014; **63**: 638–639.
580. McCauley D, Stout V, Gairhe KP, Sadaula A, Dubovi E, Subedi S *et al.* Serologic Survey of Selected Pathogens in Free-Ranging Bengal Tigers (*Panthera tigris tigris*) in Nepal. *J Wildl Dis* 2021; **57**: 393–398.
581. McCue PM, O’Farrell TP. Serological survey for selected diseases in the endangered San Joaquin kit fox (*Vulpes macrotis mutica)*. *Journal of Wildlife Diseases* 1988; **24**: 274–281.
582. McFadden KW, Wade SE, Dubovi EJ, Gompper ME. A serological and fecal parasitologic survey of the critically endangered pygmy raccoon (*Procyon pygmaeus*). *Journal of Wildlife Diseases* 2005; **41**: 615–617.
583. McGregor G, Gottschalk M, Godson D, Wilkins W, Bollinger T. Disease risks associated with free-ranging wild boar in Saskatchewan. *Canadian Veterinary Journal-Revue Veterinaire Canadienne* 2015; **56**: 839–844.
584. McKay PA, Hufschmid J, Meredith AL, Zendejas-Heredia PA, Moseby KE. Seroprevalence of *Toxoplasma gondii* in burrowing bettongs (*Bettongia lesueur*): a comparison of cat-free and cat-exposed populations. *ajoz* 2022; **69**: 175–183.
585. McKenny L, O’Handley R, Kovaliski J, Mutze G, Peacock D, Lanyon S. Evidence of infection with *Toxoplasma gondii* and *Neospora caninum* in South Australia: using wild rabbits as a sentinel species. *Australian Veterinary Journal* 2020; **98**: 380–387.
586. Measures LN, Dubey JP, Labelle P, Martineau D. Seroprevalence of *Toxoplasma gondii* in Canadian pinnipeds. *Journal of Wildlife Diseases* 2004; **40**: 294–300.
587. Meerburg BG, Van Riel JW, Cornelissen JB, Kijlstra A, Mul MF. Cats and goat whey associated with *Toxoplasma gondii* infection in pigs. *Vector Borne Zoonotic Dis* 2006; **6**: 266–274.
588. Melo RPB, Oliveira PRF, Albuquerque PPF, Barretto MLM, Moura GHF, Oliveira AAF *et al.* Detection of *Toxoplasma gondii* DNA in heart tissue from common marmoset (*Callithrix jacchus*) monitored for yellow fever and rabies in Pernambuco state, Northeastern of Brazil. *Veterinary Parasitology: Regional Studies and Reports* 2020; **21**: 100447.
589. Mendonça C, Barros S, Guimaráes V, Ferraudo A, Munhoz A. Prevalence and risk factors associated to ovine toxoplasmosis in northeastern Brazil. *Revista Brasileira de Parasitologia Veterinaria* 2013; **22**: 230–234.
590. Meng R. Seroepidemiology of *Toxoplasma gondii* in dairy cattle in Xincai County, Henan Province. *China Dairy* 2018; **198**: 46–47.
591. Mercier A, Garba M, Bonnabau H, Kane M, Rossi J-P, Dardé M-L *et al.* Toxoplasmosis seroprevalence in urban rodents: a survey in Niamey, Niger. *Mem Inst Oswaldo Cruz* 2013; **108**: 399–407.
592. Meyer CJ, Cassidy KA, Stahler EE, Brandell EE, Anton CB, Stahler DR *et al.* Parasitic infection increases risk-taking in a social, intermediate host carnivore. *Commun Biol* 2022; **5**: 1–10.
593. Michael S, Howe L, Chilvers B, Morel P, Roe W. Seroprevalence of *Toxoplasma gondii* in mainland and sub-Antarctic New Zealand sea lion *(Phocarctos hookeri*) populations. *New Zealand Veterinary Journal* 2016; **64**: 293–297.
594. Mikaelian I, Boisclair J, Dubey JP, Kennedy S, Martineau D. Toxoplasmosis in Beluga Whales (*Delphinapterus leucas*) from the St Lawrence Estuary: Two case reports and a serological survey. *Journal of Comparative Pathology* 2000; **122**: 73–76.
595. Millán J, Candela MG, Palomares F, Cubero MJ, Rodríguez A, Barral M *et al.* Disease threats to the endangered Iberian lynx (*Lynx pardinus*). *Veterinary Journal* 2009; **182**: 114–124.
596. Millar PR, Daguer H, Vicente RT, Costa T da, Sobreiro LG, Amendoeira MRR. *Toxoplasma gondii*: Epidemiological study of pigs from southwestern Paraná, Brazil. *Pesq Vet Bras* 2008; **28**: 15–18.
597. Miller AMA, Gardner IA, Packham A, Mazet JK, Hanni KD, Jessup D *et al.* Evaluation of an Indirect Fluorescent Antibody Test ( IFAT ) for Demonstration of Antibodies to *Toxoplasma gondii* in the Sea Otter (*Enhydra lutris*). *Journal of Parasitology* 2002; **88**: 594–599.
598. Miller MA, Miller WA, Conrad PA, James ER, Melli AC, Leutenegger CM *et al.* Type X *Toxoplasma gondii* in a wild mussel and terrestrial carnivores from coastal California: New linkages between terrestrial mammals, runoff and toxoplasmosis of sea otters. *International Journal for Parasitology* 2008; **38**: 1319–1328.
599. Minetto M, Witter R, de Oliveira A, Minetto J, Barros M, de Aguiar D *et al.* Antibodies anti-*Toxoplasma gondii* and anti-*Neospora caninum* in backyard pigs from the state of Mato Grosso, Brazil. *Revista Brasileira de Parasitologia Veterinaria* 2019; **28**: 403–409.
600. Mir NA, Chabra MB, Bhardwaj RM, Gautam OP. Toxoplasma infection and some other protozoan parasites of the wild rat in India. *Indian Veterinary Journal* 1982; **59**: 60–63.
601. Mitchell MA, Hungerford LL, Nixon C, Esker T, Sullivan J, Koerkenmeier R *et al.* Serologic survey for selected infectious disease agents in raccoons from Illinois. *Journal of Wildlife Diseases* 1999; **35**: 347–355.
602. Mitchell SM, Richardson DJ, Lindsay DS. Prevalence of Agglutinating Antibodies to *Toxoplasma gondii* in Striped Skunks (*Mephitis mephitis*), Opossums (*Didelphis virginiana*), and Raccoons (*Procyon lotor*) From Connecticut. *Journal of Parasitology* 2006; **92**: 664–665.
603. Miura A, de Barros L, Ferreira F, Neto J, Franco P, Su C *et al.* Genotyping of *Toxoplasma gondii* isolated from pigs for human consumption. *Parasitology Research* 2019; **118**: 1593–1599.
604. Molina CV, Catão-Dias JL, Neto JSF, Vasconcellos SA, Gennari SM, Valle RDR do *et al.* Sero-epidemiological survey for brucellosis, leptospirosis, and toxoplasmosis in free-ranging *Alouatta caraya* and *Callithrix penicillata* from São Paulo State, Brazil. *Journal of Medical Primatology* 2014; **43**: 197–201.
605. Molina CV, Krawczak F da S, Bueno MG, Soares HS, Genari SM, Pissinatti A *et al.* Negative serosurvey of *Toxoplasma gondii* antibodies in Golden-headed Lion Tamarin (*Leontopithecus chrysomelas*) from Niterói/RJ, Brazil. *Rev Bras Parasitol Vet* 2017; **26**: 115–118.
606. Moraes É, Faria E, Batista A, Freitas A, Silva J, Albuquerque P *et al.* *Toxoplasma gondii* detection in the semen of naturally infected sheep. *Pesquisa Veterinaria Brasileira* 2010; **30**: 915–917.
607. Moraes L, Raimundo J, Guimaraes A, Santos H, Macedo G, Massard C *et al.* Occurrence of anti-*Neospora caninum* and anti-*Toxoplasma gondii* IgG antibodies in goats and sheep in western Maranhao, Brazil. *Revista Brasileira de Parasitologia Veterinaria* 2011; **20**: 312–317.
608. Morariu S, Bulacu A, Morariu F, Hăgătiș A, Dărăbuș G, Suici T *Et Al.* Parasites – An Important Obstacle In Acclimatization and Conservation of The European Bison (*Bison bonasus bonasus*) In Romanian Armeniș-Plopu Reserve. 2021.
609. Morikawa V, Zimpel C, Paploski I, Lara M, Villalobos E, Romaldini A *et al.* Occurrences of anti-*Toxoplasma gondii* and anti-*Neospora caninum* antibodies in Barbary sheep at Curitiba zoo, southern Brazil. *Revista Brasileira de Parasitologia Veterinaria* 2014; **23**: 255–259.
610. Morrondo MP, Pérez-Creo A, Prieto A, Cabanelas E, Díaz-Cao JM, Arias MS *et al.* Prevalence and distribution of infectious and parasitic agents in roe deer from Spain and their possible role as reservoirs. *Italian Journal of Animal Science* 2017; **16**: 266–274.
611. Moskwa B, Kornacka A, Cybulska A, Cabaj W, Reiterova K, Bogdaszewski M *et al.* Seroprevalence of *Toxoplasma gondii* and *Neospora caninum* infection in sheep, goats, and fallow deer farmed on the same area. *Journal of Animal Science* 2018; **96**: 2468–2473.
612. Moura AB de, Osaki SC, Zulpo DL, Marana ERM. Occurrence of anti-*Toxoplasma gondii* antibodies in swine and ovine slaughtered at municipality of Guarapuava in the State of Paraná, Brazil. *Rev Bras Parasitol Vet* 2007; **16**: 54–56.
613. Movassaghi AR, Rassouli M, Fazaeli A, Salimi-Bejestani MR. Outbreak of ovine congenital toxoplasmosis in Iran, confirmed by different diagnostic methods. *Journal of Parasitic Diseases : official organ of the Indian Society for Parasitology* 2016; **40**: 152–6.
614. Muchina KD, Muturi KS, John K, Maina N, John M. Physico-Clinical and Haematological Changes in Olive Baboon (*Papio anubis*) Model of Latent Toxoplasmosis and Toxoplasmic Encephalitis. *International Journal of Primatology Research* 2020; **3**: 001–009.
615. Mucker EM, Dubey JP, Lovallo MJ, Humphreys JG. Seroprevalence of antibodies to *Toxoplasma gondii* in the Pennsylvania Bobcat (*Lynx rufus rufus*). *Journal of Wildlife Diseases* 2006; **42**: 188–191.
616. Muñoz-Zanzi C, Tamayo R, Balboa J, Hill D. Detection of Oocyst-Associated Toxoplasmosis in Swine from Southern Chile. *Zoonoses and Public Health* 2012; **59**: 389–392.
617. Muradian V, Ferreira LR, Lopes EG, de Oliveira Esmerini P, de Jesus Pena HF, Soares RM *et al.* A Survey of *Neospora caninum* and *Toxoplasma gondii* Infection in Urban Rodents from Brazil. *Journal of Parasitology* 2012; **98**: 128–134.
618. Muraro LS, Caramori Júnior JG, Amendoeira MRR, Pereira JA, Oliveira Filho JX de, Vicente RT *et al.* Seroprevalence of *Toxoplasma gondii* infection in swine matrices in Nova Mutum and Diamantino, Mato Grosso, Brazil. *Rev Bras Parasitol Vet* 2010; **19**: 254–255.
619. Murata K, Mizuta K, Imazu K, Terasawa F, Taki M, Endoh T. The Prevalence of *Toxoplasma gondii* Antibodies in Wild and Captive Cetaceans From Japan. *Journal of Parasitology* 2004; **90**: 896–898.
620. Murphy TM, Walochnik J, Hassl A, Moriarty J, Mooney J, Toolan D *et al.* Study on the prevalence of *Toxoplasma gondii* and *Neospora caninum* and molecular evidence of *Encephalitozoon cuniculi* and *Encephalitozoon intestinalis* infections in red foxes (V*ulpes vulpes*) in rural Ireland. *Veterinary Parasitology* 2007. doi:10.1016/j.vetpar.2007.02.017.
621. Muz M, Altug N, Karakavuk M. Seroprevalence of *T. gondii* in dairy ruminant production systems, shepherd dogs among the herds and detection of *T. gondii*- like oocyst in cat feces in Hatay Region. *AVKAE Dergisi* 2013; **3**: 38–45.
622. Naidenko SV, Hernandez-Blanco JA, Seryodkin IV, Miquelle DG, Blidchenko EYu, Litvinov MN *et al.* Serum Prevalence of Bears in the Russian Far East to Different Pathogens. *Biol Bull Russ Acad Sci* 2019; **46**: 960–965.
623. Namroodi S, Shirazi AS, Khaleghi SR, Mills JN, Kheirabady V. Frequency of exposure of endangered Caspian seals to Canine distemper virus, *Leptospira interrogans,* and *Toxoplasma gondii*. *PLoS ONE* 2018; **13**: 1–11.
624. Namroodi S, Yousefi MR, Milanloo D. *Toxoplasma gondii* serosurvey in Golden Jackals from Golestan province, Iran. *International Journal of Molecular and Clinical Microbiology* 2014; **2**: 446–450.
625. Nardoni S, Angelici MC, Mugnaini L, Mancianti F. Prevalence of *Toxoplasma gondii* infection in *Myocastor coypus* in a protected Italian wetland. *Parasites & Vectors* 2011; **4**: 240.
626. Nascimento COM, Silva MLCR, Kim PCP, Gomes AAB, Gomes ALV, Maia RCC *et al.* Occurrence of *Neospora caninum* and *Toxoplasma gondii* DNA in brain tissue from hoary foxes (*Pseudalopex vetulus*) in Brazil. *Acta Tropica* 2015; **146**: 60–65.
627. Nematollahi A, Shahbazi P, Nosrati S. Serological survey of antibodies to *Toxoplasma gondii* in sheep in north-west of Iran. *International Journal of Advanced Biological and Biomedical Research* 2014; **2**: 2605–2608.
628. Nezval J, Literák I. *Toxoplasma gondii* in muskrat (*Ondatra zibethicus*). *Vet Med (Praha)* 1994; **39**: 743–746.
629. Nicholas B, Ravel A, Leighton P, Stephen C, Iqbal A, Ndao M *et al.* Foxes (*Vulpes vulpes*) as sentinels for parasitic zoonoses, *Toxoplasma gondii* and *Trichinella nativa*, in the northeastern Canadian Arctic. *International Journal for Parasitology: Parasites and Wildlife* 2018; **7**: 391–397.
630. Nicholson KL, Noon TH, Krausman PR. Serosurvey of mountain lions in southern Arizona. *Wildlife Society Bulletin* 2012; **36**: 615–620.
631. Niedringhaus KD, Brown JD, Ternent MA, Cleveland CA, Yabsley MJ. A Serosurvey of Multiple Pathogens in American Black Bears (*Ursus americanus*) in Pennsylvania, USA Indicates a Lack of Association with Sarcoptic Mange. *Veterinary Sciences* 2019; **6**: 75.
632. Nogami S, Moritomo T, Hayashi Y. Prevalence of Anti-*Toxoplasma gondii* Antibody in Wild Boar, Sus scrofa riukiuanus, on Iriomote Island, Japan - ProQuest. *Veterinary Research Communications* 1999; **23**: 211–214.
633. Nunes A, da Silva E, de Oliveira J, Yamasaki E, Kim P, de Almeida J *et al.* Application of different techniques to detect *Toxoplasma gondii* in slaughtered sheep for human consumption. *Revista Brasileira de Parasitologia Veterinaria* 2015; **24**: 416–421.
634. Núñez-Egido S, Lowther A, Nymo IH, Klein J, Breines EM, Tryland M. Pathogen surveillance in Southern Ocean pinnipeds. *Polar Research* 2020. doi:10.33265/polar.v39.3841.
635. Nutter FB, Levine JF, Stoskopf MK, Gamble HR, Dubey JP. Seroprevalence of *Toxoplasma gondii* and Trichinella spiralis in North Carolina black bears (*Ursus americanus*). *Journal of Parasitology* 1998; **84**: 1048–1050.
636. O’Crowley K, Wilson JG. Feral mink (*Mustela vison)* and their potential as disease vectors in Ireland - an investigation in co Wicklow. *Irish Veterinary Journal* 1991; **44**: 71–74.
637. Obendorf DL, Statham P, Driessen M. Detection of agglutinating antibodies to *Toxoplasma gondii* in sera from free-ranging eastern barred bandicoots (*Perameles gunnii*). *Journal of Wildlife Diseases* 1996; **32**: 623–626.
638. Oertley KD, Walls KW. Prevalence of antibodies to *Toxoplasma gondii* amoung bobcats of West Virginia and Georgia. *Journal of the American Veterinary Medical Association* 1980; **177**: 852–853.
639. Oh J, Lee S, Lee S, Kim Y, Park S, Rhee M *et al.* Detection of Antibodies against *Toxoplasma gondii* in Cattle Raised in Gyeongbuk Province, Korea. *Journal Of Food Protection* 2016; **79**: 821–824.
640. Oksanen A, Åsbakk K, Prestrud KW, Aars J, Derocher AE, Tryland M *et al.* Prevalence of Antibodies Against *Toxoplasma gondii* in Polar Bears (*Ursus maritimus*) From Svalbard and East Greenland. *Journal of Parasitology* 2009; **95**: 89–94.
641. Oksanen A, Lindgren E. Seroprevalence of toxoplasmosis in Finnish lynx (*Felis lynx*). In: *Proceedings of the 15th international conference of the World Association for the Advancement of Veterinary Parasitology. The World Association for the Advancement of Veterinary Parasitology*. 1995, p 88.
642. Oksanen A, Tryland M, Johnsen K, Dubey JP. Serosurvey of *Toxoplasma gondii* in North Atlantic marine mammals by the use of agglutination test employing whole tachyzoites and dithiothreitol. *Comparative Immunology, Microbiology and Infectious Diseases* 1998; **21**: 107–114.
643. Oksanen A, Åsbakk K, Nieminen M, Norberg H, Näreaho A. Antibodies against *Toxoplasma gondii* in Fennoscandian reindeer - Association with the degree of domestication. *Parasitology International* 1997; **46**: 255–261.
644. Oliveira GC, de Souza Almeida HM, Sartori RS, Rossi GAM, de Oliveira LG, Langoni H. Prevalence of *Toxoplasma gondii* infections in swine of non-tecnified rearing farms of the northeastern region of the state of São Paulo, Brazil and associated risk factors. *Parasite Epidemiology and Control* 2019; **4**: e00080.
645. Oliveira S, Mattos PSR, Mattos KK, Toppa RH, Costa AP, Marcili A *et al.* Presence of anti-*Toxoplasma gondii*, -*Neospora caninum*, -Leishmania spp. and -*Ehrlichia canis* antibodies in free-ranging maned wolves (*Chrysocyon brachyurus*) in the northeastern region of the state of São Paulo, Brazil. *Brazilian Journal of Veterinary Research and Animal Science* 2016; **53**: 243–250.
646. Omata Y, Hammond T, Itoh K, Murata K. Antibodies against *Toxoplasma gondii* in the Pacific Bottlenose Dolphin (*Tursiops aduncus)* from the Solomon Islands. *Journal of Parasitology* 2005; **91**: 965–967.
647. Omata Y, Ishiguro N, Kano R, Masukata Y, Kudo A, Kamiya H *et al.* Prevalence of *Toxoplasma gondii* and *Neospora caninum* in Sika Deer from Eastern Hokkaido, Japan. *Journal of Wildlife Diseases* 2005; **41**: 454–458.
648. Onuma SSM, Melo ALT, Kantek DLZ, Crawshaw-Junior PG, Morato RG, May-Júnior JA *et al.* Exposure of free-living jaguars to *Toxoplasma gondii*, *Neospora caninum* and *Sarcocystis neurona* in the Brazilian Pantanal. *Brazilian Journal of Veterinary Parasitology* 2014; **23**: 547–553.
649. Onyiche TE, Ademola IO. Seroprevalence of anti-*Toxoplasma gondii* antibodies in cattle and pigs in Ibadan, Nigeria. *Journal of Parasitic Diseases : official organ of the Indian Society for Parasitology* 2015; **39**: 309–14.
650. Opsteegh M, Spano F, Aubert D, Balea A, Burrells A, Cherchi S *et al.* The relationship between the presence of antibodies and direct detection of *Toxoplasma gondii* in slaughtered calves and cattle in four European countries. *International Journal For Parasitology* 2019; **49**: 515–522.
651. Opsteegh M, Teunis P, Mensink M, Züchner L, Titilincu A, Langelaar M *et al.* Evaluation of ELISA test characteristics and estimation of *Toxoplasma gondii* seroprevalence in Dutch sheep using mixture models. *Preventative Veterinary Medicine* 2010; **96**: 232–240.
652. Opsteegh M, Swart A, Fonville M, Dekkers L, Giessen J van der. Age-Related *Toxoplasma gondii* Seroprevalence in Dutch Wild Boar Inconsistent with Lifelong Persistence of Antibodies. *PLOS ONE* 2011; **6**: e16240.
653. Opsteegh, M., Teunis, P., Züchner, L., Koets, A., Langelaar, M., & van der Giessen, J. (2011). Low predictive value of seroprevalence of Toxoplasma gondii in cattle for detection of parasite DNA. *International Journal For Parasitology*, *41*(3–4), 343–354.
654. Ortega-Pacheco A, Viana K, Guzmán-Marín E, Segura-Correa J, Alvarez-Fleites M, Jimenez-Coello A. Prevalence and Risk Factors of *Toxoplasma gondii* in Fattening Pigs Farm from Yucatan, Mexico. *Biomed Research International* 2013; **2013**. doi:10.1155/2013/231497.
655. Ozmutlu Cak D, Karatepe B. Seroprevalence of *Toxoplasma gondii* in Sheep from Nevşehir Province in Turkey. *TurkiyeParazitolDerg* 2017; **41**: 148–151.
656. Pablos-Tanarro A, Ortega-Mora LM, Palomo A, Casasola F, Ferre I. Seroprevalence of *Toxoplasma gondii* in Iberian pig sows. *Parasitol Res* 2018; **117**: 1419–1424.
657. Padilha TC, Zitelli LC, Webster A, Dall’Agnol B, Rosa VB da, Souza U *et al.* Serosurvey of antibodies against zoonotic pathogens in free-ranging wild canids (*Cerdocyon thous* and *Lycalopex gymnocercus*) from Southern Brazil. *Comparative Immunology, Microbiology and Infectious Diseases* 2021; **79**: 101716.
658. Pagmadulam B, Myagmarsuren P, Yokoyama N, Battsetseg B, Nishikawa Y. Seroepidemiological study of *Toxoplasma gondii* in small ruminants (sheep and goat) in different provinces of Mongolia. *Parasitology International* 2020; **74**. doi:10.1016/j.parint.2019.101996.
659. Pagmadulam B, Myagmarsuren P, Fereig RM, Igarashi M, Yokoyama N, Battsetseg B *et al.* Seroprevalence of *Toxoplasma gondii* and *Neospora caninum* infections in cattle in Mongolia. *Veterinary Parasitology: Regional Studies and Reports* 2018; **14**: 11–17.
660. Panadero R, Painceira A, López C, Vázquez L, Paz A, Díaz P *et al.* Seroprevalence of *Toxoplasma gondii* and *Neospora caninum* in wild and domestic ruminants sharing pastures in Galicia (Northwest Spain). *Research in Veterinary Science* 2010; **88**: 111–115.
661. Pandey VS, Van Knapen F. The seroprevalence of toxoplasmosis in sheep, goats and pigs in Zimbabwe. *Annals of Tropical Medicine & Parasitology* 1992; **86**: 313–315.
662. Papatsiros V, Athanasiou L, Stougiou D, Papadopoulos E, Maragkakis G, Katsoulos P *et al.* Cross-Sectional Serosurvey and Risk Factors Associated with the Presence of *Toxoplasma gondii* Antibodies in Pigs in Greece. *Vector-borne and zoonotic diseases* 2016; **16**: 48–53.
663. Papini R, Di Ciccio P, Marangi M, Ghidini S, Zanardi E, Vergara A *et al.* Occurrence of *Toxoplasma gondii* in Carcasses of Pigs Reared in Intensive Systems in Northern Italy. *Journal of Food Protection* 2017; **80**: 515–522.
664. Parameswaran N, O’Handley RM, Grigg ME, Fenwick SG, Thompson RCA. Seroprevalence of *Toxoplasma gondii* in wild kangaroos using an ELISA. *Parasitology International* 2009; **58**: 161–165.
665. Pardini L, Maksimov P, Herrmann DC, Bacigalupe D, Rambeaud M, Machuca M *et al.* Evaluation of an in-house TgSAG1 (P30) IgG ELISA for diagnosis of naturally acquired *Toxoplasma gondii* infection in pigs. *Veterinary Parasitology* 2012; **189**: 204–210.
666. Pas A, Dubey JP. Seroprevalence of Antibodies to *Toxoplasma gondii* in Gordon’s Wildcat (*Felis silvestris gordoni*) in the Middle East. *Journal of Parasitology* 2008; **94**: 1169–1169.
667. Paştiu AI, Cozma-Petruț A, Mercier A, Balea A, Galal L, Mircean V *et al.* Prevalence and genetic characterization of *Toxoplasma gondii* in naturally infected backyard pigs intended for familial consumption in Romania. *Parasites & Vectors* 2019; **12**: 586.
668. Paul-Murphy J, Work T, Hunter D, McFie E, Fjelline D. Serologic survey and serum biochemical reference ranges of the free-ranging mountain lion (*Felis concolor*) in California. *Journal of Wildlife Diseases* 1994; **30**: 205–215.
669. Pavlova EV, Kirilyuk EV, Naidenko SV. Occurrence Pattern of Influenza A Virus, *Coxiella burnetii*, *Toxoplasma gondii*, and *Trichinella* sp. in the Pallas Cat and Domestic Cat and Their Potential Prey Under Arid Climate Conditions. *Arid Ecosystems* 2016; **6**: 277–283.
670. Penzhorn BL, Stylianides E, Vuuren MV, Alexander K. Short communications Seroprevalence of *Toxoplasma gondii* in free-ranging lion and in southern Africa. *South African Journal of Wildlife Research* 2002; **32**: 163–165.
671. Pereira, D. C., Dubey, J. P., Da Mata, A., Neto, H., Cardoso, L., & Lopes, A. P. (2020). Seroepidemiology of *Toxoplasma gondii* in domestic cattle, sheep, goats and pigs from São Tomé and Príncipe. *Revista Brasileira De Parasitologia Veterinaria = Brazilian Journal of Veterinary Parasitology: Orgao Oficial Do Colegio Brasileiro De Parasitologia Veterinaria*, *29*(1), e014819.
672. Pereira M, Peixoto R, Langoni H, Greca H, de Azevedo S, Porto W *et al.* Risk factors for *Toxoplasma gondii* infection in sheep and goats in Pernambuco, Brazil. *Pesquisa Veterinaria Brasileira* 2012; **32**: 140–146.
673. Pérez-Grisales LJ, Cruz-Moncada M, Peláez-Sánchez R, Díaz-Nieto JF. *Toxoplasma gondii* infection in Colombia with a review of hosts and their ecogeographic distribution. *Zoonoses Public Health* 2021; **68**: 38–53.
674. Pezerico GB, Pezerico SB, Silva RC, Hoffmann JL, Camargo LB, Langoni H. Occurrence of Antibodies Anti-*Toxoplasma gondii* and Anti-*Leptospira* Spp. In Swine Slaughtered In Three Slaughterhouses of Minas Gerais and São Paulo States, Brazil. *Arq Inst Biol* 2022; **74**: 267–270.
675. Philippa JDW, Leighton FA, Daoust PY, Nielsen O, Pagliarulo M, Schwantje H *et al.* Antibodies to selected pathogens in free-ranging terrestrial carnivores and marine mammals in Canada. *Veterinary Record* 2004; **155**: 135–143.
676. Piassa FR, Araújo JB de, Rosa RC da, Mattei RJ, Silva RC da, Langoni H *et al.* Prevalence and risk factors for *Toxoplasma gondii* infection in certified and non-certified pig breeding farms in the Toledo microregion, PR, Brazil. *Rev Bras Parasitol Vet* 2010; **19**: 152–156.
677. Pilfold NW, Richardson ES, Ellis J, Jenkins E, Scandrett WB, Hernández-Ortiz A *et al.* Long-term increases in pathogen seroprevalence in polar bears (*Ursus maritimus*) influenced by climate change. *Global Change Biology* 2021; **27**: 4481–4497.
678. Pinheiro J, Mota R, Oliveira A, Faria E, Gondim L, da Silva A *et al.* Prevalence and risk factors associated to infection by *Toxoplasma gondii* in ovine in the State of Alagoas, Brazil. *Parasitology Research* 2009; **105**: 709–715.
679. Pipia A, Varcasia A, Dessì G, Panzalis R, Gai C, Nonnis F *et al.* Seroepidemiological and biomolecular survey on *Toxoplasma gondii* infection on organic pig farms. *Parasitology Research* 2018; **117**: 1637–1641.
680. Poljak Z, Dewey CE, Friendship RM, Martin SW, Christensen J, Ojkic D *et al.* Pig and herd level prevalence of *Toxoplasma gondii* in Ontario finisher pigs in 2001, 2003, and 2004. *Can J Vet Res* 2008; **72**: 303–310.
681. Pomerantz J, Rasambainarivo FT, Dollar L, Rahajanirina LP, andrianaivoarivelo R, Parker P *et al.* Prevalence of antibodies to selected viruses and parasites in introduced and endemic carnivores in western Madagascar. *Journal of Wildlife Diseases* 2016; **52**: 544–552.
682. Portas TJ, Evans MJ, Spratt D, Vaz PK, Devlin JM, Barbosa AD *et al.* Baseline Health and Disease Assessment of Founder Eastern Quolls (*Dasyurus viverrinus*) During a Conservation Translocation To Mainland Australia. *Journal of Wildlife Diseases* 2020; **56**: 547–559.
683. Poulsen A, Fritz H, Clifford DL, Conrad PA, Roy A, Glueckert E *et al.* Prevalence and potential impact of *Toxoplasma gondii* on the endangered Amargosa vole (*Microtus californicus scirpensis*), California, USA. *Journal of Wildlife Diseases* 2017; **53**: 62–72.
684. Powell L, Cheney T, Williamson S, Guy E, Smith R, Davies R *et al.* A prevalence study of Salmonella spp., Yersinia spp., *Toxoplasma gondii* and porcine reproductive and respiratory syndrome virus in UK pigs at slaughter. *Epidemiology and Infection* 2016; **144**: 1538–1549.
685. Prestrud KW, Åsbakk K, Fuglei E, Mørk T, Stien A, Ropstad E *et al.* Serosurvey for *Toxoplasma gondii* in arctic foxes and possible sources of infection in the high Arctic of Svalbard. *Veterinary Parasitology* 2007; **150**: 6–12.
686. Prestrud KW, Dubey JP, Åsbakk K, Fuglei E, Su C. First isolate of *Toxoplasma gondii* from arctic fox (*Vulpes lagopus*) from Svalbard. *Veterinary Parasitology* 2008; **151**: 110–114.
687. Proença LM, Silva JCR, Galera PD, Lion MB, Marinho-Filho JS, Ragozo AMA *et al.* Serologic Survey of Infectious Diseases in Populations of Maned Wolf (*Chrysocyon brachyurus* ) and Crab-Eating Fox (*Cerdocyon thous*) From Águas Emendadas Ecological Station, Brazil. *Journal of Zoo and Wildlife Medicine* 2013; **44**: 152–155.
688. Puvanesuaran, V. R., Noordin, R., & Balakrishnan, V. (2013). Genotyping of *Toxoplasma gondii* Isolates from Wild Boars in Peninsular Malaysia. *PLOS ONE*, *8*(4), e61730.
689. Qi Y, Zhang D, Mengke L. Serological investigation of several animal diseases in large scale livestock farms in Dulan County, Qinghai Province. *Chinese Qinghai Journal of Animal and Veterinary Science* 2018; **48**: 49–52.
690. Qin S-Y, Cong W, Liu Y, Li N, Wang Z-D, Zhang F-K *et al.* Molecular detection and genotypic characterization of *Toxoplasma gondii* infection in bats in four provinces of China. *Parasites & Vectors* 2014; **7**: 558.
691. Qiu J, Wang C, Zhang X, Sheng Z, Chang Q, Zhao Q *et al.* Seroprevalence of *Toxoplasma gondii* in Beef Cattle and Dairy Cattle in Northeast China. *Foodborne Pathogens and Disease* 2012; **9**: 579–582.
692. Quinn PJ, Ramsden ROR, Johnston DHD. Toxoplasmosis: A serological survey in Ontario wildlife. *Journal of Wildlife Diseases* 1976; **12**: 504–510.
693. Račka K, Bártová E, Budíková M, Vodrážka P. Survey of *Toxoplasma gondii* antibodies in meat juice of wild boar *(Sus scrofa)* in several districts of the Czech Republic. *Annals of Agricultural and Environmental Medicine* 2015; **22**: 231–235.
694. Raeghi S, Akaberi A, Sedeghi S. Seroprevalence of *Toxoplasma gondii* in Sheep, Cattle and Horses in Urmia North-West of Iran. *Iranian Journal of Parasitology* 2011; **6**: 90–94.
695. Rah H, Chomel BB, Follmann EH, Kasten RW, Hew CH, Farver TB *et al.* Serosurvey of selected zoonotic agents in polar bears (*Ursus maritimus*). *Veterinary Record* 2005; **156**: 7–13.
696. Rahman M, Azad M, Nahar L, Rouf S, Ohya K, Chiou S *et al.* Age-Specificity of *Toxoplasma gondii* Seroprevalence in Sheep, Goats and Cattle on Subsistence Farms in Bangladesh. *Journal of Veterinary Medical Science* 2014; **76**: 1257–1259.
697. Rainwater KL, Marchese K, Slavinski S, Humberg LA, Dubovi EJ, Jarvis JA *et al.* Health survey of free-ranging raccoons (*Procyon lotor*) in Central Park, New York, New York, USA: Implications for human and domestic animal health. *Journal of Wildlife Diseases* 2017; **53**: 272–284.
698. Rajamanickam C, Cheah TS, Paramasvaran S. Antibodies to *Toxoplasma gondii* from domestic animals in Malaysia. *Tropical Animal Health and Production* 1990; **22**: 61–62.
699. Ramey AM, Cleveland CA, Hilderbrand GV, Joly K, Gustine DD, Mangipane B *et al.* Exposure of Alaska brown bears (*Ursus arctos*) to bacterial, viral and parasitic agents varies spatiotemporally and may be influenced by age. *Journal of Wildlife Diseases* 2019; **55**: 576–588.
700. Ramzan M, Akhtar M, Muhammad F, Hussain I, Hiszczynska-Sawicka E, Haq A *et al.* Seroprevalence of *Toxoplasma gondii* in sheep and goats in Rahim Yar Khan (Punjab), Pakistan. *Tropical Animal Health and Production* 2009; **41**: 1225–1229.
701. Randel CJ III, Vanherweg WJ. Survey for Select Pathogens in the Desert Kit Fox (*Vulpes macrotis arsipus*) in California, USA. *Journal of Wildlife Diseases* 2022; **58**: 631–635.
702. Ranucci D, Veronesi F, Moretti A, Branciari R, Miraglia D, Manfredi MT *et al.* Seroprevalence of *Toxoplasma gondii* in wild boars *(Sus scrofa)* from Central Italy. *Parasite* 2013; **20**: 4–7.
703. Rasambainarivo F, andriamihajarivo MN, Dubovi E, Parker PG. Patterns of exposure of carnivores to selected pathogens in the Betampona Natural Reserve Landscape, Madagascar. *Journal of Wildlife Diseases* 2018; **54**: 386–391.
704. Razmi G, Barati M. Prevalence of *Neospora caninum* and *Toxoplasma gondii* Antibodies in Bulk Milk of Dairy Cattle, Mashhad, Iran. *Archives of Razi Institute* 2017; **72**: 265–269.
705. Rêgo W, Paula N, Vitor R, Silva R, Diniz B, Sousa M *et al.* Risk factors for *Toxoplasma gondii* infection in goats and sheep raised in the State of Piaui in northeast Brazil. *Small Ruminant Research* 2016; **141**: 17–23.
706. Reichard MV, Torretti L, Garvon JM, Dubey JP. Prevalence of Antibodies to *Toxoplasma gondii* in Wolverines From Nunavut, Canada. *Journal of Parasitology* 2008; **94**: 764–765.
707. Reiling SJ, Measures L, Feng S, Boone R, Merks H, Dixon BR. *Toxoplasma gondii*, Sarcocystis sp. and *Neospora caninum*-like parasites in seals from northern and eastern Canada: potential risk to consumers. *Food and Waterborne Parasitology* 2019; **17**: e00067.
708. Reiterová K, Špilovská S, Čobádiová A, Hurníková Z. Prevalence of *Toxoplasma gondii* and *Neospora caninum* in red foxes in Slovakia. *Acta Parasitologica* 2016; **61**. doi:10.1515/ap-2016-0105.
709. Rendón-Franco E. Frequency of Antibodies Against *Toxoplasma gondii* in Wild Carnivores and Marsupials in Northeast Mexico. *Neotropical Helminthology* 2014; **8**.
710. Rendón-Franco E, Caso-Aguilar A, Jiménez-Sánchez NG, Hernandez-Jauregui DMB, Sandoval-Sánchez AL, Zepeda-López HM. Prevalence of anti-*Toxoplasma gondii* antibody in free-ranging ocelots (*Leopardus pardalis*) from Tamaulipas, Mexico. *Journal of Wildlife Diseases* 2012; **48**: 829–831.
711. Rendón-Franco E, Xicoténcatl-García L, Rico-Torres CP, Muñoz-García CI, Caso-Aguilar A, Suzán G *et al.* Toxoplasmosis seroprevalence in wild small rodents, potentially preys of ocelots in north-eastern Mexico. *Parasite* 2014; **21**. doi:10.1051/parasite/2014058.
712. Rengifo-Herrera C, Ortega-Mora LM, Álvarez-García G, Gómez-Bautista M, García-Párraga D, García-Peña FJ *et al.* Detection of *Toxoplasma gondii* antibodies in Antarctic pinnipeds. *Veterinary Parasitology* 2012; **190**: 259–262.
713. Reperant LA, Hegglin D, Tanner I, Fischer C, Deplazes P. Rodents as shared indicators for zoonotic parasites of carnivores in urban environments. *Parasitology* 2009; **136**: 329–337.
714. Ribas MP, Almería S, Fernández-Aguilar X, Pedro GD, Lizarraga P, Alarcia-Alejos O *et al.* Tracking *Toxoplasma gondii* in freshwater ecosystems: interaction with the invasive American mink (*Neovison vison*) in Spain. *Parasitology Research* 2018; **117**: 2275–2281.
715. Richini-Pereira VB, Marson PM, Silva RC da, Langoni H. Genotyping of *Toxoplasma gondii* and *Sarcocystis* spp. in road-killed wild mammals from the Central Western Region of the State of São Paulo, Brazil. *Rev Soc Bras Med Trop* 2016; **49**: 602–607.
716. Richomme C, Afonso E, Tolon V, Ducrot C, Halos L, Alliot A *et al.* Seroprevalence and factors associated with *Toxoplasma gondii* infection in wild boar *(Sus scrofa)* in a Mediterranean island. *Epidemiology and Infection* 2010; **138**: 1257–1266.
717. Richomme C, Aubert D, Gilot-Fromont E, Ajzenberg D, Mercier A, Ducrot C *et al.* Genetic characterization of *Toxoplasma gondii* from wild boar *(Sus scrofa)* in France. *Veterinary Parasitology* 2009; **164**: 296–300.
718. Riemann HP, Ruppanner R, Franti CE, Behymer DE. Toxoplasma antibodies among bobcats and other carnivores of northern California. *Journal of Wildlife Diseases* 1975; **11**: 272–276.
719. Riemann HP, Thompson RA, Behymer DE, Ruppanner R. Toxoplasmosis and Q Fever Antibodies among Wild Carnivores in California. *Journal of Wildlife Management* 1978; **42**: 198–202.
720. Riley SPD, Foley J, Chomel B. Exposure to feline and canine pathogens in bobcats and gray foxes in urban and rural zones of a national park in California. *Journal of Wildlife Diseases* 2004; **40**: 11–22.
721. Rizzo H, Villalobos E, Meira E, Marques E, Beraldi F, Gregory L. Occurrence of antibodies anti-*Toxoplasma gondii* and anti-*Neospora caninum* in sheep with history of reproductive disorders and risk factors. *Pesquisa Veterinaria Brasileira* 2018; **38**: 1317–1326.
722. Rocchigiani G, Nardoni S, D’Ascenzi C, Nicoloso S, Picciolli F, Papini RA *et al.* Seroprevalence of *Toxoplasma gondii* and *Neospora caninum* in red deer from central Italy. *Annals of Agricultural and Environmental Medicine* 2016; **23**: 699–701.
723. Rocha D, Moura R, Maciel B, Guimaraes L, O’dwyer H, Munhoz A *et al.* Detection of *Toxoplasma gondii* DNA in naturally infected sheep’s milk. *Genetics and Molecular Research* 2015; **14**: 8658–8662.
724. Roelke ME, Forrester DJ, Jacobson ER, Kollias GV, Scott FW, Barr MC *et al.* Seroprevalence of infectious disease agents in free-ranging Florida panthers (*Felis concolor coryi*). *Journal of Wildlife Diseases* 1993; **29**: 36–49.
725. Roelke ME, Johnson WE, Millan J, Palomares F, Revilla E, Rodriguez A *et al.* Exposure to disease agents in the endangered Iberian lynx (*Lynx pardinus)*. *European Journal of Wildlife Research* 2008; **54**: 171–178.
726. Roqueplo C, Halos L, Cabre O, Davoust B. *Toxoplasma gondii* In Wild and Domestic Animals From New Caledonia. *Parasite* 2011; **18**: 345–348.
727. Roqueplo C, Blaga R, Marié JL, Vallée I, Davoust B. Seroprevalence of *Toxoplasma gondii* in hunted wild boars *(Sus scrofa)* from southeastern France. *Folia Parasitologica* 2017; **64**: 19–21.
728. Rossi G, Cabral D, Ribeiro D, Pajuaba A, Corrêa R, Moreira R *et al.* Evaluation of *Toxoplasma gondii* and *Neospora caninum* infections in sheep from Uberlandia, Minas Gerais State, Brazil, by different serological methods. *Veterinary Parasitology* 2011; **175**: 252–259.
729. Rouatbi M, Amairia S, Lahmer M, Lassoued N, Rekik M, Wieland B *et al.* Detection of *Toxoplasma gondii* infection in semen of rams used for natural mating in commercial sheep farms in Tunisia. *Veterinary Parasitology- Regional Studies and Reports* 2019; **18**. doi:10.1016/j.vprsr.2019.100341.
730. Roug A, Doden E, Griffin T, Young J, Walden X, Norman N *et al.* Health Screening of American Beavers (*Castor canadensis*) in Utah, USA. *Journal of Wildlife Diseases* 2022; **58**: 902–908.
731. Ruiz-Fons F, Vicente J, Vidal D, Höfle U, Villanúa D, Gauss C *et al.* Seroprevalence of six reproductive pathogens in European wild boar *(Sus scrofa)* from Spain: The effect on wild boar female reproductive performance. *Theriogenology* 2006; **65**: 731–743.
732. Ruppanner R, Jessup DA, Ohishi I, Behymer DE, Franti CE. Serologic survey for certain zoonotic diseases in black bears in California. *Journal of the American Veterinary Medical Association* 1982; **181**: 1288–91.
733. Ryser-Degiorgis MP, Jakubek EB, Segerstad CH af, Bröjer C, Mörner T, Jansson DS *et al.* Serological survey of *Toxoplasma gondii* infection in free-ranging Eurasian Lynx (*Lynx lynx*) from Sweden. *Journal of Wildlife Diseases* 2006; **42**: 182–187.
734. Saadi N, Hussein A, Ewida R. Occurrence of *Toxoplasma gondii* in raw goat, sheep, and camel milk in Upper Egypt. *Veterinary World* 2018; **11**: 1262–1265.
735. Saavedra GM, Ortega YR. Seroprevalence of *Toxoplasma gondii* in Swine From Slaughterhouses in Lima, Peru, and Georgia, U.S.A. *Journal of Parasitology* 2004; **90**: 902–904.
736. Sadek OA, M. Abdel-Hameed Z, M. Kuraa H. Molecular Detection of *Toxoplasma gondii* DNA In Raw Goat and Sheep Milk With Discussion of Its Public Health Importance In Assiut Governorate. *Assiut Veterinary Medical Journal* 2015; **61**: 166–177.
737. Sah R, Talukder M, Rahman A, Alam M, Ward M. Seroprevalence of *Toxoplasma gondii* infection in ruminants in selected districts in Bangladesh. *Veterinary Parasitology- Regional Studies and Reports* 2018; **11**: 1–5.
738. Saito T, Kitamura Y, Tanaka E, Ishigami I, Taniguchi Y, Moribe J *et al.* Spatial distribution of anti-*Toxoplasma gondii* antibody-positive wild boars in Gifu Prefecture, Japan. *Sci Rep* 2021; **11**: 17207.
739. Sakata FBLS, Bellato V, Sartor AA, Moura AB de, Souza AP de, Farias JA. *Toxoplasma gondii* antibodies sheep in Lages, Santa Catarina, Brazil, and comparison using IFA and ELISA. *Rev Bras Parasitol Vet* 2012; **21**: 196–200.
740. Salcedo I, Fraschina J, Busch M, Guidobono JS, Unzaga JM, Dellarupe A *et al.* Role of *Mus musculus* in the transmission of several pathogens in poultry farms. *International Journal for Parasitology: Parasites and Wildlife* 2021; **14**: 130–136.
741. Samico Fernandes EFT, Samico Fernandes MFT, Kim PCP, de Albuquerque PPF, de Souza Neto OL, de S. Santos A *et al.* Prevalence of *Toxoplasma gondii* in Slaughtered Pigs in the State of Pernambuco, Brazil. *Journal of Parasitology* 2012; **98**: 690–691.
742. Samico-Fernandes E, de Melo R, Kim P, de Almeida J, de Barros L, Garcia J *et al.* First report of genotype #65 of *Toxoplasma gondii* in pigs. *Parasitology Research* 2015; **114**: 3927–3930.
743. Samico-Fernandes E, Samico-Fernandes M, de Albuquerque P, de Almeida J, Mota A, Neto O *et al.* *Toxoplasma gondii* in backyard pigs: seroepidemiology and mouse bioassay. *Acta Parasitologica* 2017; **62**: 466–470.
744. San Miguel JM, Gutiérrez-Expósito D, Aguado-Martínez A, Gonzá Lez-Zotes E, Pereira-Bueno J, Gómez-Bautista M *et al.* Effect of different ecosystems and management practices on *Toxoplasma gondii* and *Neospora caninum* infections in wild ruminants in Spain. *Journal of Wildlife Diseases* 2016; **52**: 293–300.
745. Sanati H, Fard S, Nahrevanian M, Khalili M, Safari Z. Seroprevalence of *Toxoplasma gondii* antibodies in dairy cows in Kerman Province, southeast Iran. *Current Research Journal of Biological Sciences* 2012; **4**: 417–421.
746. Sanders CW II, Olfenbuttel C, Pacifici K, Hess GR, Livingston RS, DePerno CS. Leptospira, Parvovirus, and Toxoplasma In The North American River Otter (*Lontra canadensis*) In North Carolina, USA. *Journal of Wildlife Diseases* 2020; **56**: 791–802.
747. Sandfoss M, dePerno C, Patton S, Flowers J, Kennedy-Stoskopf S. Prevalence of antibody to *Toxoplasma gondii* and Trichinella spp. in feral pigs *(Sus scrofa)* of Eastern North Carolina. *Journal of Wildlife Diseases* 2011; **47**: 338–343.
748. Santin API, Juliano RS, Silva AC, Oliveira VSF de, Junqueira-Kipnis AP, Brito Wmed De *Et Al.* Soroepidemiologia De *Neospora caninum* E *Toxoplasma gondii* Em Bovinos Da Raça Curraleiro. *Ciência Animal Brasileira / Brazilian Animal Science* 2017; **18**. doi:10.1590/cab18044649.
749. Santoro A, Tagel M, Must K, Laine M, Lassen B, Jokelainen P. *Toxoplasma gondii* seroprevalence in breeding pigs in Estonia. *Acta Veterinaria Scandinavica* 2017; **59**. doi:10.1186/s13028-017-0349-1.
750. Santoro A, Veronesi F, Milardi G, Ranucci D, Branciari R, Diaferia M *et al.* Sequence variation in the B1 gene among *Toxoplasma gondii* isolates from swine and cats in Italy. *Research in Veterinary Science* 2017; **115**: 353–355.
751. Santos L, Farias N, Oliveira P, Cademartori B, Ramos T, Oliveira F *et al.* Presence of *Toxoplasma gondii* infection in wild boar in southern Brazil. *Scholars Journal of Agriculture and Veterinary Sciences* 2016; **3**: 238–241.
752. Santos L, Damé M, Cademartori B, da Cunha N, Farias N, Ruas J. Occurrence of antibodies to *Toxoplasma gondii* in water buffaloes and meat cattle in Rio Grande do Sul State, southern Brazil. *Acta Parasitologica* 2013; **58**: 334–336.
753. Santos PS, Albuquerque GR, Silva VMF da, Martin AR, Marvulo MFV, Souza SLP *et al.* Seroprevalence of *Toxoplasma gondii* in free-living Amazon River dolphins (*Inia geoffrensis*) from central Amazon, Brazil. *Veterinary Parasitology* 2011; **183**: 171–173.
754. Santos SM, Mira A, Salgueiro PA, Costa P, Medinas D, Beja P. Avian trait-mediated vulnerability to road traffic collisions. *Biological Conservation* 2016; **200**: 122–130.
755. Santos TR dos, Nunes CM, Luvizotto MCR, Moura AB de, Lopes WDZ, Costa AJ da *et al.* Detection of *Toxoplasma gondii* oocysts in environmental samples from public schools. *Veterinary Parasitology* 2010; **171**: 53–57.
756. Santos T, Costa A, Toniollo G, Luvizotto M, Benetti A, Santos R *et al.* Prevalence of anti-*Toxoplasma gondii* antibodies in dairy cattle, dogs, and humans from the Jauru micro-region, Mato Grosso state, Brazil. *Veterinary Parasitology* 2009; **161**: 324–326.
757. Sarkari B, Yaghoobi K, Mansouri M, Asgari Q, Khabisi SA. Seroprevalence and genotyping of *Toxoplasma gondii* in wild boars *(Sus scrofa)* from southwestern Iran. *Jundishapur Journal of Microbiology* 2017; **10**: 1–6.
758. Satbige AS, Vijaya Bharathi M, Ganesan PI, Sreekumar C, Rajendran C. Detection of *Toxoplasma gondii* in small ruminants in Chennai using PCR and modified direct agglutination test. *Journal of Parasitic Diseases : official organ of the Indian Society for Parasitology* 2016; **40**: 1466–1469.
759. Sato S, Kabeya H, Makino T, Suzuki K, Asano M, Inoue S *et al.* Seroprevalence of *Toxoplasma gondii* Infection in Feral Raccoons (*Procyon lotor*) in Japan. *Journal of Parasitology* 2011; **97**: 956–957.
760. Schaefer JJ, Kirchgessner MS, Whipps CM, Mohammed HO, Bunting EM, Wade SE. Prevalence of antibodies to *Toxoplasma gondii* in white-tailed deer (*Odocoileus virginianus*) in New York state, USA. *Journal of Wildlife Diseases* 2013; **49**: 940–945.
761. Schoonman L, Wilsmore T, Swai E. Sero-epidemiological investigation of bovine toxoplasmosis in traditional and smallholder cattle production systems of Tanga Region, Tanzania. *Tropical Animal Health and Production*; **42**: 579–587.
762. Schowalter DB, Iveksen O, Coknek J, Gunson JK. Prevalence of antibodies to *Toxoplasma gondii* in striped skunks from Saskatchewan and Alberta. *Journal of Wildlife Diseases* 1980; **16**: 189–194.
763. Scimeca RC, Perez E, Fairbanks WS, Ammar S, Su C, Gerhold RW *et al.* Seroprevalence, DNA isolation, and genetic characterization of *Toxoplasma gondii* from black bear (*Ursus americanus*) sera collected in Eastern Oklahoma. *Parasitol Res* 2020; **119**: 1109–1115.
764. Scotter SE, Tryland M, Nymo IH, Hanssen L, Harju M, Lydersen C *et al.* Contaminants in Atlantic walruses in Svalbard part 1: Relationships between exposure, diet and pathogen prevalence. *Environmental Pollution* 2019; **244**: 9–18.
765. Sechi P, Ciampelli A, Cambiotti V, Veronesi F, Cenci-Goga B. Seroepidemiological study of toxoplasmosis in sheep in rural areas of the Grosseto district, Tuscany, Italy. *Italian Journal of Animal Science* 2013; **12**. doi:10.4081/ijas.2013.e39.
766. Seltmann A, Schares G, Aschenborn OHK, Heinrich SK, Thalwitzer S, Wachter B *et al.* Species-specific differences in *Toxoplasma gondii*, *Neospora caninum* and *Besnoitia besnoiti* seroprevalence in Namibian wildlife. *Parasites & Vectors* 2020; **13**: 7.
767. Sepúlveda MA, Muñoz-Zanzi C, Rosenfeld C, Jara R, Pelican KM, Hill D. *Toxoplasma gondii* in feral American minks at the Maullín river, Chile. *Veterinary Parasitology* 2011; **175**: 60–65.
768. Serieys LEK, Hammond-Aryee K, Bishop J, Broadfield J, O’Riain MJ, Helden PD van. High seroprevalence of *Toxoplasma gondii* in an urban caracal (*Caracal caracal*) population in South Africa. *Journal of Wildlife Diseases* 2019; **55**: 951–953.
769. Sgroi G, Viscardi M, Santoro M, Borriello G, D’Alessio N, Boccia F *et al.* Genotyping of *Toxoplasma gondii* in wild boar *(Sus scrofa)* in southern Italy: Epidemiological survey and associated risk for consumers. *Zoonoses Public Health* 2020; **67**: 803–811.
770. Shah M, Zahid M, Asmat P, Kaudar A, Jan A. Seroprevalence of *Toxoplasma gondii* infection in domestic animals of Mohmand agency, Pakistan. *JCLM* 2013; **1**: 70–73.
771. Shahiduzzaman M, Islam M, Khatun M, Batanova T, Kitoh K, Takashima Y. *Toxoplasma gondii* Seroprevalence in Domestic Animals and Humans in Mymensingh District, Bangladesh. *Journal Of Veterinary Medical Science* 2011; **73**: 1375–1376.
772. Shao J. Serological investigation of swine Toxoplasma disease in Xiamen. *Fujian Anim Sci Vet Med* 2017; **39**: 12–13.
773. Shao X, Yuan L, Zhai K, Qian W, Yan W, Wang T. Seroprevalence and PCR detection of *Toxoplasma gondii* in pigs in Luoyang. *Heilongjiang Anim Sci Vet Med* 2014; **16**: 75–77.
774. Sharma R, Parker S, Al-Adhami B, Bachand N, Jenkins E. Comparison of tissues (heart vs. brain)and serological tests (MAT, ELISA and IFAT)for detection of *Toxoplasma gondii* in naturally infected wolverines (*Gulo gulo*) from the Yukon, Canada. *Food and Waterborne Parasitology* 2019; **15**. doi:10.1016/j.fawpar.2019.e00046.
775. Sharma R, Bush J, Tiwari A, Chikweto A, Bhaiyat M. Seroprevalence of *Toxoplasma gondii* in sheep and goats in Grenada, West Indies. *International Journal of Current Research and Academic Review* 2015; **3**: 262–267.
776. Shiibashi T, Narasaki K, Yoshida M, Nogami S. Prevalence of anti-*Toxoplasma gondii* antibody in hunter-killed wild boars, *Sus scrofa leucomystax*, on Amakusa Island, Kumamoto Prefecture, Japan. *Journal of Veterinary Medical Science* 2004; **66**: 327–328.
777. Shu F, Wu D, Zhou Z, Lv R, Yang J, Nie K *et al.* Seroprevalence of *Toxoplasma gondii* Infection in Slaughter Pigs in Sichuan, China. *Journal of Animal and Veterinary Advances* 2011; **10**: 1638–1639.
778. Siepierski SJ, Tanner CE, Embil JA. Prevalence of Antibody to *Toxoplasma gondii* in the Moose (*Alces alces americana Clinton*) of Nova Scotia, Canada. *Journal of Parasitology* 1990; **76**: 136–138.
779. Silva RC da, Machado GP, Cruvinel TM de A, Cruvinel CA, Langoni H. Detection of antibodies to *Toxoplasma gondii*in wild animals in Brazil. *Journal of Venomous Animals and Toxins Including Tropical Diseases* 2014; **20**: 1–4.
780. Silva RC da, Machado GP, Cruvinel TM de A, Cruvinel CA, Langoni H. Frequency of *Toxoplasma gondii* antibodies in tufted capuchin monkeys (*Cebus apella nigritus*) from an ecological station in the state of São Paulo, Brazil. *Pesquisa Veterinaria Brasileira* 2013; **33**: 251–253.
781. Silva V, Kramer B, Trevisol IM. Seroprevalence of *Toxoplasma gondii* and Hepatitis E virus (HEV) in free-ranging wild boars hunted in six Brazilian states. In: *26th International Pig Veterinary Society Congress*. Rio de Janeiro, Brazil, 2022.
782. Simon A, Chambellant M, Ward BJ, Simard M, Proulx JF, Levesque B *et al.* Spatio-temporal variations and age effect on *Toxoplasma gondii* seroprevalence in seals from the Canadian Arctic. *Parasitology* 2011; **138**: 1362–1368.
783. Simon A, Poulin MB, Rousseau AN, Dubey JP, Ogden NH. Spatiotemporal Dynamics Of *Toxoplasma gondii* Infection In Canadian Lynx (*Lynx canadensis*) In Western Québec, Canada. *Journal of Wildlife Diseases* 2013; **49**: 39–48.
784. Singh H, Tewari A, Mishra A, Maharana B, Sudan V, Raina O *et al.* Detection of antibodies to *Toxoplasma gondii* in domesticated ruminants by recombinant truncated SAG2 enzyme-linked immunosorbent assay. *Tropical Animal Health and Production* 2015; **47**: 171–178.
785. Siqueira DB, Aléssio FM, Mauffrey JF, Marvulo MFV, Ribeiro VO, Oliveira RL *et al.* Seroprevalence of *Toxoplasma gondii* in Wild Marsupials and Rodents from the Atlantic Forest of Pernambuco State, Northeastern Region, Brazil. *Journal of Parasitology* 2013; **99**: 1140–1143.
786. Sixl W, Köck M, Withalm H, Stünzner D. Serological investigations of the hedgehog (*Erinaceus europaeus*) in Styria. 2. Report. *Geographia medica Supplement = Geographia medica Sonderband* 1989; **2**: 105–8.
787. Slany M, Reslova N, Babak V, Lorencova A. Molecular characterization of *Toxoplasma gondii* in pork meat from different production systems in the Czech Republic. *International Journal of Food Microbiology*  2016; **238**: 252–255.
788. Smallbone WA, Chadwick EA, Francis J, Guy E, Perkins SE, Sherrard-Smith E *et al.* East-West Divide: Temperature and land cover drive spatial variation of *Toxoplasma gondii* infection in Eurasian otters (*Lutra lutra*) from England and Wales. *Parasitology* 2017; **144**: 1433–1440.
789. Smith DD, Frenkel JK. Prevalence of antibodies to *Toxoplasma gondii* in wild mammals of Missouri and east central Kansas: Biologic and ecologic considerations of transmission. *Journal of Wildlife Diseases* 1995; **31**: 15–21.
790. Smith HJ. Seroprevalence of anti-Toxoplasma IgG in Canadian swine. *Can J Vet Res* 1991; **55**: 380–381.
791. Smith KE, Zimmerman JJ, Patton S, Beran GW, Hill HT. The epidemiology of toxoplasmosis in Iowa swine farms with an emphasis on the roles of free-living mammals. *Veterinary Parasitology* 1992; **42**: 199–211.
792. Smith LN, Waltzek TB, Rotstein DS, Francis-Floyd R, Walsh MT, Wellehan JFX *et al.* Disseminated toxoplasmosis *Toxoplasma gondii* in a wild Florida manatee *Trichechus manatus latirostris* and seroprevalence in two wild populations. *Diseases of Aquatic Organisms* 2016; **122**: 77–83.
793. Soares H, Ahid S, Bezerra A, Pena H, Dias R, Gennari S. Prevalence of anti-*Toxoplasma gondii* and anti-*Neospora caninum* antibodies in sheep from Mossoro, Rio Grande do Norte, Brazil. *Veterinary Parasitology* 2009; **160**: 211–214.
794. Sobrino R, Cabezón O, Millán J, Pabón M, Arnal MC, Luco DF *et al.* Seroprevalence of *Toxoplasma gondii* antibodies in wild carnivores from Spain. *Veterinary Parasitology* 2007; **148**: 187–192.
795. Soccol V, de Castro E, Gazda T, Garcia G, Richartz R, Dittrich R. Occurrence of anti-*Toxoplasma gondii* antibodies in ovine from urban and periurban areas from Curitiba, Parana State. *Revista Brasileira de Parasitologia Veterinaria* 2009; **18**: 69–70.
796. Solorio MR, Gennari SM, Soares HS, Dubey JP, Hartley ACZ, Ferreira F. *Toxoplasma gondii* Antibodies in Wild White-Lipped Peccary (*Tayassu pecari*) From Peru. *Journal of Parasitology* 2010; **96**: 1232–1232.
797. Sousa S, Thompson G, Silva E, Freire L, Lopes D, da Costa J *et al.* Determination of the More Adequate Modified Agglutination Test Cut-off for Serodiagnosis of *Toxoplasma gondii* Infection in Sheep. *Zoonoses and Public Health* 2009; **56**: 252–256.
798. Souza LR, Carvalho MPN, Lopes CEB, Lopes MC, Campos BH, Teixeira ÉPT *et al.* Outbreak of canine distemper and coinfections in a maned wolf (*Chrysocyon brachyurus*) and in three giant anteaters (*Myrmecophaga tridactyla)*. *Braz J Microbiol* 2022; **53**: 1731–1741.
799. Spagnol F, Paranhos E, Oliveira L, de Medeiros S, Lopes C, Albuquerque G. Prevalence of antibodies anti-*Toxoplasma gondii* in slaughtered cattle at stockyards in the State of Bahia, Brazil. *Revista Brasileira De Parasitologia Veterinaria* 2009; **18**: 42–45.
800. Spencer JA, Higginbotham MJ, Blagburn BL. Seroprevalence of *Neospora caninum* and *Toxoplasma gondii* in Captive and Free-Ranging Nondomestic Felids in the United States. *Journal of Zoo and Wildlife Medicine* 2003; **34**: 246–249.
801. Spencer JA, Morkel P. Serological survey of sera from lions in Etosha National Park. *J Wild! Res* 1993; **23**: 60–61.
802. Špilovská S, Reiterová K, Kováčová D, Bobáková M, Dubinský P. The first finding of *Neospora caninum* and the occurrence of other abortifacient agents in sheep in Slovakia. *Veterinary Parasitology* 2009; **164**: 320–323.
803. Spriggs MC, Gerhold RW, Wilkes RP, Keenlance P, Sanders RL, Witt J *et al.* Serosurvey, Hematology, and Causes of Mortality of Free-Ranging American Martens (*Martes americana*) in Michigan. *Journal of Zoo and Wildlife Medicine* 2018; **49**: 371–383.
804. Sroka J, Bilska-Zajac E, Wójcik-Fatla A, Zajac V, Dutkiewicz J, Karamon J *et al.* Detection and Molecular Characteristics of *Toxoplasma gondii* DNA in Retail Raw Meat Products in Poland. *Foodborne Pathogens and Disease* 2019; **16**: 195–204.
805. Sroka J, Karamon J, Cencek T, Dutkiewicz J. Preliminary assessment of usefulness of cELISA test for screening pig and cattle populations for presence of antibodies against *Toxoplasma gondii*. *Annals of Agricultural and Environmental Medicine* 2011; **18**: 335–339.
806. Sroka J, Karamon J, Wójcik-Fatla A, Piotrowska W, Dutkiewicz J, Bilska-Zajac E *et al.* *Toxoplasma gondii* infection in slaughtered pigs and cattle in Poland: seroprevalence, molecular detection and characterization of parasites in meat. *Parasites & Vectors* 2020; **13**. doi:10.1186/s13071-020-04106-1.
807. Sroka J, Cencek T, Ziomko I, Karamon J, Zwolinski J. Preliminary Assessment of Elisa, Mat, and Lat For Detecting *Toxoplasma gondii* Antibodies In Pigs. *Bull Vet Inst Pulawy* 2008; **52**: 545–549.
808. Sroka J, Zwoliński J, Dutkiewicz J. Seroprevalence of *Toxoplasma gondii* in farm and wild animals from the area of Lublin province. *Bulletin of the Veterinary Institute in Pulawy* 2007; **51**: 535–540.
809. Standley WG, McCue PM. Prevalence of antibodies against selected diseases in San Joaquin kit foxes at Camp Roberts, California. Calif Fish Game. 1997; **83**: 30–37.
810. Steinparzer R, Reisp K, Grünberger B, Köfer J, Schmoll F, Sattler T. Comparison of Different Commercial Serological Tests for the Detection of *Toxoplasma gondii* Antibodies in Serum of Naturally Exposed Pigs. *Zoonoses and Public Health* 2015; **62**: 119–124.
811. Stensgaard AS, Sengupta ME, Chriel M, Nielsen ST, Petersen HH. Sero-prevalence and risk factors of *Toxoplasma gondii* infection in wild cervids in Denmark. *International Journal for Parasitology: Parasites and Wildlife* 2022; **17**: 288–294.
812. Stephenson N, Higley JM, Sajecki JL, Chomel BB, Brown RN, Foley JE. Demographic Characteristics and Infectious Diseases of a Population of American Black Bears in Humboldt County, California. *Vector-Borne and Zoonotic Diseases* 2015; **15**: 116–123.
813. Stewart RL, Humphreys JG, Dubey JP. *Toxoplasma gondii* Antibodies in Woodchucks (*Marmota monax*) from Pennsylvania. *The Journal of Parasitology* 1995; **81**: 126.
814. Stieve E, Beckmen K, Kania SA, Widner A, Patton S. *Neospora caninum* and *Toxoplasma gondii* antibody prevalence in Alaska wildlife. *Journal of Wildlife Diseases* 2010; **46**: 348–355.
815. Stutzin M, Contreras MC, Schenone H. Epidemiology of toxoplasmosis in Chile. V. Prevalence of the infection in humans and domestic and wild animals, studied by indirect hemagglutination reaction, in the Juan Fernández Archipelago. V Region. *Boletin chileno de parasitologia* 1989; **44**: 37–40.
816. Su R, Jiang N, Lu Y, Jian F, Wang H, Zhang G *et al.* Low prevalence of viable *Toxoplasma gondii* in swine from slaughter houses in the central of China. *Parasitology International* 2020; **76**. doi:10.1016/j.parint.2020.102090.
817. Suaréz-Aranda F, Galisteo AJ, Hiramoto RM, Cardoso RPA, Meireles LR, Miguel O *et al.* The prevalence and avidity of *Toxoplasma gondii* IgG antibodies in pigs from Brazil and Peru. *Vet Parasitol* 2000; **91**: 23–32.
818. Sudan V, Tewari A, Singh H. Serodiagnosis of *Toxoplasma gondii* infection in bovines from Kerala, India using a recombinant surface antigen 1 ELISA. *Biologicals* 2015; **43**: 250–255.
819. Sudan V, Tewari A, Singh H. Detection of Antibodies Against *Toxoplasma gondii* in Indian Cattle by Recombinant SAG2 Enzyme-Linked Immunosorbent Assay. *Acta Parasitologica* 2019; **64**: 148–151.
820. Sulzner K, Johnson CK, Bonde RK, Gomez NA, Powell J, Nielsen K *et al.* Health Assessment and Seroepidemiologic Survey of Potential Pathogens in Wild Antillean Manatees (*Trichechus manatus manatus*). *PLoS ONE* 2012; **7**: e44517.
821. Sun H, Wang Y, Zhang Y, Ge W, Zhang F, He B *et al.* Prevalence and Genetic Characterization of *Toxoplasma gondii* in Bats in Myanmar. *Applied and Environmental Microbiology* 2013; **79**: 3526–3528.
822. Sun H. Serological investigation of swine toxoplasmosis in Xinyang, Henan. *Prog Vet Med* 2016; **36**: 123–125.
823. Sun H. Seroprevalence of the *Toxoplasma gondii* infection in swine in Xinyang City, Henan Province. *Prog Vet Med* 2016; **37**: 123–125.
824. Sun, W., Meng, Q., Cong, W., Shan, X., Wang, C., & Qian, A. (2015). Herd-level prevalence and associated risk factors for *Toxoplasma gondii, Neospora caninum, Chlamydia abortus* and bovine viral diarrhoea virus in commercial dairy and beef cattle in eastern, northern and northeastern China. *Parasitology Research*, *114*(11), 4211–4218.
825. Sun L, Liang Q, Nie L, Hu X, Li Z, Yang J *et al.* Serological evidence of *Toxoplasma gondii* and *Neospora caninum* infection in black-boned sheep and goats in southwest China. *Parasitology International* 2020; **75**. doi:10.1016/j.parint.2019.102041.
826. Suzán G, Ceballos G. The Role of Feral Mammals on Wildlife Infectious Disease Prevalence in Two Nature Reserves within Mexico City Limits. *Journal of Zoo and* 2013; **36**: 479–484.
827. Swai ES, Schoonman L. A survey of zoonotic diseases in trade cattle slaughtered at Tanga city abattoir: a cause of public health concern. *Asian Pacific journal of tropical biomedicine* 2012; **2**: 55–60.
828. Tagel M, Lassen B, Viltrop A, Jokelainen P. Large-Scale Epidemiological Study on *Toxoplasma gondii* Seroprevalence and Risk Factors in Sheep in Estonia: Age, Farm Location, and Breed Associated with Seropositivity. *Vector borne and zoonotic diseases* 2019; **19**: 421–429.
829. Taggart PL, Fancourt BA, Fabijan J, Peacock DE, Speight KN, Caraguel CGB *et al.* No Evidence of *Toxoplasma gondii* Exposure in South Australian Koalas (*Phascolarctos cinereus)*. *Journal of Parasitology* 2019; **105**: 638.
830. Taggart PL. *Ecology of cat-borne parasitoses in Australia*. 2019. School of Animal and Veterinary Sciences. University of Adelaide, School of Animal & Veterinary Sciences
831. Taggart PL, Fancourt BA, Peacock D, Caraguel CGB, McAllister MM. Variation in *Toxoplasma gondii* seroprevalence: effects of site, sex, species and behaviour between insular and mainland macropods. *Wildlife Research* 2019. doi:10.1071/wr19041.
832. Tagwireyi W, Etter E, Neves L. Seroprevalence and associated risk factors of *Toxoplasma gondii* infection in domestic animals in southeastern South Africa. *Onderstepoort Journal of Veterinary Research* 2019; **86**. doi:10.4102/ojvr.v86i1.1688.
833. Tan Q, Yang X, Yin M, Hu L, Qin S, Wang J *et al.* Seroprevalence and correlates of *Toxoplasma gondii* infection in dairy cattle in northwest China. *Acta Parasitologica* 2015; **60**: 618–621.
834. Tao Q, Wang Z, Feng H, Fang R, Nie H, Hu M *et al.* Seroprevalence and Risk Factors For *Toxoplasma gondii* Infection On Pig Farms In Central China. *Journal of Parasitology* 2011; **97**: 262–264.
835. Tegegne D, Kelifa A, Abdurahaman M, Yohannes M. Seroepidemiology and associated risk factors of *Toxoplasma gondii* in sheep and goats in Southwestern Ethiopia. *BMC Veterinary Research* 2016; **12**. doi:10.1186/s12917-016-0906-2.
836. Thakur R, Sharma R, Aulakh R, Gill J, Singh B. Prevalence, molecular detection and risk factors investigation for the occurrence of *Toxoplasma gondii* in slaughter pigs in North India. *BMC Veterinary Research* 2019; **15**. doi:10.1186/s12917-019-2178-0.
837. Thiptara A, Kongkaew W, Bilmad U, Bhumibhamon T, Anan S. Toxoplasmosis in piglets. In: Blouin EF, Maillard JC (eds). *Impact of Emerging Zoonotic Diseases On Animal Health*. Blackwell Publishing: Oxford, 2006, pp 336–338.
838. Tialla D, Dahourou L, Gbati O. Seroprevalence and factors associated with bovine and swine toxoplasmosis in Bobo-Dioulasso, Burkina Faso. *Veterinary World* 2019; **12**: 1519–1523.
839. Tilahun B, Tolossa YH, Tilahun G, Ashenafi H, Shimelis S. Seroprevalence and Risk Factors of *Toxoplasma gondii* Infection among Domestic Ruminants in East Hararghe Zone of Oromia Region, Ethiopia. *Veterinary medicine international* 2018; **2018**: 4263470.
840. Timm SF, Munson L, Summers BA, Teno KA, Dubovi EJ, Rupprecht CE *et al.* A suspected canine distemper epidemic as the cause of a catastrophic decline in Santa Catalina Island foxes (*Urocyon littoraus cataunae*). *Journal of Wildlife Diseases* 2009; **45**: 333–343.
841. Tizard IR, Billett JB, Ramsden RO. The prevalence of antibodies against *Toxoplasma gondii* in some Ontario mammals. *Journal of Wildlife Diseases* 1976; **12**: 322–325.
842. Tizard IR, Harmeson J, Lai CH. The prevalence of serum antibodies to *Toxoplasma gondii* in Ontario mammals. *Canadian journal of comparative medicine : Revue canadienne de medecine comparee* 1978; **42**: 177–83.
843. Tocidlowski ME, Lappin MR, Summer PW, Stoskopf MK. Serologic survey for toxoplasmosis in river otters. *Journal of Wildlife Diseases* 1997; **33**: 649–652.
844. Tonouhewa ABN, Akpo Y, Sherasiya A, Sessou P, Adinci JM, Aplogan GL *et al.* A serological survey of *Toxoplasma gondii* infection in sheep and goat from Benin, West-Africa. *Journal of Parasitic Diseases : official organ of the Indian Society for Parasitology* 2019; **43**: 343–349.
845. Torres-Castro M, Noh-Pech H, Puerto-Hernández R, Reyes-Hernández B, Panti-May A, Hernández-Betancourt S *et al.* First molecular evidence of *Toxoplasma gondii* in opossums (*Didelphis virginiana*) from Yucatan, Mexico. *Open Veterinary Journal* 2016; **6**: 57–61.
846. Torres-Castro M, Muñoz-Dueñas D, Hernández-Betancourt S, Bolio-González M, Noh-Pech H, Peláez-Sánchez R *et al.* Infección con *Toxoplasma gondii* (Eucoccidiorida: Sarcocystidae) en murciélagos de Campeche y Yucatán, México. *Revista de Biología Tropical* 2019; **67**: 633–642.
847. Touloudi A, Valiakos G, Athanasiou LV, Birtsas P, Giannakopoulos A, Papaspyropoulos K *et al.* A serosurvey for selected pathogens in Greek European wild boar. *Veterinary Record Open* 2015; **2**: e000077.
848. Trevisani N, Vieira-Neto A, Güths MF, Aparecida A, de Souza AP, de Moura AB. *Toxoplasma gondii*: Sorologia E Fatores De Risco Em Suínos De. *Archives of Veterinary Science* 2013.
849. Trombley SN, Barthman-Thompson LM, Riley MK, Estrella SA, Smith KR, Clifford DL *et al.* Parasites of An Endangered Harvest Mouse (*Reithrodontomys raviventris halicoetes*) In A Northern California Marsh. *Journal of Wildlife Diseases* 2021; **58**: 122–136.
850. Truppel JH, Reifur L, Montiani-Ferreira F, Lange RR, Vilani RGDODC, Gennari SM *et al.* *Toxoplasma gondii* in Capybara (*Hydrochaeris hydrochaeris*) antibodies and DNA detected by IFAT and PCR. *Parasitology Research* 2010; **107**: 141–146.
851. Tryland M, Nymo IH, Nielsen O, Nordøy ES, Kovacs KM, Krafft BA *et al.* Serum Chemistry And Antibodies Against Pathogens In Antarctic Fur Seals, Weddell Seals, Crabeater Seals, and Ross Seals. *Journal of Wildlife Diseases* 2012; **48**: 632–645.
852. Tsokana CN, Sokos C, Giannakopoulos A, Birtsas P, Athanasiou LV, Valiakos G *et al.* Serological and molecular investigation of selected parasitic pathogens in European brown hare (*Lepus europaeus*) in Greece: inferring the ecological niche of *Toxoplasma gondii* and *Leishmania infantum* in hares. *Parasitology Research* 2019;**118** : 2715–2721.
853. Tuda J, Adiani S, Ichikawa-Seki M, Umeda K, Nishikawa Y. Seroprevalence of *Toxoplasma gondii* in humans and pigs in North Sulawesi, Indonesia. *Parasitology International* 2017; **66**: 615–618.
854. Turceková L, Antolová D, Reiterová K, Spisák F. Occurrence and genetic characterization of *Toxoplasma gondii* in naturally infected pigs. *Acta Parasitologica* 2013; **58**: 361–366.
855. Turčeková Ľ, Hurníková Z, Spišák F, Miterpáková M, Chovancová B. *Toxoplasma gondii* in protected wildlife in the Tatra National Park (TANAP), Slovakia. *Ann Agric Environ Med* 2014; **21**: 235–238.
856. Turni C, Smales LR. Parasites of the bridled nailtail wallaby (*Onychogalea fraenata*) (Marsupialia : Macropodidae). *Wildl Res* 2001; **28**: 403–411.
857. Tzanidakis N, Maksimov P, Conraths F, Kiossis E, Brozos C, Sotiraki S *et al.* *Toxoplasma gondii* in sheep and goats: Seroprevalence and potential risk factors under dairy husbandry practices. *Veterinary Parasitology* 2012; **190**: 340–348.
858. Ueno T, Gonçalves V, Heinemann M, Dilli T, Akimoto B, de Souza S *et al.* Prevalence of *Toxoplasma gondii* and *Neospora caninum* infections in sheep from Federal District, central region of Brazil. *Tropical Animal Health and Production* 2009; **41**: 547–552.
859. Uhart MM, Rago MV, Marull CA, Ferreyra H del V, Pereira JA. Exposure to selected pathogens in Geoffroy’s cats and domestic carnivores from central Argentina. *Journal of Wildlife Diseases* 2012; **48**: 899–909.
860. Ullmann LS, Gravinatti ML, Yamatogi RS, Santos LC dos, Moraes W de, Cubas ZS *et al.* Serosurvey of anti-Leptospira sp. and anti-*Toxoplasma gondii* antibodies in capybaras and collared and white-lipped peccaries. *Revista da Sociedade Brasileira de Medicina Tropical* 2017; **50**: 248–250.
861. USDA Animal and Plant Health Inspection Service. *Toxoplasma gondii* Antibody Seroprevalence in U.S. Swine. APHIS Info Sheet. 2018.
862. Uzelac A, Klun I, Ćirović D, Penezić A, Ćirković V, Djurković-Djaković O. Detection and genotyping of *Toxoplasma gondii* in wild canids in Serbia. *Parasitology International* 2019; **73**: 101973.
863. van der Giessen J, Fonville M, Bouwknegt M, Langelaar M, Vollema A. Seroprevalence of *Trichinella spiralis* and *Toxoplasma gondii* in pigs from different housing systems in The Netherlands. *Vet Parasitol* 2007; **148**: 371–374.
864. van Knapen F, Kremers AFT, Franchimont JH, Narucka U. Prevalence of antibodies to *Toxoplasma gondii* in cattle and swine in the Netherlands: Towards an integrated control of livestock production. *Veterinary Quarterly* 1995; **17**: 87–91.
865. Vanek JA, Dubey JP, Thulliez P, Riggs MR, Stromberg BE. Prevalence of *Toxoplasma gondii* Antibodies in Hunter-Killed White-Tailed Deer (*Odocoileus virginianus*) in Four Regions of Minnesota. *The Journal of Parasitology* 1996; **82**: 41.
866. Venturini MC, Bacigalupe D, Venturini L, Rambeaud M, Basso W, Unzaga JM *et al.* Seroprevalence of *Toxoplasma gondii* in sows from slaughterhouses and in pigs from an indoor and an outdoor farm in Argentina. *Vet Parasitol* 2004; **124**: 161–165.
867. Verhelst D, De Craeye S, Vanrobaeys M, Czaplicki G, Dorny P, Cox E. Seroprevalence of *Toxoplasma gondii* in domestic sheep in Belgium. *Veterinary Parasitology* 2014; **205**: 57–61.
868. Verin R, Mugnaini L, Nardoni S, Papini RA, Ariti G, Poli A *et al.* Serologic, molecular, and pathologic survey of *Toxoplasma gondii* infection in free-ranging red foxes (*Vulpes vulpes*) in central Italy. *Journal of Wildlife Diseases* 2013; **49**: 545–551.
869. Verma SK, Carstensen M, Calero-Bernal R, Moore SA, Jiang T, Su C *et al.* Seroprevalence, isolation, first genetic characterization of *Toxoplasma gondii*, and possible congenital transmission in wild moose from Minnesota, USA. *Parasitology Research* 2016; **115**: 687–690.
870. Verma SK, Sweeny AR, Lovallo MJ, Calero-Bernal R, Kwok OC, Jiang T *et al.* Seroprevalence, isolation and co-infection of multiple *Toxoplasma gondii* strains in individual bobcats (*Lynx rufus*) from Mississippi, USA. *International Journal for Parasitology* 2017; **47**: 297–303.
871. Veronesi F, Santoro A, Milardi G, Diaferia M, Branciari R, Miraglia D *et al.* Comparison of PCR assays targeting the multi-copy targets B1 gene and 529 bp repetitive element for detection of *Toxoplasma gondii* in swine muscle. *Food Microbiology* 2017; **63**: 213–216.
872. Vikøren T, Tharaldsen J, Fredriksen B, Handeland K. Prevalence of *Toxoplasma gondii* antibodies in wild red deer, roe deer, moose, and reindeer from Norway. *Veterinary Parasitology* 2004; **120**: 159–169.
873. Villagra-Blanco R, Barrantes-Granados O, Montero-Caballero D, Romero-Zúñiga J, Dolz G. Seroprevalence of *Toxoplasma gondii* and *Neospora caninum* infections and associated factors in sheep from Costa Rica. *Parasite Epidemiology and Control* 2019; **4**. doi:10.1016/j.parepi.2019.e00085.
874. Villari S, Vesco G, Petersen E, Crispo A, Buffolano W. Risk factors for toxoplasmosis in pigs bred in Sicily, Southern Italy. *Vet Parasitol* 2009; **161**: 1–8.
875. Vismarra A, Barilli E, Miceli M, Mangia C, Genchi M, Brindani F *et al.* *Toxoplasma gondii* in the Cornigliese sheep breed in Italy: Meat juice serology, *in vitro* isolation and genotyping. *Veterinary Parasitology* 2017; **243**: 125–129.
876. Vitaliano SN, Soares HS, Pena HF de J, Dubey JP, Gennari SM. Serologic Evidence of *Toxoplasma gondii* Infection in Wild Birds and Mammals From Southeast Brazil. *Journal of Zoo and Wildlife Medicine* 2014; **45**: 197–199.
877. Vostalová E, Literák I, Pavlásek I, Sedlák K. Prevalence of *Toxoplasma gondii* in finishing pigs in a large-scale farm in the Czech Republic. *Acta Vet BRNO* 2000; **69**: 209–212.
878. Waap H, Nunes T, Vaz Y, Leitão A. Serological survey of *Toxoplasma gondii* and *Besnoitia besnoiti* in a wildlife conservation area in southern Portugal. *Veterinary Parasitology: Regional Studies and Reports* 2016; **3–4**: 7–12.
879. Wallander C, Frössling J, Vågsholm I, Uggla A, Lundén A. *Toxoplasma gondii* seroprevalence in wild boars *(Sus scrofa)* in Sweden and evaluation of ELISA test performance. *Epidemiology & Infection* 2015; **143**: 1913–1921.
880. Wallander C, Frössling J, Dórea FC, Uggla A, Vågsholm I, Lundén A. Pasture is a risk factor for *Toxoplasma gondii* infection in fattening pigs. *Veterinary Parasitology* 2016; **224**: 27–32.
881. Walton BC, Walls KW. Prevalence of Toxoplasmosis in Wild Animals From Fort Stewart, Georgia, As Indicated By Serological Tests and Mouse Inoculation. *The American journal of tropical medicine and hygiene* 1964; **13**: 530–533.
882. Wang D, Liu Y, Jiang T, Zhang G, Yuan G, He J *et al.* Seroprevalence and genotypes of *Toxoplasma gondii* isolated from pigs intended for human consumption in Liaoning province, northeastern China. *Parasites & Vectors* 2016; **9**. doi:10.1186/s13071-016-1525-2.
883. Wang H, Wang T, Luo Q, Huo X, Wang L, Liu T *et al.* Prevalence and genotypes of *Toxoplasma gondii* in pork from retail meat stores in Eastern China. *International Journal of Food Microbiology*  2012; **157**: 393–397.
884. Wang J, Shi D, Cheng H, Huo J. Serological survey of toxoplasmosis of cattle in Zhenzhou City. *Chinese Journal of Veterinary Medicine* 2013; **49**: 39–40.
885. Wang M, Ye Q, Zhang N, Zhang D. Seroprevalence of *Toxoplasma gondii* infection in food-producing animals in Northwest China. *Chinese Journal of Zoonoses* 2016; **32**: 608–612.
886. Wang W, Zhang J, Chen W, Wang J, Meng Q, Qiao J. Serological survey of Toxoplasma infection among human and animals in Shihezi Region. *Progress in Veterinary Medicine* 2011; **32**: 120–122.
887. Wang X-L, Dong L, Zhang L, Lv Y, Li Q, Li H-L. Seroprevalence and Genetic Characterization of *Toxoplasma gondii* In Naturally Infected Synanthropic Rodents In Yunnan Province, Southwestern China. *Journal of Parasitology* 2018; **104**: 383–387.
888. Wang X, Li X, Cairang N, Wang G, Ma L. Serological detection of abortion disease in Qura-type of Tibetan sheep in Henan County of Qinghai. *Qinghai J Anim Vet Sci* 2015; **45**: 8–10.
889. Wang Y, Gui B, Li R, Wang G, Ge M, Liu G. Seroprevalence and Risk Factors of *Toxoplasma gondii* Infection in Growth Stages of Pigs in Hunan Province, Subtropical China. *Vector borne and zoonotic diseases* 2019; **19**: 945–949.
890. Wang Z, Li K, Sun Y, Li H, Yang H, Dong C *et al.* Sero-epidemiological investigation and risk factor analysis of *Toxoplasma gondii* from cattle in Chongqing. *Journal of Southwest University (Natural Science Edition)* 2018; **40**: 8–12.
891. Wanha K, Edelhofer R, Gabler-Eduardo C, Prosl H. Prevalence of antibodies against *Neospora caninum* and *Toxoplasma gondii* in dogs and foxes in Austria. *Veterinary Parasitology* 2005; **128**: 189–193.
892. Watts DE, Benson AM. Prevalence of antibodies for selected canine pathogens among wolves (*Canis lupus*) from the Alaska Peninsula, USA. *Journal of Wildlife Diseases* 2016; **52**: 506–515.
893. Wen Q, Guo Y, Yang J, Shen H. Investigation on epidemiology of toxoplasmosis in pig from Henan Province. *Chinese Journal of Veterinary Medicine* 2015; **51**: 44–45.
894. White CL, Schuler KL, Thomas NJ, Webb JL, Saliki JT, Ip HS *et al.* Pathogen exposure and blood chemistry in the Washington, USA population of northern sea otters (*Enhydra lutris kenyoni*). *Journal of Wildlife Diseases* 2013; **49**: 887–899.
895. Wiengcharoen, J., Nakthong, C., Mitchaothai, J., Udonsom, R., & Sukthana, Y. (2012). Toxoplasmosis And Neosporosis Among Beef Cattle Slaughtered For Food In Western Thailand. *Southeast Asian Journal Of Tropical Medicine And Public Health*, *43*(5), 1087–1093.
896. Winter M, Abate SD, Pasqualetti M, Farina FA, Ercole ME, Pardini L *et al.* *Toxoplasma gondii* and *Trichinella* infections in wild boars *(Sus scrofa)* from Northeastern Patagonia, Argentina. *Prev Vet Med* 2019; **168**: 75–80.
897. Witkowski L, Czopowicz M, Nagy DA, Potarniche AV, Aoanei MA, Imomov N *et al.* Seroprevalence of *Toxoplasma gondii* in wild boars, red deer and roe deer in Poland. *Parasite* 2015; **22**: 17.
898. Wolfe A, Hogan S, Maguire D, Fitzpatrick C, Vaughan L, Wall D *et al.* Red foxes (*Vulpes vulpes*) in Ireland as hosts for parasites of potential zoonotic and veterinary significance. *Veterinary Record* 2001; **149**: 759–763.
899. Wong A, Lanyon JM, O’Handley R, Linedale R, Woolford L, Long T *et al.* Serum antibodies against *Toxoplasma gondii* and *Neospora caninum* in southeast Queensland dugongs. *Marine Mammal Science* 2020; **36**: 180–194.
900. Wu D, Lv R, Sun X, Shu F, Zhou Z, Nie K *et al.* Seroprevalence of *Toxoplasma gondii* antibodies from slaughter pigs in Chongqing, China. *Trop Anim Health Prod* 2012; **44**: 685–687.
901. Wu F, Wang Y, Yang Z, Li X, Li Z, Lin Q. Seroprevalence and Risk Factors of *Toxoplasma gondii* in Slaughter Pigs in Shaanxi Province, Northwestern China. *Vector borne and zoonotic diseases* 2017; **17**: 517–519.
902. Wu J-Y, Li J-J, Wang D-F, Wei Y-R, Meng X-X, Tuerxun G *et al.* Seroprevalence of Five Zoonotic Pathogens in Wild Ruminants in Xinjiang, Northwest China. *Vector-Borne and Zoonotic Diseases* 2020; **20**: 882–887.
903. Wu S, Ciren D, Huang S, Xu M, Ga G, Yan C *et al.* First Report of *Toxoplasma gondii* Prevalence in Tibetan Pigs in Tibet, China. *Vector borne and zoonotic diseases* 2012; **12**: 654–656.
904. Wu S, Danba C, Huang S, Zhang D, Chen J, Gong G *et al.* Seroprevalence of *Toxoplasma gondii* Infection in Tibetan Sheep in Tibet, China. *Journal of Parasitology* 2011; **97**: 1188–1189.
905. Wyrosdick HM, Gerhold R, Su C, Mignucci-Giannoni AA, Bonde RK, Chapman A *et al.* Investigating seagrass in *Toxoplasma gondii* transmission in Florida (*Trichechus manatus latirostris*) and Antillean (*T. m. manatus*) manatees. *Diseases of Aquatic Organisms* 2017; **127**: 65–69.
906. Xiang Z, Duan Y, Yue X, Chen P. Serological investigation of swine toxoplasmosis in partial area of Anhui Province. *Hunan Agric Sci Technol* 2011; **17**: 138.
907. Xing B. Serological survey of toxoplasmosis in aborted cows in Yindu District, Anyang. *China Dairy* 2019; **211**: 55–56.
908. Xu B, Zhang X, Zeng Z, Feng C. Serological investigation and analysis of porcine toxoplasmosis in Chongqing. *Chinese Veterinary Science* 2013; **43**: 98–102.
909. Xu M, Liu Q, Fu J, Nisbet A, Shi D, He X *et al.* Seroprevalence of *Toxoplasma gondii* and *Neospora caninum* infection in dairy cows in subtropical southern China. *Parasitology* 2012; **139**: 1425–1428.
910. Xu P, Cai Y, Leng X, Wang J, Ma W, Mu G *et al.* Seroprevalence of *Toxoplasma gondii* infection in pigs in Jilin Province, Northeastern China. *Tropical Biomedicine* 2015; **32**: 116–120.
911. Xu Y, Li R, Liu G, Cong W, Zhang X, Yu X *et al.* Seroprevalence of *Toxoplasma gondii* Infection in Sows in Hunan Province, China. *Scientific World Journal* 2014. doi:10.1155/2014/347908.
912. Yai LEO, Ragozo AMA, Aguiar DM, Damaceno JT, Oliveira LN, Dubey JP *et al.* Isolation of *Toxoplasma gondii* from Capybaras (*Hydrochaeris hydrochaeris*) from São Paulo State, Brazil. *Journal of Parasitology* 2008; **94**: 1060–1063.
913. Yamakawa AC, Pellizzaro M, Joaquim SF, da Cunha GR, Weckerlin P, Martins CM *et al.* Serosurvey of *Toxoplasma gondii* and Leptospira spp. in Free-Range Agoutis (*Dasyprocta azarae*) from an Urban Area of Southern Brazil. *Journal of Wildlife Diseases* 2019; **56**: 472–474.
914. Yan X, Han W, Wang Y, Zhang H, Gao Z. Seroprevalence of *Toxoplasma gondii* infection in sheep in Inner Mongolia Province, China. *Parasite* 2020; **27**. doi:10.1051/parasite/2020008.
915. Yang N, Li H, He J, Mu M, Yang S. Seroprevalence of *Toxoplasma gondii* Infection in Domestic Sheep in Liaoning Province, Northeastern China. *Journal of Parasitology* 2013; **99**: 174–175.
916. Yang N, Xing M, Wang H, Li D, Wu Y. Detection and analysis of *Toxoplasma gondii* antibody from pigs sera in Liaoning Province. *Prog Vet Med* 2017; **38**: 125–128.
917. Yang P, Guo H, Tang Y, Wang J, Chen M, Li Z. Characteristic of Setting of Carbon Fiber Composite Grout. In: Deng W (ed). *Tongji University*. 2011, pp 229–232.
918. Yang S. *Seroepidemiological investigation of Chlamydia, Toxoplasma gondii and Neospora caninum in Yanbian cattle*. 2018.
919. Yang Y, Feng Y, Yao Q, Wang Y, Lu Y, Liang H *et al.* Seroprevalence, Isolation, Genotyping, and Pathogenicity of *Toxoplasma gondii* Strains from Sheep in China. *Frontiers In Microbiology* 2017; **8**. doi:10.3389/fmicb.2017.00136.
920. Ybañez R, Busmeon C, Viernes A, Langbid J, Nuevarez J, Ybañez A *et al.* Endemicity of Toxoplasma infection and its associated risk factors in Cebu, Philippines. *PLOS ONE* 2019; **14**. doi:10.1371/journal.pone.0217989.
921. Yildiz K, Kul O, Babur C, Kilic S, Gazyagci AN, Celebi B *et al.* Seroprevalence of *Neospora caninum* in dairy cattle ranches with high abortion rate: Special emphasis to serologic co-existence with *Toxoplasma gondii*, Brucella abortus and Listeria monocytogenes. *Vet Parasitol* 2009; **164**: 306–310.
922. Yin M, Wang J, Huang S, Qin S, Zhou D, Liu G *et al.* Seroprevalence and risk factors of *Toxoplasma gondii* in Tibetan Sheep in Gansu province, Northwestern China. *BMC Veterinary Research* 2015; **11**. doi:10.1186/s12917-015-0358-0.
923. Younis E, Abou-Zeid N, Zakaria M, Mahmoud M. Epidemiological studies on toxoplasmosis in small ruminants and equine in Dakahlia Governorate, Egypt. *Assiut Vet Med J* 2015; **61**: 22–31.
924. Yu B, Yin C, Yu X, AI M, Li X, Xue S *et al.* Preliminary investigation on the infection of *Toxoplasma gondii* in Yanbian area. *Jilin Agriculture* 2018; **30**: 80–71.
925. Yu H, Zhang Z, Liu Z, Qu D, Zhang D, Zhang H *et al.* Seroprevalence of *Toxoplasma gondii* infection in pigs, in Zhejiang Province, China. *J Parasitol* 2011; **97**: 748–749.
926. Yuan Z-G, Luo S-J, Dubey JP, Zhou D-H, Zhu Y-P, He Y *et al.* Serological Evidence of *Toxoplasma gondii* Infection in Five Species of Bats in China. *Vector-Borne and Zoonotic Diseases* 2013; **13**: 422–424.
927. Yucel S, Yaman M, Kurt C, Babur C, Celebi B, Kilic S *et al.* Seroprevalance of Brucellosis, Listeriosis and Toxoplasmosis in cattle in Adana province of Turkey. *Turkiye parazitolojii dergisi* 2014; **38**: 91–6.
928. Zanatto D, Gatto I, Labruna M, Jusi M, Samara S, Machado R *et al.* *Coxiella burnetii* associated with BVDV (Bovine Viral Diarrhea Virus), BoHV (Bovine Herpesvirus), Leptospira spp., *Neospora caninum*, *Toxoplasma gondii* and Trypanosoma vivax in reproductive disorders in cattle. *Revista Brasileira de Parasitologia Veterinaria* 2019; **28**: 245–257.
929. Zanet S, Palese V, Trisciuoglio A, Cantón Alonso C, Ferroglio E. *Encephalitozoon cuniculi*, *Toxoplasma gondii* and *Neospora caninum* infection in invasive Eastern Cottontail Rabbits *Sylvilagus floridanus i*n Northwestern Italy. *Veterinary Parasitology* 2013; **197**: 682–684.
930. Zarnke RL, Dubey JP, Hoef JMV, McNay ME, Kwok OCH. Serologic survey for *Toxoplasma gondii* in lynx from interior Alaska. *Journal of Wildlife Diseases* 2001; **37**: 36–38.
931. Zarnke RL, Dubey JP, Kwok OCH, Hoef JMV. Serologic survey for *Toxoplasma gondii* in grizzly bears from Alaska. Journal of Wildlife Diseases. 1997; **33**: 267–270.
932. Zarnke RL, Dubey JP, Kwok OCH, Ver Hoef JM. Serologic survey for *Toxoplasma gondii* in selected wildlife species from Alaska. *Journal of Wildlife Diseases* 2000; **36**: 219–224.
933. Zetun CB, Hoffmann JL, Silva RC, Souza LC, Langoni H. Leptospira spp. and *Toxoplasma gondii* antibodies in vampire bats (*Desmodus rotundus*) in Botucatu region, SP, Brazil. *Journal of Venomous Animals and Toxins Including Tropical Diseases* 2009; **15**: 546–552.
934. Zhang N, Wang S, Wang D, Li C, Zhang Z, Yao Z *et al.* Seroprevalence of *Toxoplasma gondii* infection and risk factors in domestic sheep in Henan province, central China. *Parasite* 2016; **23**. doi:10.1051/parasite/2016064.
935. Zhang SY, Jiang SF, He YY, Pan CE, Zhu M, Wei MX. Serologic Prevalence of *Toxoplasma gondii* in Field Mice, *Microtus fortis*, From Yuanjiang, Hunan Province, People’s Republic of China. *Journal of Parasitology* 2004; **90**: 437–438.
936. Zhang Y, Gong H, Mi R, Huang Y, Han X, Xia L *et al.* Seroprevalence of *Toxoplasma gondii* infection in slaughter pigs in Shanghai, China. *Parasitology International* 2020; **76**. doi:10.1016/j.parint.2020.102094.
937. Zhang Y, Ma X, Liu H, Dou Y, Wang R, Zhao W. Diagnosis and prevention of listeriosis in sheep. *Proc Confe Chin Soc Vet Sheep Prod Res in 2012* 2012; 355–356.
938. Zhao J, Tao J. Investigation report of major epidemic diseases in large-scale beef farms. *Today Animal Husbandry and Veterinary Medicine* 2018; **34**: 1–2.
939. Zhao P, Zhang S, Jia L, Yu L, Li N, Li J. Serological investigation of bovine toxoplasmosis in Delingha area of Qinghai Province. *Animal Husbandry & Veterinary Medicine* 2016; **48**: 146–147.
940. Zhao Z, He Q, Yang J, Li G, Gu D, Zhang C *et al.* Serological investigation of toxoplasmosis in cattle and sheep in some towns in Datong County. *Journal of Animal and Veterinary Sciences* 2018; **48**: 42–46.
941. Zheng H, Zhai S, Zhou X, Xu D. Serological investigation of swine toxoplasmosis in Rongchang area. *Shanghai Anim Husbandry Vet Commun* 2009; **6**: 39–40.
942. Zheng W-B, Gui B-Z, Long H-B, Chen Y-W, Zhu X-Q, Wang S-L *et al.* Molecular Detection and Genotyping of *Toxoplasma gondii* in Edward’s Long-Tailed Rats (*Leopoldamys edwardsi*). *Foodborne Pathogens and Disease* 2019; **16**: 539–542.
943. Zhou D, Zhao F, Lu P, Xia H, Xu M, Yuan L *et al.* Seroprevalence of *Toxoplasma gondii* infection in dairy cattle in southern China. *Parasites & Vectors* 2012; **5**. doi:10.1186/1756-3305-5-48.
944. Zhou D-H, Liang R, Yin C-C, Zhao F-R, Yuan Z-G, Lin R-Q *et al.* Seroprevalence of *Toxoplasma gondii* in Pigs From Southern China. *Journal of Parasitology* 2010; **96**: 673–674.
945. Zhou M, Cao S, Sevinc F, Sevinc M, Ceylan O, Liu M *et al.* Enzyme-linked immunosorbent assays using recombinant TgSAG2 and NcSAG1 to detect *Toxoplasma gondii* and *Neospora caninum*-specific antibodies in domestic animals in Turkey. *Journal of Veterinary Medical Science* 2016; **78**: 1877–1881.
946. Zhou P, Mu G, Li J, Li L, Ma Q, Yuan Y *et al.* Serological investigation of bovine toxoplasmosis in Delingha area of Qinghai Province. *Animal Husbandry & Veterinary Medicine* 2011; **43**: 103–104.
947. Zhou P, Nie H, Zhang L, Wang H, Yin C, Su C *et al.* Genetic Characterization of *Toxoplasma gondii* Isolates From Pigs in China. *Journal of Parasitology* 2010; **96**: 1027–1029.
948. Zitelli LC, Webster A, Morel AP, Umeno KA, Padilha TC, Rocha MA *et al.* Serological investigation of protozoan pathogens (*Trypanosoma cruzi*, *Toxoplasma gondii* and *Neospora caninum*) in opossums from southern Brazil. *Veterinary Parasitology: Regional Studies and Reports* 2021; **24**: 100546.
949. Zou F, Yu X, Yang Y, Hu S, Chang H, Yang J *et al.* Seroprevalence and Risk Factors of *Toxoplasma gondii* Infection in Buffaloes, Sheep and Goats in Yunnan Province, Southwestern China. *Iranian Journal of Parasitology* 2015; **10**: 648–651.
950. Zou F-C, Sun X-T, Xie Y-J, Li B, Zhao G-H, Duan G *et al.* Seroprevalence of *Toxoplasma gondii* in pigs in Southwestern China. *Parasitology International* 2009; **58**: 306–307.
