# Supplementary material for: The role of species ecology in predicting Toxoplasma gondii prevalence in wild and domesticated mammals globally
Source: PLoS Pathog. 2024 Jan 10;20(1):e1011908. doi: 10.1371/journal.ppat.1011908 (PMC10805296; doi:10.1371/journal.ppat.1011908)
Supplement: Attachment — Submitted filename: Response to Reviewer.docx [file ppat.1011908.s005.docx]

Reviewer #1: Below are comments, questions, and suggested edits.

**Comment 1**. Lines 28 and 99: Toxoplasma oocysts are not “free-living” but can survive for long periods of time in the environment.

**Response:** Change made.

Line 28 now reads: “ *Toxoplasma gondii* infects its hosts through oocysts in the environment…”

Line 98 now reads: “Infected felids excrete free-living oocysts in their feces…”

All other references to oocysts in this manner have been removed.

**Comment 2.** Lines 31, 41 and 50: I don’t think that “Susceptibility” is the correct word here. Susceptibility has to do with how susceptible an animal/person is to infection and associated disease and may be attributed to factors such as host immunologic status, host-parasite adaptation, etc. But this study does not examine the clinical aspects of infections. Consider reword to “exposure and infection”.

**Response:** Change made.

Lines 29-31 now read: “ These routes of infection enable specific predictions regarding the ecological and life history traits that should predispose specific taxa to higher exposure and, thus, infection rates of *T. gondii.*”

Lines 39-41 now read: “Among ecological guilds, terrestrial species had lower T. gondii prevalence than aquatic species, with freshwater aquatic taxa having an increased prevalence compared to marine aquatic species.

Lines 50-51 now read: “... enabling us to confirm the increased prevalence within aquatic and carnivorous species…”

**Comment 3.** Line 50: What is meant by “confirm”? Is this published elsewhere?

**Response:** Change made

Line 50 now reads “...demonstrating an increased infection risk for aquatic and carnivorous species.” We meant to relay that smaller studies have demonstrated carnivory to be a risk factor. We cite these papers in line 249.

**Comment 4.** Line 61: Toxoplasma does not cause severe health problems in most of the species it infects. Reword. Might instead write, “capable” of causing…

**Response:** Change made

Line 61 now reads: “...capable of causing severe and chronic health problems to host species,…”

**Comment 5**. Line 86: Suggest alternative word to “benefit”, such as relevance or importance.

**Response:** Change made

Lines 85-87 now read: “...Generalist pathogens with domestic animal reservoirs that affect multiple species, including humans, are particularly poignant research targets, given the relevance for human, wildlife and domestic animal health .”

**Comment 6.** Line 144: “Toxoplasmosis” Is the disease manifested from the infection. Suggest change word to transmission.

**Response:** Change made

Lines 141-143 now read: “Waterborne transmission is a significant exposure route for all mammals irrespective of diet, due to the risk of oocyst ingestion by drinking water and active foraging [28].”

**Comment 7.** Lines 143-147: Suggest rewording these sentences to more effectively make your point that although waterborne transmission is a significant route in terrestrial mammals, it is predicted that aquatic mammals would have a higher rate of waterborne exposure and therefore higher prevalence of infection than terrestrial mammals.

**Response:** Change made

Lines 143- 146 now read: “However, aquatic species are immersed in water, placing them at an increased risk of encountering oocysts which can be distributed throughout the water column, providing a multi-dimensional exposure surface. This risk is exacerbated by the ability of *T. gondii* to concentrate on aquatic particulates, sediment, biofilms and filter-feeding aquatic prey items [9].”

**Comment 8.** Line 148: Add “oocysts” so that sentence reads: Since T. gondii oocysts enter the aquatic system from…

**Response:** Change made

Lines 148-150 now read: “Since *T. gondii* oocysts enter the aquatic system from terrestrial pollution pathways (i.e., surface runoff and preferential flow) [29],..”

**Comment 9.** Can the authors comment on the varying methods used to determine Toxoplasma infection for prevalence calculations (e.g., MAT, PCR, etc.) and how the differences in sensitivity and specificity of the different test methods used across different studies included impact the model? In particular, assay validation in wildlife species.

**Response:** We have now included a paragraph discussing the statistical differences we found for prevalence estimates from studies using serology or isolation methods and provide some discussion on the cause and impact of methodological differences.

Lines 304-315 now read: “We did find that studies using serosurveys were generally associated with a higher prevalence. Although serological tests, especially the modified agglutination test, have been widely used in wildlife, these tests are rarely validated, such that false positives and negatives are possible [5]. Similarly, molecular or isolation methods require that bradyzoites or tachyzoites are included in the sampled tissue from infected individuals. In cases where infected individuals have low numbers of tissue cysts or in larger-bodied animals, there is an increased risk of sampling error, leading to an underestimation of prevalence. However, if the sample includes the life-stage, molecular methods can have high sensitivity and specificity [5]. Although our model controlled for methodological differences, the presence of a phylogenetic signal suggests that our models did not capture some phylogenetically conserved but influential ecological or physiological traits, which may also include taxon-specific differences in method sensitivity and specificity.”

**Comment 10.** Figure 1: Do dark grey areas correspond to regions where no data is available? Suggest including statement in figure legend to describe the dark grey regions.

**Response**: Yes, that is correct. We have now made this change.

Figure 1 legend now reads: “Countries for which *T. gondii* prevalence data was not found within the scope of our search are shown in grey.”

**Comment 11.** S1 and S2 Tables: Suggest including the total number of each order and taxonomic family included in the prevalence estimation.

**Response:** Change made.

The total number of individuals used in calculations are now included for the taxonomic order and family. We also included the number of species for each family.

**Comment 12**. Will the master dataset and list of publications included in the analysis be provided?

**Response**: Yes, the master dataset and the master list of publications used in the analysis are now also provided and uploaded in the supplementary information.
